# Counter-intuitive influence of Himalayan river morphodynamics on Indus Civilisation urban settlements

**DOI:** 10.1038/s41467-017-01643-9

**Published:** 2017-11-28

**Authors:** Ajit Singh, Kristina J. Thomsen, Rajiv Sinha, Jan-Pieter Buylaert, Andrew Carter, Darren F. Mark, Philippa J. Mason, Alexander L. Densmore, Andrew S. Murray, Mayank Jain, Debajyoti Paul, Sanjeev Gupta

**Affiliations:** 1 Department of Earth Sciences, Indian Institute of Technology Kanpur, Kanpur, 208016 India; 2 Department of Earth Science and Engineering, Imperial College London, London, SW7 2AZ UK; 3 Centre for Nuclear Technologies, Technical University of Denmark, DTU Risø Campus, DK-4000 Roskilde, Denmark; 4 Nordic Laboratory for Luminescence Dating, Department of Geoscience, Aarhus University, DTU Risø Campus, DK-4000 Roskilde, Denmark; 5 Department of Earth and Planetary Sciences, Birkbeck, University of London, London, WC1E 7HX UK; 6 Natural Environment Research Council Argon Isotope Facility, Scottish Universities Environmental Research Centre, Glasgow, G75 0QF UK; 7 Department of Earth and Environmental Science, University of St Andrews, St Andrews, KY16 9AJ UK; 8 Institute of Hazard, Risk, and Resilience and Department of Geography, Durham University, Durham, DH1 3LE UK

## Abstract

Urbanism in the Bronze-age Indus Civilisation (~4.6–3.9 thousand years before the present, ka) has been linked to water resources provided by large Himalayan river systems, although the largest concentrations of urban-scale Indus settlements are located far from extant Himalayan rivers. Here we analyse the sedimentary architecture, chronology and provenance of a major palaeochannel associated with many of these settlements. We show that the palaeochannel is a former course of the Sutlej River, the third largest of the present-day Himalayan rivers. Using optically stimulated luminescence dating of sand grains, we demonstrate that flow of the Sutlej in this course terminated considerably earlier than Indus occupation, with diversion to its present course complete shortly after ~8 ka. Indus urban settlements thus developed along an abandoned river valley rather than an active Himalayan river. Confinement of the Sutlej to its present incised course after ~8 ka likely reduced its propensity to re-route frequently thus enabling long-term stability for Indus settlements sited along the relict palaeochannel.

## Abstract (web summary)

The Bronze-age Indus civilisation (4.6–3.9 ka) was thought to have been linked to the development of water resources in the Himalayas. Here, the authors show that along the former course of the Sutlej River the Indus settlements developed along the abandoned river valley rather than an active Himalayan river.

## Introduction

Alluvial landscapes built by large perennial rivers form the environmental templates on which the earliest urban societies nucleated^1, 2^. Large-scale spatiotemporal settlement patterns in early urban societies are postulated to have been influenced by river migration across alluvial floodplains^1, 3, 4^. On long time scales, rivers migrate by episodic, relatively abrupt changes in their course called avulsions^5^. Avulsions lead to diversion of river flow into new or abandoned channel pathways on floodplains^5–7^. They are stochastic events that typically occur at century to millennial timescales^8^. A rare natural observation of such an event occurred in August 2008 on the Kosi River in the eastern Ganges Plains in northern India^9–11^. A levee breach caused the temporary re-routing of the Kosi River some 60 km eastwards into a former channel course that had been abandoned a hundred years previously, causing extensive flooding and loss of life in the region^9^. River avulsions have long been considered important in the development of early complex society^3, 4^, but their precise influence on early urban settlement patterns is poorly understood. It is commonly accepted that settlements are clustered near active rivers and that river avulsion leads to settlement abandonment^3^; this has been offered as an explanation for spatiotemporal changes in urban settlement patterns^4, 12, 13^, but this mechanism cannot be tested, unless the timing of major avulsions is known. Here we reconstruct the chronology of a major late Quaternary avulsion in the Himalayan foreland and evaluate its role in urban settlement patterns of the Bronze-age Indus Civilisation (~4.6–3.9 ka B.P.).

**Fig. 1:**
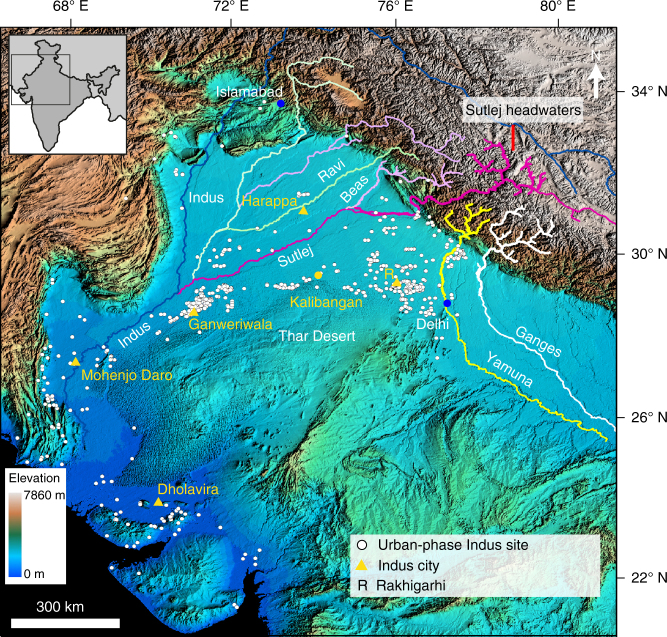
Topographic map showing northwestern India and Pakistan, key Himalayan rivers and the distribution of urban-phase Indus Civilisation settlements. Note how Indus urban-phase settlements are not necessarily located along modern Himalayan river courses. The most prominent cluster of sites occurs located on the drainage divide between the Sutlej and Yamuna rivers, an area devoid of perennial Himalayan drainage. Base digital elevation map is derived from NASA Shuttle Radar Topography Mission (SRTM)^53^. Site locations are from the compilation of urban-phase Indus settlement locations collated in Possehl^86^. Inset locates figure in south Asia

During the early- to mid-third millennium BCE, the Indus Civilisation developed one of the most extensive urban cultures in the Old World^14–16^. This civilisation was established on the alluvial plains of the Indo–Gangetic basin in northwestern India and Pakistan, with an urban-phase commencing ~4.6–4.5 ka B.P.^15, 17^. It was contemporaneous with and more extensive in area than the earliest urban societies of Egypt and Mesopotamia, encompassing an area estimated at ~1 million km^2^ ^14^. Urbanism in the Indus Civilisation is associated with the development of five large settlements considered by archaeologists as cities, and numerous smaller urban settlements that are characterised by distinctive architectural elements and material culture^15, 16, 18^. The Indus Civilisation has long been considered river-based, with two of its largest and best-known cities, Harappa and Mohenjo-Daro, located adjacent to large perennial Himalayan rivers^19, 20^. Indus settlements have also been shown to be associated with a sinuous palaeochannel inferred to be the ancient course of the Beas river in north-eastern Pakistan^20–22^. However, the largest concentration of Indus settlements is located near the divide between the Ganges–Yamuna and Indus river systems in India and Pakistan, far from major active rivers^14–16, 23–26^ (Fig. 1). Why numerous Indus settlements should have been located in a region now devoid of large perennial rivers has been the subject of vigorous debate and controversy.

During the late 19th century, topographers identified the trace of a major palaeochannel extending across the modern states of Punjab, Haryana and Rajasthan in India, and Cholistan in Pakistan^27–30^ (see Chakrabarti and Saini^25^ for review). Later, surveys revealed the presence of numerous archaeological sites spatially associated with this palaeochannel, many of which were shown to be urban settlements occupied during the peak of the Indus civilisation^24, 26, 31, 32^. The subsequent identification of this palaeochannel, known as the Ghaggar in India and the Hakra in Pakistan, on satellite imagery^33–36^ has led to intense discussion about its origin and its genetic link with nearby Indus settlements^12, 25, 37–40^. The Ghaggar–Hakra palaeochannel has been claimed as the former course of a large Himalayan river that provided water resources to sustain these Indus settlements^12, 33, 41, 42^, which include important sites such as Kalibangan, Banawali, Bhirrana and Kunal. Moreover, the palaeochannel has been linked with the mythical Sarasvati River first referred to in Vedic texts^12, 28–30, 41^. The modern landscape, by contrast, is characterised by ephemeral river courses, such as the Ghaggar River, which primarily flow during monsoon precipitation^39, 43, 44^.

The drying up of the river that formed the Ghaggar–Hakra palaeochannel has been suggested as a major factor in the decline and abandonment of Indus urban centres in the region from ~4.0–3.9 ka B.P.^14^. This has led to speculation that drying of the river also contributed to the transformation or collapse of the Indus urban system^24, 37, 41, 42^. For about a millennium after the decline of Indus urbanism, no large-scale urban centres developed in South Asia, until the early Historic period^15, 18^. The disappearance of the river has been explained as a consequence of river diversion related to tectonic activity^12^, or aridification due to climate change^39^. However, there is no independent evidence for either of these mechanisms, and no constraint on the timing. Despite much speculation, and several recent studies^39, 44–48^, the lack of detailed in situ constraints on the character, age and origin of the river deposits means that the specific role of river dynamics in the florescence and decline of Indus urbanism in this important region remains unresolved^25, 38, 39, 43, 49, 50^. Here we resolve these issues by characterising the nature of late Quaternary fluvial deposition, up to and including the time of Indus Civilisation urbanisation, near the drainage divide of the Sutlej and Yamuna rivers (Fig. 1). By determining the chronology and provenance of fluvial deposits, we focus on the effects of river avulsion on the onset and long-term stability of Indus urbanism in northwestern India.

## Results

### Remotely sensed imaging of the Ghaggar–Hakra palaeochannel

To map the large-scale modern and palaeo-drainage configuration of the region, we analysed the geomorphology using remotely sensed optical imagery and a Synthetic Aperture Radar (SAR)-derived digital elevation model (DEM) focussing in particular on the Ghaggar–Hakra palaeochannel.

**Fig. 2:**
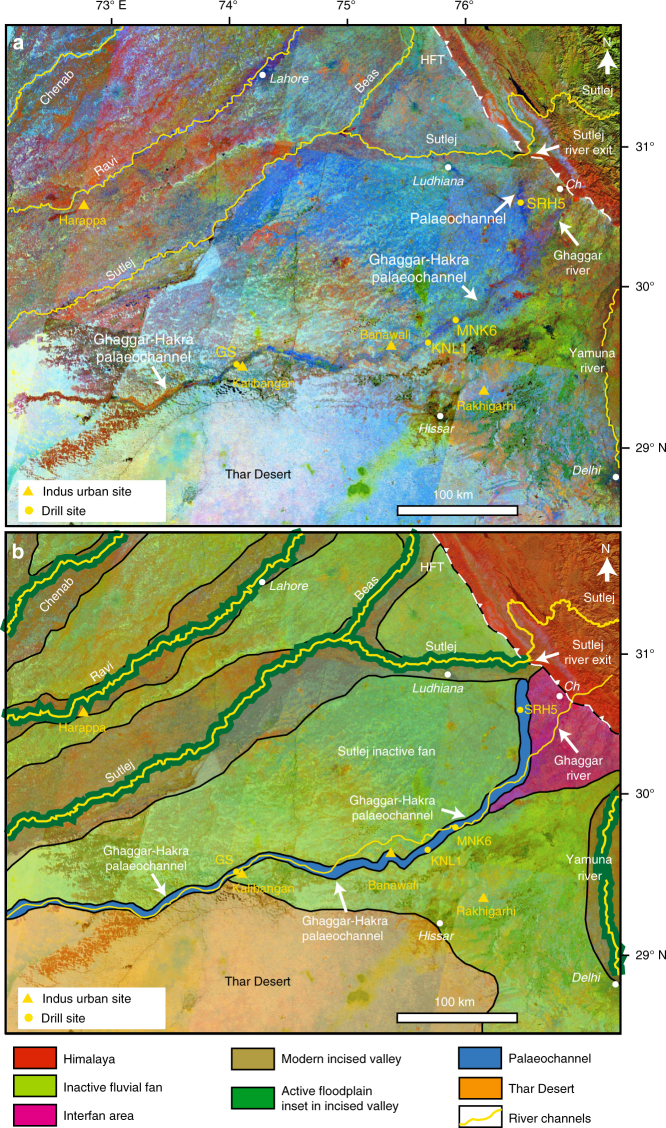
Trace of Ghaggar–Hakra palaeochannel on northwestern Indo–Gangetic plain. **a** Background shows Landsat 5 TM colour composite mosaic (bands 456). The Ghaggar–Hakra palaeochannel is visible as a sinuous, dark blue feature. Location of GS core sites adjacent to the Indus urban centre of Kalibangan, along with core sites at KNL1, MNK6, and SRH5, are also indicated. Location of key Indus urban settlements indicated by triangles. **b** Geomorphological map showing major alluvial landforms in the study region. *Ch*, Chandigarh; HFT Himalayan frontal thrust

We generated a new colour composite image mosaic from Landsat 5 Thematic Mapper (TM) scenes using spectral bands 456 (near infra-red, short-wave infra-red and thermal infra-red regions) displayed in the red, green and blue colour guns, respectively (Fig. 2; Supplementary Methods). The thermal infra-red (band 6) can be considered a proxy for surface temperature and shows the varying emittance of surface materials; during daytime imaging, damp conditions in the palaeochannel suppress both surface temperature and reflectivity, causing it to appear in a dark blue colour in Fig. 2. Areas outside the palaeochannel are characterised by drier conditions and therefore appear brighter and more reflective, while the Thar Desert is shown as white due to brightness in all bands (high reflectance in bands 4 and 5, and high emittance in band 6).

The large-scale geomorphology of the study area comprises two major fluvial fan depositional systems formed by the Sutlej and Yamuna rivers^51, 52^. Both of these rivers are currently deeply incised into older fan deposits, such that the fan surfaces are relict features that are disconnected from modern Himalayan river flow. We observe a distinct ~5–6 km wide sinuous feature (the dark blue feature in Fig. 2) on the Sutlej fan surface that extends ~400 km from the Sutlej River exit at the Himalayan mountain front to the Thar Desert. Our analysis suggests that the darker blue tone represents relatively cooler and less reflective surface materials, interpreted as sediments with higher moisture content. We interpret this damp and sinuous feature to represent the trace of the Ghaggar–Hakra palaeo-drainage system.

**Fig. 3:**
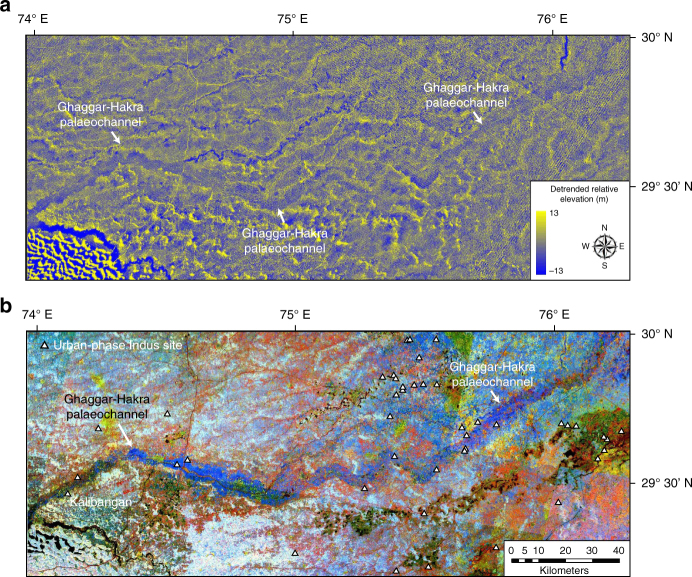
Topography of Ghaggar–Hakra palaeochannel. **a** Detrended relative elevation map of Sutlej–Yamuna drainage divide, derived from NASA Shuttle Radar Topography Mission (SRTM)^53^ 30 m DEM (2014 release) showing that Ghaggar–Hakra palaeochannel forms an incised valley. **b** Corresponding TM colour composite image (detail of Fig. 2) showing correspondence of Ghaggar–Hakra palaeochannel and incised valley. Locations of urban-phase Indus settlements along Ghaggar–Hakra palaeochannel are indicated

We investigated the topographic character of this palaeo-drainage system using the NASA Shuttle Radar Topography Mission^53^ (SRTMv3) DEM with a 1 arc-second or 30 m spatial resolution. Analysis of a relative elevation map derived from these data (Fig. 3) shows that the Ghaggar–Hakra palaeochannel observed in the colour composite image data corresponds to a topographic low in the landscape. This indicates that the palaeochannel forms an elongate and sinuous incised valley that is eroded several metres into the surrounding plains (Fig. 3).

### Sedimentary characteristics of the Ghaggar–Hakra palaeochannel

**Fig. 4:**
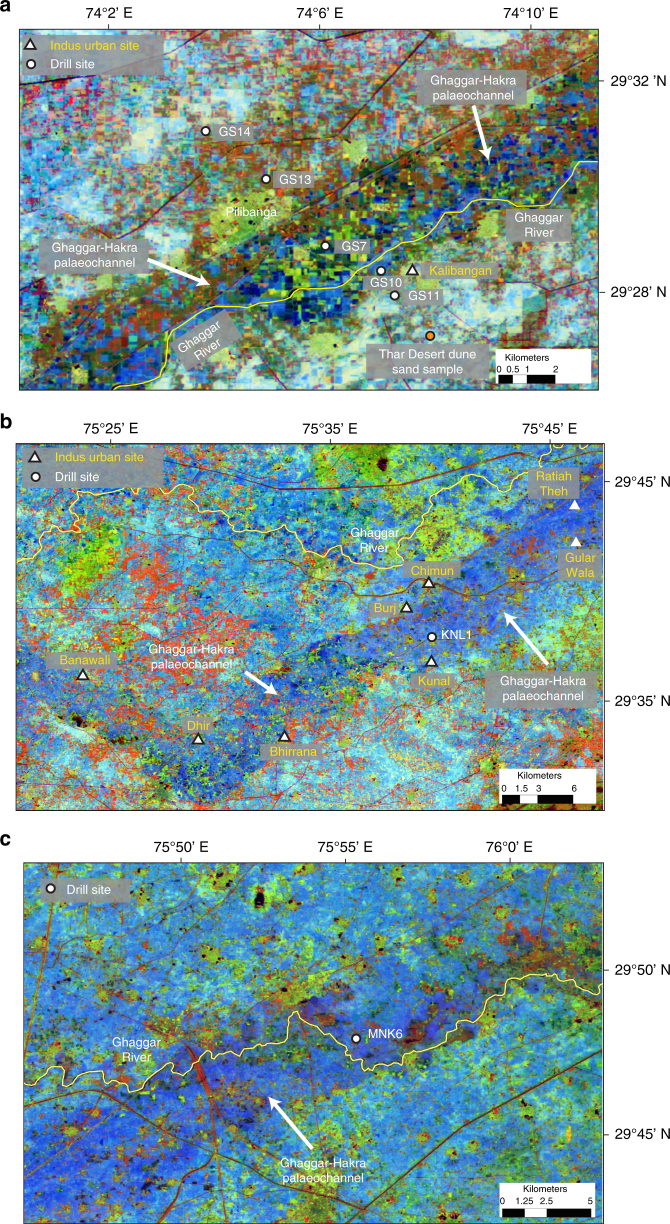
Locations of core sites along Ghaggar–Hakra palaeochannel. Background images are derived from Landsat 5 TM colour composite satellite mosaic shown in Fig. 2. White circles show locations of cores with relationship to Ghaggar–Hakra palaeochannel (dark blue tone). Course of modern ephemeral Ghaggar River is indicated in yellow. **a** Vicinity of Kalibangan Indus urban centre showing locations of cores GS14, GS13, GS7, GS10 and GS11. Location of Thar Desert modern dune sample also indicated. **b** Location of core KNL1. Urban-phase Indus archaeological sites in area are indicated by white triangles. **c** Location of core MNK6. Locations of all drill sites tabulated in Supplementary Table 1

To test the hypotheses that (1) the Ghaggar–Hakra palaeochannel hosted a major Himalayan river, and (2) that its abandonment coincided with Indus urban settlement decline, we drilled five cores perpendicular to the axis of the palaeochannel adjacent to the important Indus site of Kalibangan in Rajasthan^54, 55^ (Figs. 2, 4a) (29˚28'27'' N, 74˚7'51'' E). During its urban phase, Kalibangan comprised of two major walled mounds containing regular house plans, and a grid of streets^54^. The site is located topographically above the palaeochannel floor on the southern edge of the Ghaggar–Hakra palaeochannel^54^ (Fig. 4a). Analysis of the sedimentology of the Ghaggar–Hakra palaeochannel at this location enables us to understand the direct connection between river morphodynamics and Indus settlements.

**Fig. 5:**
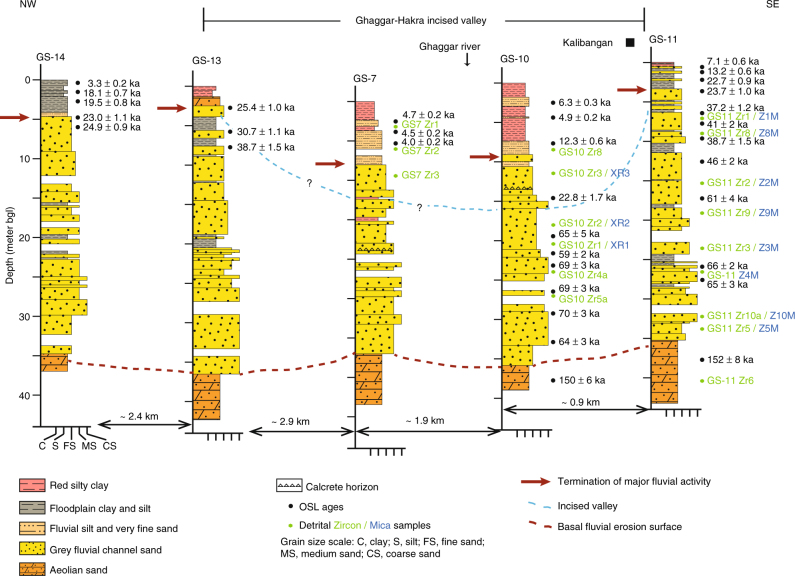
Stratigraphic panel showing core stratigraphy, sedimentology and K-feldspar OSL ages at GS core sites along transect across Ghaggar–Hakra palaeochannel near to Indus urban centre of Kalibangan. Note how in cores GS7 and GS10, sediments show young OSL ages that are inset into surrounding older strata, indicating that the sediments with young ages infill an incised valley. Additional evidence for this comes from the abrupt age disjunction observed in core GS10 at ~ 16 m depth, which defines the base of the incised valley. Sampling points for U-Pb detrital zircon and ^40^Ar/^39^Ar detrital muscovite analysis are also indicated. Stratigraphic sections are arranged in elevation. Dashed lines indicate basal fluvial erosion surface (red) and base of youngest incised valley (blue). Note variable horizontal scale. bgl below ground level

**Fig. 6:**
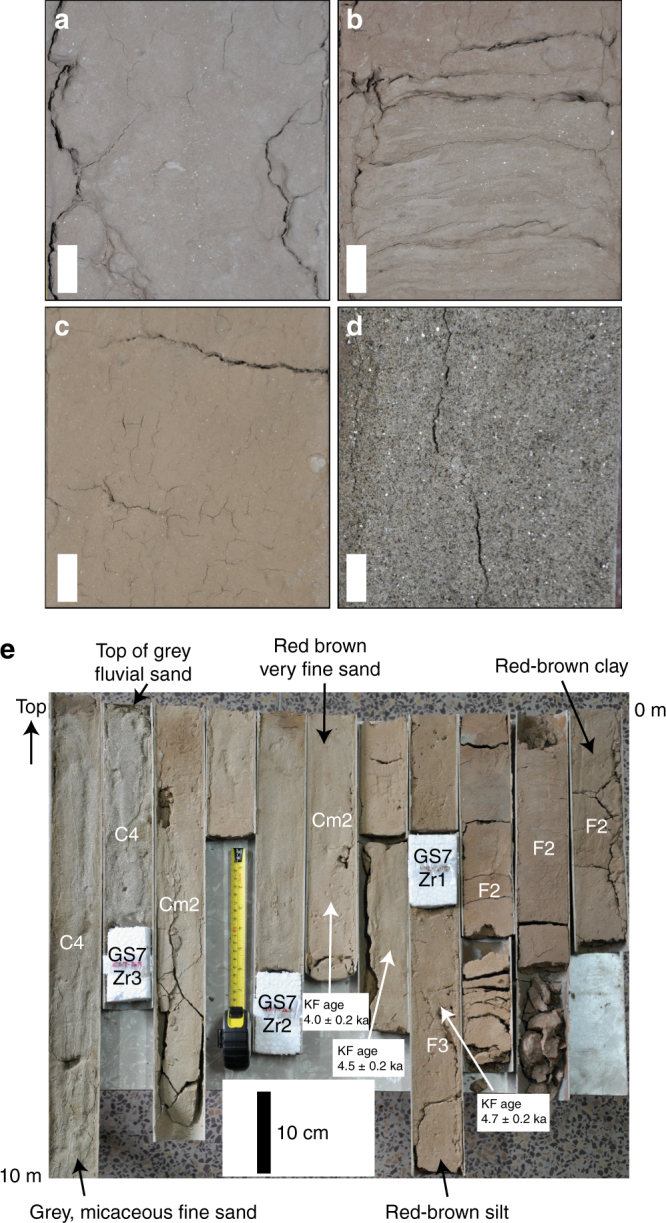
Characteristics of sediments in cores. **a**–**d** Detailed sedimentary features of core recovered from GS10 at Kalibangan. Scale bar is 1 cm in all images. **a** Silty clay at 2 m depth, **b** interlaminated silt and very fine sand at 4 m depth, **c** red-brown clayey silt at 6.5 m depth, **d** grey micaceous fine sand at 17 m depth. **e** Core recovered from GS7 at Kalibangan at a depth of 10 to 0 m, from the centre of incised valley. Facies abbreviations: F2, red-brown silty clay. F3, red-brown very fine sand. Cm2, yellow-brown very fine sand. C4, grey fine, micaceous sand. Details of facies are summarised in Supplementary Table 2. The base of the section comprises unconsolidated grey micaceous fluvial sands. Above these there is an abrupt transition into brown very fine sands and silts, and toward the top red-brown silty clays indicative of very low-energy depositional environments are present. Sampling points of detrital zircon samples GS7 Zr1-3 are indicated, together with K-feldspar OSL ages

The cores are dominated by a ~30 m thick fining-up succession of unconsolidated, dark grey, mica-rich, coarse- to fine-grained sand (Fig. 5). The sands have a distinctive ‘salt and pepper’ texture due to the abundance of dark heavy minerals (Fig. 6d). The grain size, poor to moderate sorting and abundance of angular grains in the sands indicate high-energy fluvial channel deposits. Thin beds of silt and clay interstratified within the sands and characterised by carbonate nodules, mottling and rhizoconcretions represent floodplain facies (Fig. 6a, c). Near the base of all cores, the grey sands sharply overlie light yellow-brown, well sorted, fine-grained sand that we interpret as aeolian dune deposits (Fig. 5 and Supplementary Fig. 1). These attest to an earlier phase of aeolian activity prior to fluvial incursion into the area. The grey sands, which comprise bedsets that are <5 m thick, likely represent fluvial bar- and channel-fill sediments that have become vertically stacked during multiple episodes of fluvial deposition. While the coring process does not preserve diagnostic sedimentary structures the textural character of the grey sands is typical of channel sands in modern Himalayan rivers in the region^56^. These channel deposits underlie and extend beyond the margins of the ~5 km wide surface trace of the Ghaggar–Hakra palaeochannel, as seen for example in cores GS13 and 14 (Fig. 5 and Supplementary Fig. 2) and inferred from geophysical data^44^. This demonstrates that a major river system once flowed across the Kalibangan area.

**Fig. 7:**
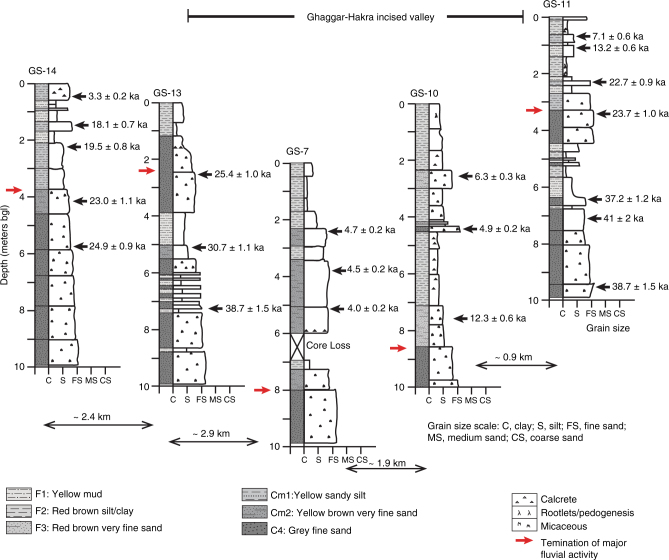
Stratigraphic panel showing detailed core sedimentology in upper part of GS section across Ghaggar–Hakra palaeochannel at Kalibangan. OSL ages are indicated. Red arrows demarcate top of grey sands indicating termination of major Himalayan fluvial activity in each section. Sedimentary sections are arranged in elevation. bgl, below ground level

Beneath the surface trace of the palaeochannel, in cores GS7 and GS10, the grey fluvial sands are overlain by an ~8 m-thick fining-up succession that shows upward transition from brown very fine sand and silt into reddish-brown silty clay (Figs. 5–7). These fine-grained deposits show evidence of weak pedogenesis, indicating relatively slow rates of deposition. The abrupt grain size change from the grey sand likely records a cessation of high-energy fluvial deposition and the onset of low-energy fluvial activity and suspension fall-out from standing, ponded water on floodplains. These very fine-grained sediments form a wedge-like unit that pinches out at the margins of the palaeochannel indicating that they were deposited in a palaeotopographic low.

### Chronology of palaeochannel fluvial sands

To establish if the grey fluvial sands were deposited by a major river adjacent to Kalibangan during the Indus urban phase, and to investigate whether the decline of Indus settlements along the palaeochannel was related to cessation of river flow, we determined the timing of fluvial deposition in our cores. Because rivers migrate laterally across floodplains, the timing of flow cessation varies in space and must be dated systematically across the entire channel belt. Thus, we dated the transition from grey sands to fine sediment across the Kalibangan transect.

We derived 52 optically stimulated luminescence (OSL) burial ages from seven cores using both the infra-red stimulated (IR_50_) signals from multi-grain K-feldspar aliquots, and blue/green stimulated signals from multi-grain and single-grain quartz aliquots (see Supplementary Methods: Optically stimulated luminescence dating and Supplementary Note 1) (Supplementary Tables 6–9). Single-grain quartz dose distribution analysis using standard rejection criteria and minimum age models gave improbably young ages with significant stratigraphic inversions and led to the implication that the degree of incomplete bleaching was a function of the subsequent burial time; this is physically unrealistic (Supplementary Note 2, Minimum single-grain ages). Alternatively, analysing the dose distributions using the Finite Mixture Model^57^ suggested unrealistic post-depositional mixing (Supplementary Note 3). The standard multi-grain IR_50_ fading-corrected feldspar ages were considered more likely. When additional rejection criteria (Fast Ratio^58^, and the D_0_ criterion^59^) (Supplementary Note 4) were applied to the quartz single-grain dose distributions, the resulting ages were consistent with the more precise multi-grain feldspar ages (Supplementary Note 5). This agreement supports the hypothesis that both signals were well bleached or reset at deposition^60, 61^ and thus the feldspar ages are used in further discussion (Supplementary Table 8).

For cores GS10 and GS11 (Fig. 5), we obtained OSL ages for the entire recovered succession. Aeolian sands at the base of both cores have ages of 150 ± 6 and 152 ± 8 ka, much older than the overlying fluvial sands. The grey fluvial sands in GS11 range from 66 ± 2 to 23.7 ± 1.0 ka, and in GS10 from 70 ± 3 to 23 ± 2 ka. These ages indicate that major fluvial activity in the region initiated during Marine Isotope Stage (MIS) 5/4 and persisted into MIS2. The dominance of channel sands in the GS section, with limited preservation of floodplain deposits suggests that the area formed a major fluvial channel belt that was re-occupied multiple times over ~40–50 ka. On the northwestern flank of the palaeochannel (cores GS14 and 13), the youngest coarser-grained fluvial sands are dated to 23.0 ± 1.1 ka and 25.4 ± 1.0 ka, respectively, and the oldest overlying fine-grained sediment to 19.5 ± 0.8 ka (Figs. 5, 7, Supplementary Fig. 2). On the southeastern flank, the youngest fluvial sands in core GS11 are dated to 23.7 ± 1.0 ka and the oldest overlying fine-grained sediment to 22.7 ± 0.9 ka (Figs. 5, 7).

In the centre of the transect, cores GS7 and GS10 penetrate the surface trace of the palaeochannel (Figs. 5, 7). Here, sediments with young OSL ages occur at greater depths than on the flanks of the palaeochannel (Figs. 5–7). Moreover, in GS10, we observe an abrupt age disjunction between two similar fluvial sandbodies at ~16 m depth, with coarse-grained sand dated to 23 ± 2 ka directly overlying deposits dated to 65 ± 5 ka. This indicates that the younger deposits are inset into older fluvial deposits across an erosional surface, and we interpret the younger deposits as partially filling an abandoned incised valley that is still partially preserved in the landscape. The mainly pre-Holocene ages exhibited in the uppermost strata on the northwestern and southeastern flanks of this incised valley (cores GS11 and 14) indicate that these topographically higher locations were largely disconnected from fluvial and overbank sedimentation during the Holocene.

Within the younger, incised valley fill, fine-grained sediments interpreted as low-energy fluvial and floodplain deposits range from 12.3 ± 0.6 to 4.0 ± 0.2 ka. In particular, the uppermost several metres of sediment are dominated by red silty clay (Fig. 6) that we interpret as deposition from suspension in standing water in the Ghaggar–Hakra floodplain, and that contrasts markedly from the sands that dominate the underlying succession. Taken together, these data imply that all fluvial activity indicative of a large river system terminated at this valley cross-section between ~23 and  ~ 12.3 ka.

### Regional analysis of the palaeochannel

**Fig. 8:**
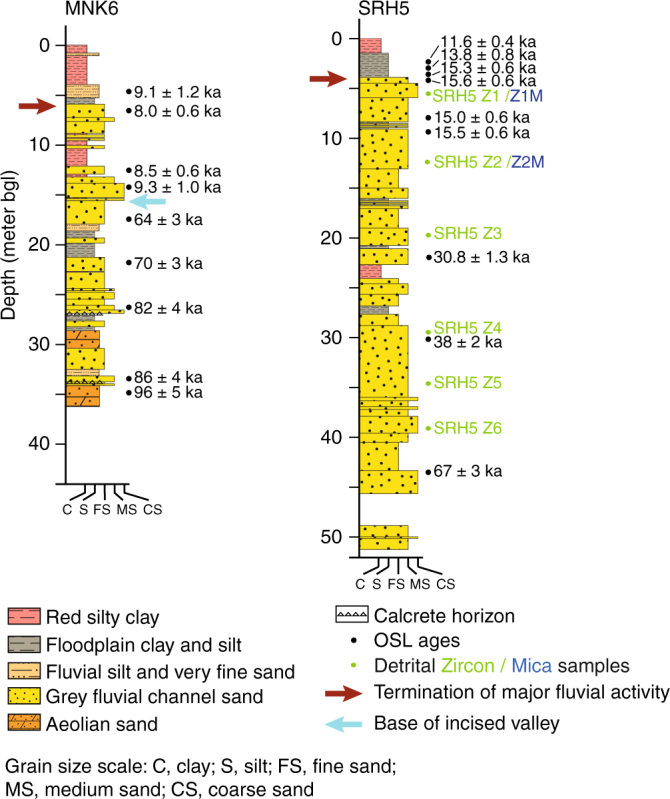
Core stratigraphy, sedimentology and OSL ages at MNK6 and SRH5 drill sites along Ghaggar–Hakra palaeochannel. Sampling points for U-Pb detrital zircon and ^40^Ar/^39^Ar detrital muscovite analysis are also indicated. Arrows indicate basal fluvial erosion surface (red) and base of incised valley (blue). Note major age disjunction at 16 m depth in core MNK6, indicating a major episode of fluvial incision and defining the base of the incised valley. bgl below ground level

In order to characterise the wider sedimentology and chronology of the Ghaggar–Hakra palaeochannel, we obtained three additional cores upstream of Kalibangan, two in the middle reach of the palaeochannel (sites KNL1 and MNK6), and one close to the Himalayan mountain front (site SRH5) (Figs. 2, 4). In all three cores, thick layers of grey, micaceous sands interpreted as fluvial deposits are overlain by several metres of silt and clay, indicative of the cessation of high-energy fluvial activity (Fig. 8 and Supplementary Figs. 4, 5). OSL ages on these cores enable comparison of the timing of fluvial activity with the sediments at Kalibangan. At MNK6, grey fluvial sands in the lower part of the core yield ages of 86 ± 4 to 64 ± 3 ka, and are sharply overlain by coarse sands at ~16 m depth that are dated at 9.3 ± 1.0 ka (Fig. 8). This age disjunction is evidence of significant erosion at this contact and confirms observations in core GS10 at Kalibangan that the younger deposits infill an incised valley. We note that the depth of this erosional boundary occurs at a similar depth in both cores GS10 and MNK6, suggesting that the depth of incision of the palaeovalley is similar. As at Kalibangan, grey fluvial sands at SRH5 and MNK6 are overlain by fine sand and silt interpreted as low-energy fluvial and floodplain deposits. At SRH5, the youngest grey fluvial sand is dated at 15.6 ± 0.6 ka with the overlying fine-grained unit exhibiting ages of 15.3 ± 0.6 to 11.6 ± 0.4 ka (Fig. 8; Supplementary Fig. 5). Thus, major river flow in the incised valley had ceased at this location by ~15 ka. However, at MNK6, the youngest fluvial sands show an age range of 9.3 ± 1.0 to 8.0 ± 0.6 ka, suggesting continued fluvial flow here up to ~ 8 ka. These data suggest that cessation of major fluvial flow along the entire length of the palaeovalley commenced at ~12–15 ka and was complete shortly after ~8 ka.

### Detrital zircon provenance of Ghaggar–Hakra palaeochannel

To constrain the source of the fluvial deposits, we determined the provenance of sand in the cores by using U-Pb detrital zircon age distributions to isotopically fingerprint erosional source regions. Because of marked contrasts of bedrock across the western Himalaya, U-Pb analysis of detrital zircons provides a valuable and widely used technique to discriminate source terrains for fluvial sediments in the Indo–Gangetic basin^62^. Age distributions from fluvial sands in core samples were compared with samples from modern rivers and published bedrock ages.

**Fig. 9:**
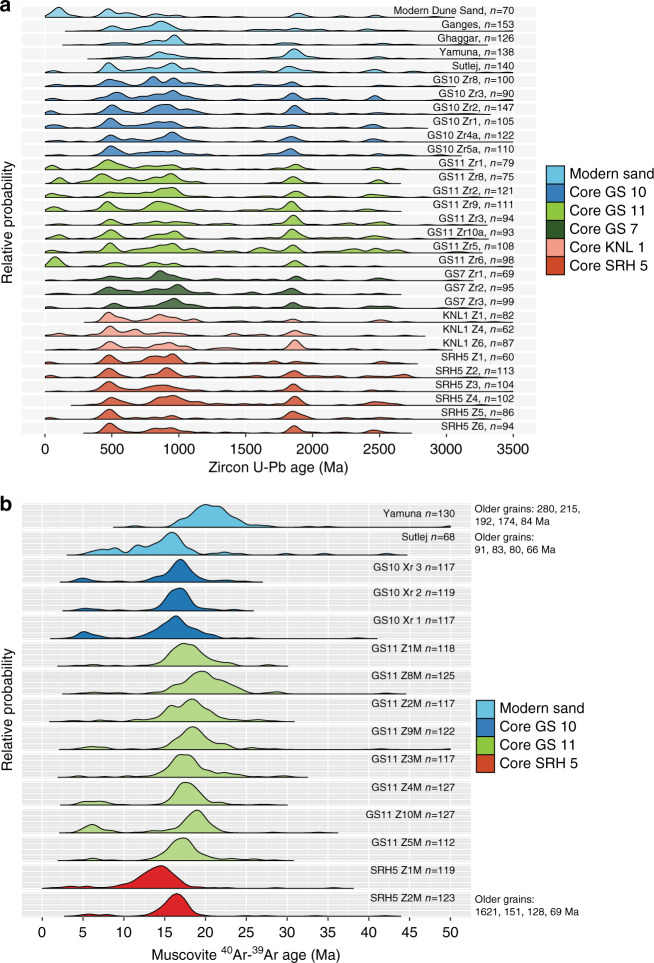
Age distributions of detrital zircon and muscovite grains for core, modern river, and aeolian dune sand samples. **a** U-Pb detrital zircon age distributions. Modern Sutlej sand shows a peak at ~480 Ma that is not prominent in Yamuna, Ghaggar and Ganges modern river samples. All fluvial sand samples from cores show distributions that match modern Sutlej river sand, thus identifying Sutlej catchment as the source of the fluvial sand underlying the Ghaggar–Hakra palaeochannel. A palaeo-Yamuna River cannot be ruled out as an additional contributor to GS and KNL1 sands, but cannot be a contributor to SRH5. Only GS11-Zr6 shows a different distribution; this sand is interpreted as an aeolian deposit below the fluvial succession and shows a good match to the modern Thar Desert dune sand. Sample locations shown in Figs. 2 and 4, and detailed in Supplementary Tables 1 and 3. Sample points in cores shown in Figs. 5, 8 and Supplementary Fig. 4, and described in Supplementary Table 4. **b** ^40^Ar/^39^Ar detrital muscovite age distributions. Two prominent peaks at ~15–20 Ma and ~4–6 Ma are present in the core samples. Both populations are present in the modern Sutlej sample, but the younger population is not present in the modern Yamuna sample, implying that the Sutlej catchment must be a contributor to fluvial sediments in the core. A palaeo-Yamuna River cannot be ruled out as an additional contributor to the GS fluvial sands but could not have contributed to the SRH5 fluvial sediments. Sample points in cores described in Supplementary Table 5

We conducted U-Pb isotopic analyses on 2508 detrital zircon grains from 26 samples from 5 cores, together with 630 grains from four modern rivers, and 70 grains from one modern dune sand (see Supplementary Methods: U-Pb dating; Supplementary Data 1). The modern river sands show markedly different age distributions with the Sutlej River in particular being characterised by a distinct peak at ~ 480 Ma. Fluvial sands from our cores show major peaks at ~800–1000 Ma and ~ 1600–1900 Ma (Fig. 9a), which is consistent with published bedrock ages from Higher Himalayan and Lesser Himalayan rocks, respectively^62–64^ (Supplementary Fig. 6). However, the majority of the fluvial sand samples from cores also show a prominent peak at ~480 Ma like that of the modern Sutlej river sample. We attribute this age peak to detrital zircons sourced from Palaeozoic granites exposed in the Sutlej river catchment^64, 65^. Notably, this peak is not dominant in the modern Yamuna, Ganges or Ghaggar river samples because the catchments of these rivers all lack prominent Palaeozoic granite bedrock^64^. This result strongly suggests that the Sutlej River was the main source of fluvial sediment to the Ghaggar–Hakra palaeochannel. The consistency of the zircon age distributions in fluvial sands taken from core samples traced from close to the Himalayan mountain front at SRH5 to Kalibangan, ~300 km downstream, strengthens the case that these sands were deposited by the same sediment routing system.

In addition to age peaks at ~480 Ma, ~800–1000 Ma and ~1600–1900 Ma, the GS cores collected at Kalibangan also show a young peak at <100 Ma that is not prominent in cores from further upstream or in modern river samples (Fig. 9a). This peak is also visible in the sample from the modern Thar Desert dune sand and in sample GS11 Zr-6, which is a buried aeolian sand at the base of core GS11. We interpret this young peak as originating from Thar Desert aeolian sand reworked into the fluvial system. Supporting evidence comes from the observation that this young peak is more prominent in samples from core GS11, located close to the Thar Desert fringe, than in samples from cores GS10 and GS 7, which are located more centrally within the Ghaggar–Hakra palaeochannel (Fig. 4a). This young (<100 Ma) grain population is inferred to be derived by aeolian reworking of Indus plain sediments, which were transported by the northeastward winds blowing across the Thar Desert^66, 67^. The young peak cannot be explained as input from the Sutlej or Yamuna rivers, as apart from Miocene leucogranites, there are no sources of <100 Ma zircons east of Ladakh/Khohistan/Trans-Himalaya in Himalayan bedrock. It is plausible that some of the ~20 Ma zircon grains could be derived from Cenozoic leucogranites exposed in the Higher Himalaya in the Sutlej catchment^68^.

### Detrital mica provenance of Ghaggar–Hakra palaeochannel

To isolate the effects of recycled zircons derived from eroded Himalayan foreland basin deposits, we also obtained ^40^Ar/^39^Ar ages on detrital muscovite grains to provide additional constraints on the provenance of the Ghaggar–Hakra palaeochannel. The ^40^Ar/^39^Ar ages record cooling of grains in the source region through the 350 ˚C isotherm and are controlled by exhumation rates^69^. Because the western Himalaya is characterised by marked across-strike variation in exhumation rates^70, 71^, detrital muscovite ages have the potential to fingerprint distinct bedrock source regions^72^.

We present 1560 single-grain muscovite ^40^Ar/^39^Ar ages from a total of 13 core samples, together with 198 ^40^Ar/^39^Ar ages from two modern river samples (Fig. 9b) (see Supplementary Methods: ^40^Ar/^39^Ar dating; Supplementary Data 2). We observe a prominent population of ~15–20 Ma grain ages, and a subsidiary peak of ~4–6 Ma ages. Notably, grains older than ~30 Ma are relatively rare. Very young ages (~4–6 Ma) are derived from bedrock units undergoing recent rapid exhumation, which is consistent with very young bedrock cooling ages from the Lesser Himalayan crystalline rocks in the Sutlej catchment^70, 71^. We deduce that the modern Ghaggar River, which erodes only Sub-Himalayan Miocene-Pliocene foreland basin deposits, cannot be a significant contributor to the fluvial deposits, because the rarity of older grain ages in our core samples implies that muscovite grains are not recycled from foreland basin strata^73, 74^ (Supplementary Fig. 7). In summary, the prominent ~480 Ma detrital zircon age peak derived from Palaeozoic granites and the ~4–6 Ma detrital micas both identify the Sutlej catchment, the third-largest Himalayan river, as the major sediment source for the buried fluvial deposits (Fig. 9, Supplementary Fig. 6).

### Statistical analysis of detrital zircon and mica ages

To quantify the dissimilarity between the zircon and mica age distributions (KDE plots in Fig. 9), we used a standard statistical method known as multidimensional scaling (MDS). Supplementary Fig. 8 shows a three-way MDS map of the pattern of similarity or dissimilarity among the detrital zircon and detrital mica age distributions. The plot groups samples with similar age distributions, and separates samples with different distributions, using the Kolmogorov-Smirnov (KS) effect size as a dissimilarity measure^75^. Fluvial sands from cores at GS-10, GS-11 and SRH-5 bear closest similarity to the modern Sutlej River sand sample, and are unlike the modern Yamuna River sand sample. This result confirms our inference that the fluvial sands from the cores are deposits of a former course of the Sutlej River.

## Discussion

Our study explores the evolution of major rivers on the western Indo–Gangetic plains and their effect on the development of urban-scale settlements of the Bronze-age Indus Civilisation. The migration of rivers has long been considered important in understanding the distribution of settlements in early civilisations. Indeed, river diversion or avulsion has been widely assumed to lead to settlement abandonment in early civilisations^3, 4^, although inadequate chronologies of both fluvial deposits and archaeological sites has limited the integration of fluvial and archaeological records. Recent studies in the desert Nile have shown that alluvial dynamics were important in determining whether climate-modulated fluctuations in river flow represented opportunities or hazards for Bronze-age farming communities^76^. It is clear that societal response to environmental change is not as straightforward as postulated in many studies. In the case of the Indus Civilisation it has been widely assumed that ancient urban-scale settlements developed adjacent to large rivers, which served as water sources. While this is demonstrably true for parts of the Indus geographical sphere^19, 21^, this assumption has led to the belief that the largest concentration of urban-scale Indus settlements, located on the drainage divide between the Yamuna and Sutlej rivers in northwestern India and in Cholistan, Pakistan, were contemporaneous with a Himalayan-sourced river that flowed along the trace of the Ghaggar–Hakra palaeochannel. Extension of this argument led to the supposition that diversion or drying up of this major river triggered the decline and abandonment of these urban sites from ~4.0–3.9 ka B.P.^14^. These ideas have dominated the discourse on environmental dynamics and Indus societal response during Indus times^50^.

**Fig. 10:**
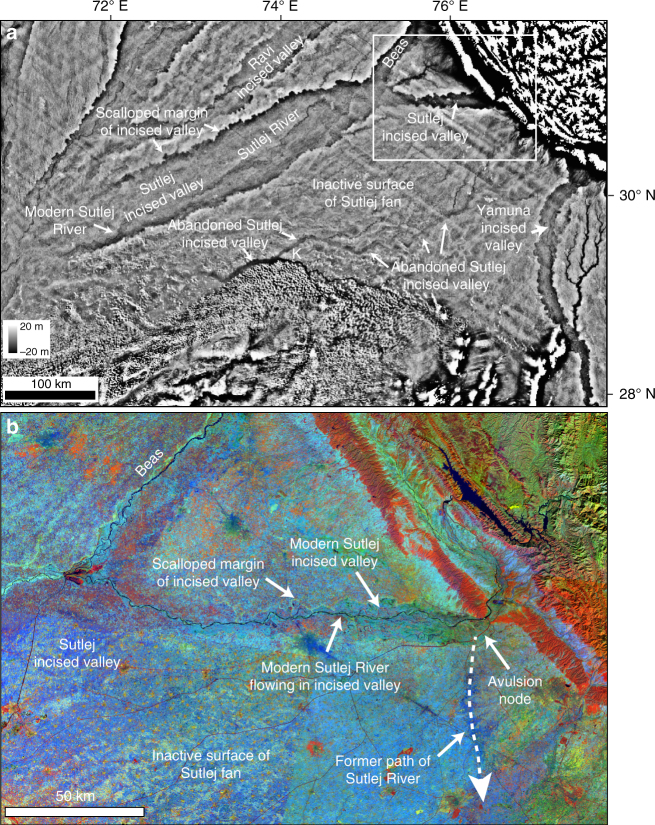
Topography of Sutlej–Yamuna plains showing modern Himalayan rivers occupy incised valleys. **a** Detrended relative elevation map, derived from SRTM 30 m DEM (2014 release), showing courses of the modern Sutlej, Beas and Yamuna rivers confined to regionally extensive incised valleys eroded into alluvial deposits of the Indo–Gangetic basin. Confinement prevents the rivers from readily avulsing across older fluvial fan surfaces. White box indicates area of detailed image in **b**. **b** Detail from Landsat 5 TM colour composite mosaic in Fig. 2 showing modern Sutlej incised valley near its outlet at Himalayan mountain front. Inferred palaeo-Sutlej course that joins Ghaggar–Hakra palaeochannel, a former Sutlej incised valley, is indicated, as is the likely river avulsion node

Our OSL-derived chronologies firmly establish that a major Himalayan river was not contemporaneous with Indus settlements in the Ghaggar–Hakra region and did not sustain the Indus Civilisation in this region. This finding resolves a question that has been debated for well over a hundred years. Our analysis shows that the Ghaggar–Hakra palaeochannel is a former course of the Himalayan Sutlej River that formed and occupied an incised valley from at least ~23 ka (Fig. 10a). Initial abandonment of this incised valley by the Sutlej River commenced after ~15 ka, with complete avulsion to its present course shortly after ~8 ka. This involved a lateral shift of the Sutlej River by up to 150 km, with the avulsion node located close to the Sutlej exit at the Himalayan front (Fig. 10). While we cannot identify the root cause of this avulsion, its timing after ~8 ka corresponds with the onset of a long phase of decline in the strength of the Indian Summer Monsoon (ISM)^77, 78^ that may indicate a possible climatic control on river reorganisation. However, it is important to point out that avulsion is an autogenic mechanism and need not mark a response to an external event.

Our study sheds new light on the role of river dynamics on early urbanisation. We find that the locus for the abundant Indus Civilisation urban settlements along the Ghaggar–Hakra palaeochannel was the relict, underfilled topography of a recently abandoned valley of the Himalayan Sutlej River rather than an active Himalayan river. We suggest that this abandoned incised valley was an ideal site for urban development because of its relative stability compared to Himalayan river channel belts that regularly experience devastating floods and lateral channel migration. It is also worth noting that many large Himalayan rivers are typically characterised by high avulsion frequencies, with rivers commonly revisiting past courses. For example, the Kosi River in the eastern Ganges basin shows an average avulsion frequency of 24 years^79^. However, in the western Ganges basin, rivers such as the Sutlej and the Yamuna flow in incised valleys that are deeply entrenched in abandoned alluvial plains (Fig. 10)^52, 80, 81^, and form regionally extensive sediment routing corridors. We suggest that confinement to incised valleys reduced the propensity for these rivers to frequently re-route. Since complete avulsion of the Sutlej River to its present course shortly after ~8 ka, the Sutlej has remained trapped in an incised valley and has not revisited its former Ghaggar–Hakra course. This has provided environmental stability within the Ghaggar–Hakra palaeovalley, a factor that may have helped to enable the long-term development of Indus urban settlements.

Following avulsion of the palaeo-Sutlej to its present course, the relict incised valley became partially infilled by very fine-grained sediments that we interpret as deposition from ephemeral monsoon-fed rivers derived from the Himalayan foothills, likely the equivalent of the modern Ghaggar River and its tributaries. Similar, very fine-grained infill was also documented by Saini et al.^45, 46^ along a section of the Ghaggar–Hakra palaeochannel. Thus, despite the diversion of the Sutlej, some fluvial flow and deposition of fine sediment continued in the topographic low formed by the relict valley. Our OSL dates from the upper part of the incised valley fill (core GS10) show that up to 6 m of fine-grained fluvial sediment were deposited from ~12.5 to ~5–6 ka, with only ~2 m of red clays above this section. The higher rate of deposition in the early Holocene corresponds to the interval of strengthened Holocene ISM from 10–7 ka^78^. The decrease in fluvial sedimentation after ~5 ka is likely due to the decrease in monsoon intensity documented after ~ 6 ka^78^. The fining-up character of the Holocene succession in our cores with very fine-grained sands and silts showing upward transition to silty clay suggests a progressive decrease in fluvial competence and decline in fluvial activity, which mirrors trends seen in the regional climate records of ISM weakening^78, 82, 83^.

The persistence of fine-grained fluvial sedimentation in the Ghaggar–Hakra incised valley during the mid-Holocene demonstrates that Indus urban settlements in the region were likely sustained by monsoon-fed fluvial activity. However, the Indus urban settlements were occupied at a time of strongly-reduced fluvial activity compared with the Himalayan-fed river system before ~15–9 ka or the moderate activity in the early Holocene. It thus seems improbable that Indus settlements flourished due to ‘perennial’ monsoon-fed river flow as proposed by Giosan et al.^39^. Likewise, our results show clearly that avulsion of the Himalayan-fed Sutlej, and decline in monsoon-fed fluvial activity within the Ghaggar–Hakra palaeochannel, predate both the establishment and decline of Indus urban settlements in the region, ruling out a causal link. Giosan et al.^39^suggested that decline in monsoonal rivers due to weakening of the ISM was responsible for this transformation of the Indus urban system. While independent climate records provide strong evidence for widespread weakening of the ISM across large parts of India at ~ ~ 4.2–4.0 ka^83^, and our cores indicate a marked decrease in sedimentation rate after ~5 ka, current fluvial chronologies lack the resolution necessary to draw robust conclusions regarding the influence of climate-modulated river activity on the decline of the Indus urban system. Future development of high-resolution chronologies for late Holocene fluvial records in this region may permit testing of climatic influence on river flow and its possible relationship to decline of Indus urban settlements.

A significant unresolved issue is that not all urban settlements in the region are necessarily co-located with the Ghaggar–Hakra palaeochannel^84^. The largest Indus site in the region, Rakhigarhi, widely considered to be of the scale of an Indus city^14, 16, 85^, is situated at least 50 km from the Ghaggar–Hakra palaeochannel. Although its location has been linked to another abandoned river system, the Drishadvati^85^, in situ data are necessary to determine the existence and timing of such river activity before drawing inferences on how such sites were sustained.

In conclusion, our results firmly rule out the existence of a Himalayan-fed river that nourished Indus Civilisation settlements along the Ghaggar–Hakra palaeochannel. Instead, the relict Sutlej valley acted to focus monsoon-fed seasonal river flow as evidenced by very fine-grained sediments in the upper part of the valley-fill record. This and the potential to pond flood waters in the topographic depression^38^ formed by the valley likely offered favourable conditions that led Indus populations to preferentially settle along the incised palaeovalley. We find that river dynamics controlled the distribution of Indus sites in the region, but in the opposite sense to that usually assumed: it was the departure of the river, rather than its arrival, that triggered the growth of Indus urban settlements here. We posit that a stable abandoned valley, still able to serve as a water source but without the risk of devastating floods, is a viable alternative model for how rivers can nucleate the development of ancient urban settlements.

### Data availability

The data that support the findings of this study are included in this published article (and its Supplementary Information files) or are available from the corresponding author upon reasonable request.

## Electronic supplementary material

Supplementary Information

Description of Additional Supplementary Files

Supplementary Data 1

Supplementary Data 2
