# Supplementary material for: Counter-intuitive influence of Himalayan river morphodynamics on Indus Civilisation urban settlements
Source: Nat Commun. 2017 Nov 28;8:1617. doi: 10.1038/s41467-017-01643-9 (PMC5705636; doi:10.1038/s41467-017-01643-9)
Supplement: Supplementary file 1 — Supplementary Information [file 41467_2017_1643_MOESM1_ESM.pdf]

Supplementary Methods

Remote Sensing Methods

Prior work[1] used Landsat 4 Multi-Spectral Scanner (MSS) satellite images to identify the surface trace of a possible palaeochannel on the Ghaggar-Hakra plains. These data are however of low spatial resolution and the study did not provide an explanation as to why such a feature was observed on image data. We generated a higher-resolution (30 m) colour composite image mosaic from 12 individual Landsat 5 Thematic Mapper (TM) scenes, acquired in November and December 1998, and using spectral bands 456 (near infra-red, short-wave infra-red and thermal infra-red regions) displayed in the red, green and blue colour guns respectively (Fig. 2). These bands have been chosen for several reasons: firstly, the visible bands 1, 2 and 3 are badly affected by atmospheric scattering so that 'true-colour' and 'standard' false colour infra-red composite images appear washed out and dominated by haze. Secondly, the short-wave infra-red (SWIR) region is where most vegetation, rocks and soils have their maximum reflectance and most diagnostic spectral features. Thirdly, the thermal infra-red (TIR) band 6 contains valuable information about water content, soil moisture and thermal properties of earth materials. The patterns in the colour composite reveal relative brightness changes in these 3 spectral bands which are controlled by variations in surface material chemical composition, texture and moisture content. Image data have been stretched using the Balanced Contrast Enhancement Technique (BCET)[2] and enhanced using a Direct Decorrelation Stretch (DDS)[3] and then manually colour balanced between scenes.

To investigate the topography associated with the Ghaggar-Hakra palaeochannel observed in the imagery we analysed a subset of NASA's Shuttle Radar Topography Mission (SRTMv3)[4] digital elevation model (DEM) with a 1 arc-second or 30 m spatial resolution. The raw SRTM DEM is dominated by the effect of the regional slope between higher ground in the north-east and lower elevations in the south-west. To remove this regional trend and thereby enhance the local topographic variations, a detrended relative elevation image was produced by subtracting the average elevation (as calculated using a low-pass 71 x 71 convolution filter, i.e., a moving window approximately 2x2 km, and scaled for visualization by a factor equal to the square of the filter size) from the raw elevations (Fig. 3).

Coring and Sampling Methods

Because the flat terrain of the Ghaggar plains does not offer any surface exposures to establish the alluvial stratigraphy, we carried out drilling of the subsurface sediments. Drilling operations were conducted, to retrieve undisturbed cores from depths of up to 35-50 m, at strategically chosen drilling sites (Figs. 2 and 4; Supplementary Table 1) along the surface trace of the palaeochannel. Core drilling was performed by rotary drilling using a diamond core bit mounted on the core tube barrel installed on a calyx drilling rig and rotated using a electric power generator. Bentonite powder was used as drilling mud and works as a lubricant, provides hydrostatic pressure to prevent groundwater in the drilled sands from entering in to the hole, facilitates the continuous removal of drill cuttings out of the hole, and most importantly cools the drilling bit. Sediment cores were retrieved in PVC pipes (~ 63 mm diameter) inserted in the core tube. After a drilling run of about 1 to 1.5 meters,

depending on the lithology, the whole drilling assembly was taken out and the drill bit was removed to pull the PVC pipe out of the drilling core tube barrel. The PVC pipe containing the sediment core was capped at both ends and labeled. The drill cuttings in the drill core bit were used for preparing field logs. Complete sediment cores are stored in the Indian National Core Archive facility at the Indian Institute of Technology - Kanpur, Kanpur (UP), India.

For sediment analysis, drill core pipes were split into two halves in a dark room preserving one half of the pipe for sampling for optically stimulated luminescence dating while the other half was used for detailed sedimentological logging and sample collection for analysis for dating and provenance. Colour, grain size, texture, mineral assemblages, inclusions such as nodules, rootlets, shells, carbonaceous material, and features like color mottling were documented. A qualitative estimate of grain size was made by following the grade scale proposed by Wentworth (ref. 5). The modal grain size was estimated by means of visual comparison to a reference card[6]. Texture is a combination of relative grain size, compactness and presence of any structures like lamination or lenses, or any inclusions like nodules, rhizoconcretions, shells, burrows. The core sediments were classified into sedimentary facies that are summarised in Supplementary Table 2. Three major sedimentary facies associations are identified: channel sand, floodplain fine-grained sediments and aeolian sand. Channel facies were identified mainly on the basis of sand grade particle size, poor to moderate sorting, thickness of sedimentary succession, and upward fining. Floodplain facies comprise finer-grained sediments, mostly silt and mud, in varying proportions along with iron and/or calcrete nodules, mottling and rhizoconcretions. Aeolian facies comprise yellow-brown, fine-grained, well-sorted sands (Supplementary Figure 1).

Optically Stimulated Luminescence Dating

Optically stimulated luminescence (OSL) dating determines the time elapsed since the last exposure of sediment grains to daylight (the burial age); this light exposure resets any prior latent OSL signal. During burial, mineral grains are exposed to environmental ionising radiation resulting in the creation of free charge. Some part of this free charge is trapped at defects and the net trapped charge is related to the total radiation exposure. In the case of quartz and feldspar, optical stimulation can release this trapped charge resulting in the emission of luminescence called OSL. The OSL signal can be calibrated in terms of absorbed dose (the equivalent dose, D_e; expressed in Gy). The burial age is then determined by dividing the D_e by the independently measured environmental dose rate (expressed in Gy ka^{-1}). We dated 52 samples extracted from seven individual sediment cores using the infra-red stimulated luminescence (IRSL) signal at 50°C (henceforth IR_{50}) from multi-grain K-feldspar aliquots (2 mm) as well as the blue/green OSL signals from multi-grain (8 mm) and single-grain quartz aliquots.

Fifty-three OSL samples (one modern sample from a Sutlej River sand bar and 52 samples from the seven cores) were prepared under subdued orange light conditions in the Nordic Laboratory for Luminescence Dating, DTU Risø Campus. The potentially light-exposed material from the ends of each selected core section was reserved for radionuclide

concentration measurements, and the inner portion used for water content and OSL measurements. For the majority of samples (coarse to medium sand) the grain size fraction 180–250 µm was first separated by wet sieving and then treated with HCl (10%), H_2O_2 (10%), and HF (10%). This quartz- and feldspar-rich mixture was then density separated using aqueous solutions of 'Fastfloat' to give quartz-rich (>2.62 g cm^{-3}) and K feldspar-rich (<2.58 g cm^{-3}) extracts. The quartz-rich extract was again etched in HF (40%) to minimize any residual feldspar contamination, before re-sieving to >180 µm. In those cases where the samples were dominated by fine sand, the 90-180 µm fraction was processed in the same manner, but with final sieving to >90 µm (see Supplementary Table 7). Finally, the luminescence purity of the resulting quartz extract was examined by testing the IRSL sensitivity (a measure of feldspar contamination). Thirteen of the 38 quartz samples were found to have a significant IRSL signal which could not be removed by repeated HF treatment; nine of these samples were then measured using pulsed optical stimulation (see below).

Automated Risø TL/OSL readers (TL-DA- 20,[7]) were used for all OSL measurements. Optical stimulation of multi-grain aliquots used infra-red (IR, 870±40 nm; ~130 mW cm^{-2}) and blue (470±30 nm; ~80 mW cm^{-2}) light emitting diodes (LEDs) and pulsed optical stimulation (POSL) used an integrated pulsing option to control the LEDs[8]. All POSL curves were measured using an on-time (pulse width) and off-time (time elapsed between two consecutive pulses) of 50 µs; this setting has been shown to give the best separation of quartz and feldspar OSL signals[9]. Data were only acquired during the off-time, collection began after a delay of 5 µs after the end of the light pulse, and stopped 0.7 µs before the beginning of the next pulse. OSL signals were detected using EMI 9635QA photomultipliers in combination with various detection filters. Feldspar IRSL signals were detected using a filter pack consisting of 2 mm Schott BG-39 in combination with 4 mm Corning 7-59. Quartz OSL signals were detected through 7.5 mm Hoya U-340 filters. *In situ* irradiations were made using calibrated $^{90}Sr/^{90}Y$ beta sources providing dose rates ranging between ~0.1 and ~0.3 Gy s^{-1} to quartz mounted on stainless steel discs.

Automated Risø readers fitted with single grain laser attachments[10] were used for single-grain OSL measurements. The stimulation light source was a 10 mW Nd:YVO_4 solid-state diode-pumped laser emitting at 532 nm, which was focused sequentially on a 10 by 10 array of grain holes spaced on a 600 µm square grid in special 10 mm diameter aluminium sample discs. The diameters of the single-grain holes were either 200 or 300 µm depending on the grain-size fraction measured. All single-grain discs were screened for contamination prior to measurement. Correcting the single grain data for beta source non-homogeneity using the approach described by Lapp et al. (ref: 11) did not result in significant changes to dose or dose distribution.

OSL measurements

The single-aliquot regenerative-dose (SAR) procedure[12] was used for equivalent dose determination. For K-rich feldspar measurements, optical stimulation for 200 s used IR LEDs with the sample held at 50˚C. The preheat and cutheat temperature were both 250˚C for 60 s

(ref. 13). A high-temperature IR bleach at 290˚C for 40 s was inserted in between the various SAR cycles to minimize recuperation. Initial $pIRIR_{290}$ measurements[14] were made using a preheat and cutheat of 320˚C for 60 s and a prior IR stimulation temperature of 50˚C. For quartz measurements, a double SAR procedure employing IR stimulation prior to blue or green stimulation (in both cases, sample at 125˚C, stimulation for 40 s) was used on all samples. A preheat temperature of 260˚C, 10 s and a cutheat temperature of 220˚C was used. A high-temperature blue bleach at 290˚C for 40 s was inserted in between each SAR cycle to minimize recuperation effects[15].

The IRSL signals from 2 mm diameter aliquots of K-feldspar were summed over the first 2 s of stimulation and corrected for background using the last 20 s of stimulation (i.e., late background subtraction). The OSL signals from multi-grain quartz aliquots (8 mm) were summed over the first 0.64 s of stimulation and corrected for background using the signal detected between 1.12 and 1.76 s of stimulation (i.e., early background subtraction EBG[16]). The effect of using EBG on quartz multi-grain aliquot data for these samples is shown in section 'Multi-grain quartz luminescence characteristics' below. The OSL signals from single grain measurements were collected during 1 s stimulations, and summed over the first 60 ms of stimulation and corrected for background using the last 0.25 s of stimulation.

The SAR dose response curves were based on a minimum of three regenerative dose points, a recuperation point and a recycling point. The doses reported were estimated by interpolation onto sensitivity-corrected dose-response curves fitted with a saturating exponential function

$$L'_D = L'_\infty\left(1 - e^{-D/D_0}\right) \quad (1)$$

where L'_D is the sensitivity-corrected OSL response to a given dose D (commonly denoted as the ratio L_x/T_x for laboratory dose and L_n/T_n for natural), L'_∞ is the saturation value and D_0 is a constant. Based on this interpolation model, the laboratory dose for which the response L'_D equals the corrected natural OSL signal L'_n is the so-called equivalent dose, D_e. The uncertainties on D_e estimated using individual aliquots or grains were assigned using "Analyst 4.00" (ref. 17); these consist of error propagation from counting statistics and curve fitting. For a significant number of quartz aliquots no bounded D_e estimates could be derived. Here we define a bounded dose estimate to be an estimate of D_e for which

$$D_e + s_D \quad (2)$$

was finite, where s_D is the standard error. Thus, an unbounded dose estimate is one where the natural sensitivity-corrected signal

$$L'_n + s_{L'_n} \geq L'_\infty \quad (3)$$

where $s_{L'_n}$ is the standard error of the natural sensitivity-corrected signal. Equivalent dose estimates were accepted if the uncertainty on the natural test dose response (i.e. the first test dose signal) is less than 20% and if the recycling ratio is within 15% of unity (multi-grain aliquots) or 2σ of unity (single grains). For single-grain analysis, dose estimates were also rejected if the IR depletion ratio[18] was not within 2 σ of unity, and if the recuperation dose was larger than 1 Gy.

Dosimetry

Radionuclide concentrations were estimated using high resolution gamma spectrometry, with ground and homogenized ~200 g samples mixed with wax to retain radon and cast in a reproducible geometry[19]. The resulting ^{238}U, ^{226}Ra, ^{232}Th and ^{40}K activity concentrations (Supplementary Table 7) were converted to dose rates following Guerin et al. (ref. 20). For K-feldspar extracts the internal beta dose rates from ^{40}K and ^{87}Rb were calculated assuming an effective potassium concentration of 12.5±0.5% and a rubidium concentration of 400±100 ppm (ref. 21).

Parent ^{238}U is not precisely measured in our gamma spectrometry facility; nevertheless, the arithmetic mean of the ^{226}Ra to ^{238}U activity ratio is 1.12±0.03 and the weighted mean is 0.94±0.02. We conclude that there is no evidence for significant secular disequilibrium in our samples in the first half of the uranium series. Nevertheless, for dosimetry calculations we conservatively assumed ^{234}U and ^{230}Th concentrations midway between the observed values of ^{238}U and ^{226}Ra with uncertainties equal to those of the ^{238}U analyses. We also assumed a 20±10% loss of ^{222}Rn compared to its parent ^{226}Ra. Supplementary Table 7 lists the resulting infinite matrix dry beta and gamma dose rates. Had we instead assumed uranium series equilibrium down to and including ^{226}Ra and based our uranium series activities only on ^{226}Ra analyses, then these dose rates would be, on average <1% different.

The total dose rates given in Supplementary Table 7 include a water content correction[22] based on the presumed lifetime average water content shown in column 6. These lifetime averages were based on laboratory-measured saturation water contents (with an associated uncertainty of ±4%) because most of our samples have lain below the water table for the entire burial period; even the shallowest samples will have been saturated for the majority of the burial period. Finally, a contribution from cosmic rays was added to the total dose rate[23].

K-Feldspar analysis

We initially studied the behavior of both the IRSL measured at 50˚C (IR_{50}) and that of the subsequent IRSL measured at 290˚C (i.e. $pIRIR_{290}$), both following a preheat at 320˚C for 60 s. It rapidly became clear that the $pIRIR_{290}$ signal was very poorly bleached, and thus we focus here on the IR_{50} signal.

K-feldspar luminescence characteristics The inset to Supplementary Figure 9a shows a typical natural IRSL stimulation curve measured at 50˚C (IR_{50}) for sample 101906, together with the regenerated stimulation curve resulting from a regeneration dose (in this case 143 Gy), both following a preheat at 250˚C for 60 s. The difference in decay shape between the two signals is presumably caused by anomalous fading in the natural signal[24]. Supplementary Figure 9a shows a typical dose response curve for the same sample for the IR_{50} signal. The dose response curve has been fitted using a single saturating exponential function and has a D_0 value of 245±7 Gy. The recuperation is small and the recycling value is 1.002±0.001. The natural dose is estimated to be 114±1 Gy. Supplementary Figure 9b is a histogram of the average apparent sensitivity change taking place in the SAR measurement for all natural K-rich feldspar samples. The average total sensitivity change is 0.81±0.03 (n=37). The inset to

Supplementary Figure 9b shows the sensitivity change for the aliquot shown in Supplementary Figure 9a. The average recycling ratio is 1.004±0.001 (n=37).

To examine the dependence of the equivalent dose from the IR_{50} signal on thermal treatment (e.g., preheat temperature) preheat plateau measurements using three aliquots at each temperature were measured for samples 111902 and -13 (see Supplementary Figure 9c). The preheat temperature (held for 60 s) was varied between 210 and 320˚C. The preheat temperature for the T_x cycles were kept the same as for the L_x cycles. As is often observed for the IR_{50} signal there is little dependence on the choice of preheat temperature in this temperature range and a preheat temperature of 250˚C, 60 s were chosen for all subsequent measurements.

All K-feldspar dose response curves were fitted with a single saturating exponential. The characteristic saturation values D_0 (average 284±7 Gy, n=320) are shown as a histogram in Supplementary Figure 9d.

K feldspar dose recovery Dose recovery measurements were done in two different ways. In the first set of experiments, 3 aliquots of 11 samples were bleached for 2 h in a daylight simulator (Hönle SOL 2) at a distance of 80 cm and subsequently given doses of ~ 45, 90 or 180 Gy. The dose recovery ratio shows no dependence of the size of the given dose and the ratio is 0.88±0.02 (n=11). In the other experiment, a dose of ~90 Gy was added on top of the natural signal for three aliquots from four samples (111902, -03, -12 and 19). To obtain the dose recovery ratio the natural dose for each sample (measured using fresh aliquots) was subtracted. The measured dose recovery ratio is independent of the natural dose (varying between ~12 and 50 Gy) and on average is 0.85±0.01 (n=4). This failure to accurately recover the laboratory given dose is commonly seen for the IR_{50} signal, and several authors[25,26] have failed to observe any correlation between dose recovery ratios and equivalent dose. Buylaert et al. (ref. 25) were able to compare two sets of IR_{50} measurements taken under different experimental conditions and giving different dose recovery ratios, and show that nevertheless both gave, on average, accurate estimates of equivalent dose (when compared with results expected from age control). As a result, we do not regard these minor deviations from the normal accepted range (0.9 to 1.1) as of significance.

K-feldspar fading rates Fading rates (g-values normalized to two days) have been measured on 3-6 aliquots from 37 samples using the approach described by Auclair et al. (ref. 13). The average g_{2days}-value for IR_{50} is 3.42±0.04% per decade (n=142), typical for the IR_{50} signal (Supplementary Figure 10a, b). The over-dispersion in the data is 13.0±1.0%. There is no correlation between D_e value and fading rate (Supplementary Figure 10c) as might be expected, i.e. variation in the laboratory fading rates determined on individual aliquots are not governed by variation in natural fading rates, but rather by some other cause. Thus, there is no justification for the use of aliquot-specific fading rates. This is not surprising given that these are multi-grain aliquots each containing more than 100 grains with all the resulting averaging effects. One is forced to conclude that accurate ages cannot be obtained by correcting the ages from individual aliquots by the individual fading rates. This is not a new observation; it has been reported before by Buylaert et al. (refs. 25, 27). Given that the CAM g-value is 3.41±0.04% per decade we are confident of our estimate of experimental uncertainty. As a result, we use the average estimate of the fading rate and standard deviation on the mean (i.e., standard) error to correct the average sample ages[5].

K-feldspar of modern analogue One of the possible limiting factors of the OSL technique is the potential problem of incomplete bleaching of the OSL signal prior to the last depositional event. The presence of incomplete bleaching will inevitably lead to age overestimation, but the question is whether this overestimation is significant. Several previously published bleaching experiments[28,29,25] have shown that the quartz OSL signal bleaches approximately one order of magnitude faster than the IR_{50} signal and approximately two orders of magnitude faster than the $pIRIR_{290}$ signal from K-feldspar. Thus, if incomplete bleaching is a significant problem it is expected to be much worse for feldspar than for quartz. One approach to estimating the likelihood of the presence of significant incomplete bleaching in a given environment is to measure appropriate modern analogues. Any modern analogue is by definition not yet a preserved sediment and so is very likely to undergo further daylight exposure before final preservation. In contrast, fossil material is only sampled after such final preservation, and so, in general, will have received more light exposure than the corresponding modern analogue. Thus, residual ages measured from modern analogues are likely to represent the worst-case scenario. Several studies[30-35] have reported IR_{50} doses from modern samples in the range from 10 mGy to 11 Gy and $pIRIR_{290}$ doses ranging between 1.6 and 54 Gy. In this study, we measured a modern analogue (sample 121915) which was taken from where the Sutlej river leaves the Siwalik foothills and enters the alluvial plain. This sample was measured using multi-grain aliquots of quartz and feldspar as well as single grains of quartz. Using the IR_{50} signal from the K-feldspar fraction we measured an average dose of 2.2±0.3 Gy (n=10), which corresponds to an age of ~0.7 ka. The smallest IR_{50} dose measured in this study is 9.4±0.6 Gy (n=8, sample 121906), which corresponds to a fading corrected age of 3.3±0.2 ka. We have not corrected any of the feldspar ages for the residual measured for the modern sample. Most probably these residual ages represent the worst case scenario, and it is expected that bleaching of buried samples is in general better than the modern analogues[32,36]. Even were we to assume that this modern analogue is of direct relevance to the residual doses in the older sediments, then it seems that incomplete bleaching of the IR_{50} signal is not likely to be of significance to the determination of the time when major flow ceased, because all fluvial samples are older than ~9 ka (for these samples, a 2 Gy residual signal would be ~10% of the D_e). The ages of some of the younger (fine-grained) samples might be somewhat overestimated, but these do not represent major river flow.

We also measured this modern sample using the $pIRIR_{290}$ protocol[14] and measured a dose of 48±20 Gy (n=6); corresponding to an age of 13±5 ka. This age off-set is comparable with the ages of the younger samples and indicates that the $pIRIR_{290}$ signal was not well-bleached at deposition; we do not consider this signal further.

Multi-grain Quartz analysis

Multi-grain quartz luminescence characteristics *Stimulation curve shape:* Supplementary Figure 9e shows a typical quartz dose response curve from sample 101906. The dose response curve has been fitted using a single saturating exponential function and has a D_0 value of 43±2 Gy. The recuperation is small and the recycling value is 0.97±0.02. The natural dose is estimated to be 82±11 Gy. The inset shows the natural OSL stimulation curve as well as the stimulation curve measured for the highest regeneration dose (in this case 197 Gy). These are very similar in shape, but in some aliquots the regenerated stimulation curve was observed to decay more slowly than the natural. The use of a background signal taken very close to the signal summation interval helps to make quartz OSL signals insensitive to such changes in shape by preferentially isolating the fast OSL component. To test whether or not this change in stimulation shape affects our large multi-grain aliquot quartz dose estimates

the equivalent doses were repeatedly calculated using an initial signal interval consisting of the first 0.32 s and a background 0.48 s wide. This background was moved progressively down the curve from immediately adjacent to the signal interval to the final 39.52 to 40 s for four samples (091901, 091906, 111901 and 111915). The average ratio of the dose based on the most stringent summation interval (first 0.32 s minus the subsequent 0.48 s) to that based on the standard summation interval (given in Supplementary Methods: OSL Measurements) is 1.03±0.03 (n=4). It is clear that the average equivalent dose is independent of the background interval chosen for these samples and that all results are consistent with those from our standard intervals. It is concluded that our equivalent dose estimates are insensitive to differences in decay shapes between the natural and regenerated signals.

Sensitivity change The SAR procedure is widely used in quartz OSL dating because of its ability to monitor and correct for sensitivity changes using the test dose correction. The sensitivity change as a function of SAR cycle number for the aliquot displayed in Supplementary Figure 9e is shown as an inset in Supplementary Figure 9f. This particular aliquot changes its sensitivity by a factor of ~22 and the average sensitivity change for all accepted aliquots for sample 101906 is 13.5±1.3 (n =15). In Supplementary Figure 9f the average total sensitivity change for all individual samples (n=37) is shown as a histogram. The average sensitivity change is about 5 times with the largest sensitivity change occurring for sample 111906 (27±2, n =21) and the smallest for 111905 (1.28±0.05, n =28). The re-measurement of a point on the dose response curve (i.e. a recycling point) gives information on the reproducibility of sensitivity-corrected luminescence signals; on average this recycling ratio was 1.008±0.009 (n=37) for all samples. Thus, although the sensitivity varies significantly during the measurement of the laboratory dose response curve, our SAR protocol is able to correct successfully for these changes.

Preheat plateau To investigate the dependence of the equivalent dose on our choice of preheat temperature we measured preheat plateaus for samples 101906, 111902 and 111903. The cutheat temperature (before the T_x cycle) was kept at 40˚C below the preheat temperature. Supplementary Figure 9g illustrates these measurements for sample 101906 and 111902. Each point is the average of at least four aliquots. It appears that our equivalent doses are insensitive to preheat temperatures ≤ 280˚C. Here we choose a standard preheat of 260˚C for 10 s and a cutheat of 220˚C.

Multi-grain quartz dose recovery To investigate whether a known dose can be recovered using the SAR protocol prior to any thermal treatment we undertook dose recovery experiments, where the aliquots were bleached twice for 100 s using blue light stimulation at room temperature, separated by a pause of 10 ks; this pause is intended to allow charge transferred to the 110˚C TL peak to decay to negligible levels. The aliquots were then given a beta dose in the range 15 to 115 Gy before subsequent measurement. Dose recovery measurements (see Supplementary Figure 12a) were undertaken for a total of 140 aliquots of 24 samples and we obtain an average dose recovery ratio of 1.03±0.02 (n=140), suggesting that a laboratory dose given before the first heating of these samples can be measured accurately using our protocol. This is of particular relevance to these samples given the very large sensitivity changes (up to 27 times) that are observed in both dose recovery and equivalent dose measurements.

Quartz modern analogue Murray et al. (ref. 32) summarise 67 average multi-grain quartz doses (including 40 from Jain et al. (ref. 36)) from young or modern quartz samples from fluvial and colluvial environments and find that the average residual dose is ~2 Gy. These authors further point out that their data should be considered as providing upper limits to likely residual doses at deposition, because they were taken from samples still undergoing

transport; such samples are expected to undergo further light exposure before final deposition and eventual preservation. Thus, in such environments, incomplete bleaching is likely to be of concern only for young samples (e.g., <10 ka).

The samples measured in this study are all (with the exception of samples 091905 and 111909) extracted from a major fluvial system, between ~50 km (SRH-5a) and ~300 km (GS cores) from the Himalayan mountain front onto a low gradient alluvial plain. Based on this geomorphological setting, we do not anticipate incomplete bleaching of quartz in this environment to be a significant problem. To test this hypothesis, we measured the quartz fraction of the modern analogue (sample 121915) taken from where the Sutlej river leaves the Siwalik foothills and enters the floodplain. For the multi-grain quartz measurements, a dose of 0.9±0.2 Gy (n=24) was measured. This corresponds to an age of ~ 400 years. We also measured 2,400 single grains and obtained a dose distribution with an average dose of 0.4±0.3 Gy (n=90) and a weighted dose of 0.06±0.01 Gy, corresponding to an age of ~25 years. Thus, if we assume that this modern analogue is appropriate to the buried samples being dated here, we can conclude that incomplete bleaching is not of significant concern for our samples.

Natural multi-grain quartz dose distributions Natural dose distributions were measured using large (8 mm) multi-grain quartz aliquots and the resulting dose distributions have relative standard deviations ranging between 16% (111911) and 59% (111913) with an average value of 33%. All dose response curves have been fitted using a single saturating exponential function and D_0 (exponential coefficient) values have been obtained for all aliquots. The average D_0 value for multi-grain quartz aliquots is 65±4 Gy (n=1197). A However, we were not able to derive bounded dose estimates for a significant fraction of the aliquots (~17% on average, using CW stimulation) which otherwise passed the rejection criteria outlined in section OSL Measurements. In the literature[37,38], it has been pointed out that if the L'_n value is close to the saturation value L'_∞, the uncertainties on the associated dose estimate D will be large and asymmetric[37,38] and Wintle and Murray (ref. 39) stated that it seems prudent to ensure that accepted dose estimates are smaller than $2\times D_0$. Although, the application of this $2\times D_0$ criterion may be appropriate when applied on a sample-by-sample basis, it will inevitably produce a bias to lower doses if applied on an aliquot-by-aliquot basis within a sample[40]. Because of this we have included all results which appear to intercept the laboratory dose response curve[41].

IR contamination As stated in section Sample Processing, 13 of the 38 quartz samples (including the modern sample 121915) showed a significant reduction in OSL signal after stimulation with IR, which is normally assumed to indicate feldspar contamination. All samples were measured using a double SAR protocol[42,43] where an IR bleach is inserted prior to blue stimulation to reduce the likely contribution from feldspar to the quartz dose. However, a blue-stimulated feldspar signal remained after IR stimulation and it has been shown that the simple IR bleach (here at 125°C, 100 s) in the double SAR protocol is not always sufficient to result in a pure quartz dose[30]. However, the luminescence lifetime of feldspar during blue light stimulation is significantly shorter than that of quartz[44-46] so it is possible to achieve an instrumental separation of the two signals using pulsed (POSL) stimulation. We applied a double SAR POSL protocol to 11 of the multi-grain quartz samples (9 samples with poor IR depletion ratios (111904, 05, 07, 19-24)).

In Supplementary Figure 13 the average CW D_0 values for individual samples are shown as a function of average IR depletion ratio (no IR depletion ratio has been measured for sample 111901, but the IR to blue ratio indicated significant IR contamination). If the IR signal arises from feldspar it is to be expected that samples with IR depletion ratios significantly smaller

than unity would have substantially higher D_0 values, because the blue stimulated signal from feldspar has much higher D_0 values than those from the quartz fast component. However, Supplementary Figure 13 does not show any apparent correlation between D_0 and IR depletion ratio. We can also compare the D_0 values obtained for CW and POSL stimulation. If the stimulation curves from these IR contaminated samples are significantly influenced by the presence of contaminating feldspar, one would expect that the D_0 values would be larger in CW mode than in POSL: the D_0 ratio between CW and POSL modes is 1.23±0.17 (n=9) indistinguishable from unity. Only two of the samples (111904 and -05) have D_0 ratios significantly greater than unity, e.g., 2.3±0.3 and 1.7±0.2, respectively. For these samples alone, it would appear that at high doses, where the quartz signal is in saturation, feldspar contamination may govern the shape of the dose response curve. For those samples where both methods were used, the ratio of the CW to POSL doses is 1.01±0.09 (n=8; data not shown; no POSL dose estimate could be derived from sample 111907, see below) indicating that any blue-stimulated feldspar contamination remaining after the IR bleach at 125°C is insufficient to affect the CW dose estimate significantly.

Although the average doses and D_0 values seems more or less unaffected by any feldspar contamination, the proportion of aliquots for which we are unable to provide bounded dose estimates increases significantly when we apply POSL stimulation. The average relative number of unbounded dose estimates for the nine samples in CW mode is 12±5% (n=9) and in POSL mode 45±11% (n=9), e.g. for sample 111907 we are not able to derive bounded dose estimates in CW for 33% of the measured aliquots (n=16/48) due to saturation whereas in POSL mode the corresponding number is 100% (n=7/7). This suggests that many of these samples are indeed at or close to saturation on the quartz growth curve, but that under CW stimulation the feldspar signal (unimportant at low doses) does allow the dose response curve to continue to increase at high doses; this results in an incorrect and apparently finite quartz dose estimate. We use the POSL data for the 9 IR contaminated samples. For the four remaining samples with significant IR signals (091904, 101902, 111901 and 111908) we did not pursue the use of the POSL mode since the average doses were indistinguishable between the POSL and CW-OSL mode. For these samples, we only used CW-OSL, and the relative number of aliquots rejected due to saturation is 18, 47, 0 and 33%, respectively.

Supplementary Figure 9h shows a histogram of all measured D_0 values (CW and POSL); the average D_0 is 59.9±1.0 Gy (n=1161), although the distribution is significantly positively skewed with the highest D_0 value being 426±168 Gy. These data are used below when discussing bounded and unbounded estimates of equivalent dose.

Dose saturation One of the major drawbacks of using quartz as a natural dosimeter for old samples is that a fast-component dominated signal saturates at relatively low doses, e.g. ~100 Gy (ref. 39). Of the 37 core samples measured using multi-grain quartz aliquots (8 mm), only 6 samples (101901, -03, 111901, -02, -10, -12) had no aliquots with the natural sensitivity corrected signal (L_n/T_n) appearing to be in saturation. These samples are all relatively young with OSL ages of ~ 6 ka, with the exception of sample 111902 which has an age of ~15 ka. The fact that many natural L_n/T_n values lie significantly above the saturation value for the majority of samples is of concern and implies that the measured natural signal did not grow up the same dose-response curve as is generated in the laboratory. This casts doubt on the accuracy of all equivalent dose estimates derived using multi-grain quartz aliquots; all aliquots presumably contain some grains for which the laboratory dose response curve does not describe the growth of the natural signal. Certainly, if a substantial number of aliquots appears to be in or above saturation it is likely that the derived average equivalent dose should be regarded as a minimum dose estimate. The relative number of aliquots rejected due to saturation is given in Supplementary Table 8 for each sample. Here, we have arbitrarily

chosen to regard all multi-grain average equivalent dose estimates as minimum dose estimates if the proportion of aliquots in saturation is larger than 15%. Thus, we regard the multi-grain quartz D_e estimates from 20 of the 37 samples as minimum dose estimates.

Single grain quartz analysis

To test if quartz and feldspar grains were well-bleached at deposition, quartz single-grain analysis was undertaken for 30 samples. Single grain OSL analysis is appropriate if significant incomplete bleaching is observed or anticipated[47,48]; it may also be justified for samples which have experienced post-depositional mixing[49], or as a means of rejecting grains with poor luminescence characteristics[50,51] – i.e., grains which do not accurately record the burial dose. We undertook single grain analysis using 30 (out of 37) core samples to investigate the unusually high rejection rate of multi-grain analyses due to saturation, and to further test whether incomplete bleaching may contribute to the scatter in inter- and intra-sample dose and age estimates.

Single grain luminescence characteristics All single grain analyses were measured using a standard preheat of 260˚C for 10 s and a cutheat of 220˚C as these thermal treatments were determined to be appropriate using multi-grain aliquots. The luminescence characteristics of single grains of quartz have consistently been reported to be highly variable between different grains both in terms of intrinsic brightness, stimulation curve and dose response curve shape for the same measurement conditions[47,52,53], and some selection criteria are required to identify grains suitable for further analysis.

Single grain rejection criteria We applied standard rejection criteria (see OSL Measurements) in a first attempt to obtain the most reproducible dose distributions. The effect of applying these rejection criteria to 30 single grain natural dose distributions on the normalized Central Age Model (CAM)[54] dose, the normalized over-dispersion and the normalized number of accepted grains are shown in Supplementary Figure 14a, b and c, respectively. In general, the application of these rejection criteria does not affect either the central dose or the observed scatter (quantified by the relative over-dispersion, OD) significantly; this conclusion applies both to natural dose distributions and to dose recovery dose distributions. Sample 111912 is the single exception to this statement. In this sample, although the central dose does not change significantly, the OD changes from 70±7% (s_{TN}<20%) to 32±6% (all rejection criteria). However, the ratio of ODs determined with and without rejection criteria for all other samples are not statistically different from unity. A similar conclusion was reached by Thomsen et al. (ref. 55), Guérin et al. (ref. 56) and Thomsen et al. (ref. 40) using samples of different origin. It would seem that the only significant effect of applying these rejection criteria is to significantly reduce the number of accepted dose estimates (by ~50%, on average). Nevertheless, for consistency with earlier published work, below we only consider the data which pass all the standard rejection criteria.

Intrinsic OSL brightness The natural OSL signals from 103,300 individual grains were measured from 30 samples. Of these grains, 3.6% gave both an acceptable test dose response (i.e. s_{TN}<20%) and a natural sensitivity-corrected signal which intercepted the laboratory regenerated dose response curve (i.e. gave bounded dose estimates). After application of the remaining single grain rejection criteria, 1.9±0.4% (n=30 samples) of the measured grains were accepted into the respective natural dose distributions. Supplementary Figure 15 shows the cumulative light sum[57] for all grains from one sample from each of the six cores; 80% of the light sum is derived from 5 to 20% of the grains and ~40% of the grains do not contribute significantly to the light sum. In single grain analysis it is common for the sensitivity of

individual grains to vary by over four orders of magnitude[58] but for these samples the average variation in sensitivity is only about two orders of magnitude with the majority of grains being only weakly luminescent. The inset to Supplementary Figure 15 shows the net OSL signal per Gy from the natural test dose signal for six representative samples for the grains accepted according to the selection criteria given in section OSL Measurements; the grains have been ranked from brightest to dimmest[58]. For comparison, the results from a Namibian fluvial sample (031304 (ref. 59)) and a Danish coastal deposit[55] are also given. The quartz samples from our study are in comparison significantly dimmer than published material. For the samples in this study the median of the natural test dose OSL response per grain is 1.78±0.09 Gy^{-1} (summed over the initial 60 ms of stimulation; 1966 grains, 30 samples). For comparison, the corresponding numbers for the fluvial sample (031304) from Namibia is 94 Gy^{-1} (525 grains) and for the recent Danish coastal deposit is 78 Gy^{-1} (358 grains).

Single grain OSL stimulation curve shape Multi-grain quartz measurements have shown that the quartz OSL signal consists of different components with differing photoionisation cross sections and thermal stabilities[51,60]. It is generally accepted that only samples dominated by the fast component are likely to give accurate estimates of burial dose[61,62]. As a consequence, many laboratories routinely use an early background subtraction (EBG) for multi-grain quartz measurements to minimize the contribution from slower decaying and potentially less bleached components. Supplementary Figure 16 shows five representative, normalized OSL stimulation curves for the samples measured in this study; there is a large variability in curve shape. Although such large variability is commonly observed, only a few single grain OSL studies have examined the impact of including dose estimates derived from grains with varying decay rates on the final dose distribution. Ballerini et al. (ref. 63) concluded that single grain dose estimates can be over-estimated if doses derived from slowly decaying OSL signals are accepted into the dose distributions and suggested that EBG ought to be applied. In a single grain quartz study by Reimann et al. (ref. 64) it was observed that using EBG instead of LBG (late background subtraction) apparently reduced the relative OD (from 34±2% to 30±3%) because the use of EBG preferentially leads to the rejection of low sensitivity quartz grains, but that this had no significant effect on the average burial dose estimate. This is in agreement with the observation by Thomsen et al. (ref. 55) who reported that doses from weakly luminescent single grains were significantly more over-dispersed than those from bright grains. The EBG approach was used by a number of authors[65-67], but all concluded that for their samples no significant benefits could be observed. Madsen and Murray (ref. 68) and Durcan and Duller (ref. 69) suggested using the fast ratio F_R to classify the decay shapes of individual stimulation curves. F_R is given by $(F\text{-}BG)/(M\text{-}BG)$, where F is the initial summation, M is a subsequent summation and BG is the late background. The fast ratio F_R was applied by Duller (ref. 70) in his single grain dose recovery experiments and he observed a significant improvement in laboratory relative OD although the dose recovery ratios remained underestimated for given doses approximately > 100 Gy. In contrast, Feathers (ref. 71) observed both an improved dose recovery and a reduction in OD in laboratory and natural dose distributions. Thomsen et al. (ref. 55) also applied the F_R approach to their single grain dose recovery data but only observed a small reduction in relative OD for some of their samples. Jacobs et al. (ref. 65) applied the F_R approach to their natural samples and found no significant effects on either burial dose or relative OD.

We calculated the F_R for six of our single grain dose recovery data sets (e.g., 111904, -10, 12, 15, 101903 and -06, see Single grain dose recovery experiments) using the summation of the initial 30 ms of stimulation as F, the summation of the subsequent 30 ms as M and the last 150 ms as BG. The F_R values for the five normalized OSL stimulation curves shown in Supplementary Figure 16a are given in parenthesis in the legend. The distributions of F_R

values are all positively skewed with an average mode of 1.8±0.1. To assess if the rate of decay influences the estimated dose and the relative OD, we have calculated the average (arithmetic) dose, the CAM dose and the relative OD for grains with F_R>3 and for all grains irrespective of F_R value, respectively, and then derived the ratio of these values calculated from the two data sets. (On average only ~20% of the accepted grains have F_R>3.) The weighted mean of this ratio is 1.04±0.04 (n=6) for the average dose and 1.07±0.04 (n=6) for the CAM dose. Thus, we do not see a significant change in estimated dose by applying the F_R criterion. For the ratio of the relative OD, the weighted mean is 0.92±0.11 (n=5), and all ratios (except for 111915) are consistent with unity within 2 standard errors. Thus, rejecting grains based on the F_R values does not seem to introduce a significant change in relative OD. However, for sample 111915, which has a relative OD of 26±4% (n=72) when all grains irrespective of F_R value is included in the calculation, we observed that by rejecting grains with a F_R value less than 3, the data set becomes under-dispersed. This does lend some support to the observation made by Duller (ref. 70), although it should be noted that the F_R<3 data set only contains 9 dose points, and so the apparent reduction in the unexplained scatter may be simply a statistical fluctuation.

We also calculated the F_R for 25 of our natural data sets. The weighted ratios for the arithmetic mean and the CAM are 1.10±0.04 (n=25, 96% consistent with unity within 2 standard errors) and 1.08±0.04 (n=25, 88% consistent with unity within 2 standard errors), respectively. Thus, if there is an effect on the estimated dose it is small. The weighted ratio for the relative OD is 0.79±0.07 (n=25, 96% consistent with unity within 2 standard errors), which would imply that we observe a significant reduction in OD. However, this ratio includes sample 111921, which has a relative OD of 55±11% (n=34), when all grains are included and a relative OD of 15±9% (n=11), if we only include those grains with a F_R >3. If we were to eliminate this sample from the data set then the weighted ratio is 0.87±0.07 (n=24), which is again consistent with unity. Thus, it would appear that the decay rate of individual single grains does not seem to significantly affect either the estimated dose or the relative OD.

Single-grain OSL dose response curves In the literature it has been reported[53,70] that the shapes of the dose-response curves of individual grains are highly variable, e.g., some dose response curves saturate at high doses (high D_0 values) whereas others saturate at low doses (low D_0 values). In this study, the D_0 values of the dose response curves (all fitted with a saturating exponential function) also vary widely from grain to grain. Supplementary Figure 16b shows three measured dose response curves for three of the grains whose stimulation curves are shown in Supplementary Figure 16a. In contrast to Jacobs et al. (ref. 65), who stated that there was some support for the suggestion that grains with low D_0 values also had the slowest rates of OSL decay (e.g., low F_R ratios), we see no correlation between D_0 and F_R for these samples. Supplementary Figure 17b is a frequency histogram of the D_0 values known to better than 30% of the accepted grains from sample 111909. The average weighted D_0 value for this sample is 112±5 (n=229) Gy with a median value of 109 Gy. The average weighted D_0 for all samples is 94±6 Gy (n=30) which is significantly higher than that observed for multi-grain aliquots (60±1 Gy). For all samples, we were unable to derive bounded dose estimates for ~30% (880) of the grains which otherwise pass all standard rejection criteria. The number of grains rejected due to saturation varies between 4% (111912) and 63% (091905); the fraction of grains in saturation generally increases with depth (age), as might be expected. It is interesting to note that the D_0 values of grains rejected due to saturation are, on average, only 52±3% (n=30) of the D_0 values of the accepted grains. A similar observation was made by Thomsen et al. (ref. 55) in their dose recovery experiments. However, the average L_n/T_n ratio of the grains rejected due to saturation is

2.3±0.1 (n=30) times those of the accepted grains. Thus, grains rejected because of saturation have both smaller D_0 values and larger L_n/T_n ratios than accepted grains. Both these characteristics will individually lead to unbounded dose estimates. Again, we observe no correlation between D_0 value for these saturated grains and F_R value (data not shown).

Single grain sensitivity change As found using multi-grain aliquots, the accepted single grains also showed significant sensitivity changes throughout the SAR measurement sequence. The average sensitivity change for all samples is 4.4±0.6 (n=30 samples). As in the multi-grain data, the largest average sensitivity change (11.5±1.3, n=59 grains) is observed for sample 111906 and the smallest (1.2±0.2, n=18 grains) for sample 111905. The CAM average of the sensitivity change observed in multi-grain measurements to that observed using single grain measurements is 1.33±0.15 (n=30 samples); in addition, there is no indication, in terms of sensitivity change, that rejected single grains behave differently from accepted grains. In contrast to Gliganic et al. (ref. 72), we see no correlation between the size of the sensitivity change and the rate of OSL decay (data not shown).

Single grain thermal transfer and recuperation To assess the importance of thermal transfer and recuperation for the chosen thermal treatment (preheat of 260˚C, 10 s and cutheat of 220˚C) portions of samples 091903, -04 and 111904 were bleached for 1 hour at a distance of 80 cm in a daylight simulator (Hönle SOL2). A total of 4200 grains were measured and 39 grains were accepted into the dose distribution (data not shown), to give an average dose of 2.2±1.0 Gy (n=39), a CAM dose (assuming a normal distribution) of 0.6±0.2 Gy, and an over-dispersion of 0.6±0.3 Gy. Thus, thermal transfer and recuperation do not appear to be a significant problem for these samples for the chosen thermal treatment.

Single grain dose recovery experiments Single grain dose recovery experiments, similar to those carried out for multi-grain aliquots, were undertaken for 10 samples (091903, -06, 101903, -06, 111903, -04, -10, -12, -15 and -16). A total of 21,800 individual grains were measured and 680 (~3%) of these passed the standard rejection criteria. The ten individual dose recovery ratios are shown in Supplementary Figure 12b. The average dose recovery ratio (measured/given) is 0.98±0.04 (n=10 samples). The inset to Supplementary Figure 12b shows the dose recovery ratio as a function of given dose using the standard rejection criteria (filled symbols). We appear to be able to measure accurately a dose given in the laboratory prior to any thermal treatment. However, the variability observed in the individual dose distributions is significantly larger than expected from the assigned uncertainties (see section OSL Measurements), i.e. the average relative OD is 32±2% (n=10), significantly larger than the typical OD (~10%) reported in beta dose recovery experiments. In a series of dose recovery experiments performed on a recent coastal deposit from Denmark, Thomsen et al. (ref. 55) showed that the observed OD in their single grain dose distributions depends on the intrinsic brightness; i.e. the OD calculated from bright grains is smaller than that calculated from dim grains. Given the low inherent OSL sensitivity of the quartz samples investigated here, it is possible that at least a part of this additional variability arises from this phenomenon. Regardless of the origins of this OD, these dose recovery results indicate that although we can, on average, measure a given dose accurately, the precision is low; we cannot know an individual dose estimate to better than ~30%, regardless of the calculated uncertainty associated with the individual dose. Thus, we have added (in quadrature) a 30% uncertainty to that derived from counting statistics and fitting (unless otherwise stated) to all individual dose estimates before any subsequent statistical analyses.

Single grain natural dose distributions Natural quartz single grain dose distributions were measured from 30 of the 37 samples discussed here. Individual D_e estimates were obtained

for between 1200 and 7200 grains from each of the 30 samples. The number of accepted individual dose estimate ranges between 18 and 229. Relevant statistical information is provided in Supplementary Table 9. Supplementary Figure 18 shows representative single grain dose distributions for four of the samples (111910, 091906, 111915 and 111918). On the left the natural test dose signal is shown as a function of the dose estimated from individual grains and the insets show the same data as a simple frequency histogram. Radial plots of the same data are shown on the right, with individual data points assigned a minimum uncertainty of 30% (in addition to the uncertainty arising from counting statistics and curve fitting errors). (Note that Supplementary Figure 17a shows an additional dose distribution for sample 111909; there the natural test dose signal is expressed as a function of dose.) All dose distributions include a wide range of individual dose estimates, e.g. the dose distribution for sample 111918 contains individual dose estimates ranging between -0.2±1.0 Gy and 208±65 Gy and for sample 111909 they range between -8±10 Gy and 560±100 Gy. These two samples were extracted from the cores at 5.85 m (GS-14) and 36.89 m (GS-10), respectively and we confidently reject the possibility that the zero dose estimates for these samples arise from young intruding grains. Others[73] have also reported the existence of such zero dose grains in old samples; they were also unable to explain these apparently very young grains in terms of their depositional context. However, as we have screened the individual grain holes for contamination (stuck grains) before use, we are confident that any zero dose grains that we record have not arisen from contaminated discs. It would therefore be incorrect to simply reject these grains, because of the inherent risk of incorrectly biasing the dose distributions towards higher doses. In the absence of a credible extrinsic explanation for these doses (e.g. post-depositional mixing) we must accept that such extreme outliers simply indicate the degree of scatter in our measurements – that is, they reflect the intrinsic OD. Nine of the 30 single grain dose distributions contain such negative dose estimates (111912, 101901, 111903, -09, 101903, -04, 091901, -04 and 111918).

All natural single grain quartz distributions have been obtained using a double SAR protocol with an IR diode bleach at 125˚C for 40 s prior to green laser stimulation. In addition to this, only grains with an acceptable IR depletion ratio have been accepted. Thus, we are confident that our dose distributions are not significantly affected by feldspar contamination. Nevertheless, the existence of grains with very low doses extracted from old samples might indicate signal instability; although the fast component OSL signal from multi-grain quartz aliquots has been shown to be stable (>10^8 years), little information concerning stability on a grain by grain basis is available. To investigate the short-term stability, we undertook simple fading measurement on 2,400 grains from sample 111909, for which we previously had determined the natural dose. The fading measurement sequence consisted of three prompt L_x/T_x measurements separated by two delays of 3 days duration. In this experiment, the ratio of the averaged delayed L_x/T_x to the average prompt L_x/T_x measurements was 0.97±0.02 (n=83) and we did not observe any correlation between this ratio and the measured dose. This confirms that at least this quartz sample is unlikely to suffer from anomalous fading, or other forms of short-term instability.

The relative ODs of the natural dose distributions (calculated with individual uncertainties assigned on the basis of counting statistics and curve fitting errors alone) range between 32±6% (n=73, sample 111912) and 105±19% (n=61, sample 101904), with an unweighted average of 64±3% (weighted 61±3% with an OD of 20±6%) for all samples. Only two samples (111910 and -12 from GS-7) have ODs consistent with the OD reported for the single grain beta dose recovery experiments. There appears to be no correlation between OD and dose.

U-Pb geochronologic analysis of detrital zircons

Zircon U-Pb analyses were performed by LA-ICPMS using a New Wave Nd:YAG 213 nm laser ablation system, coupled to an Agilent 7500 quadrupole mass spectrometer. Real-time data were processed using GLITTER v4.4 data reduction software (www.glitter-gemoc.com). Repeated measurements of the zircon Plesovice standard (TIMS reference age 337.13 ± 0.37 Ma (ref. 74) and NIST 612 silicate glass[75] were used to correct for instrumental mass bias and depth-dependent inter-element fractionation of Pb, Th and U. Reported ages are based on the 206/207 ratio for grains older than 1.1 Ga and the 206/238 ratio for younger zircons. Grains with discordance >10% were omitted. Examination of discordant grain ratios showed that in most cases the discordance was due to mixing of multiple growth stages, rather than simple lead loss. Details of locations of modern river and dune samples are given in Supplementary Table 3, and locations of cores together with sample depth and lithology are given in Supplementary Tables 1 and 4. U-Pb isotope data for detrital zircons are presented in Supplementary Database 1.

^{40}Ar/^{39}Ar geochronologic analysis of detrital mica

The location and details of sample depths in cores are provided in Supplementary Tables 1, 3, 5. Samples were subjected to magnetic separation. Mica was handpicked under a binocular microscope to ensure a pure separate. Approximately 150 crystals per sample were picked. After rinsing in de-ionised water and methanol, the grains were parceled into Cu packets and positioned within an Al holder for irradiation with International ^{40}Ar/^{39}Ar Age Standard Fish Canyon sanidine (FCs, 28.201 ± 0.023 Ma (ref. 76)). Samples were irradiated in two batches. The first batch of samples were irradiated for 10 hours in the Cd-lined facility at the McMaster Nuclear Reactor, Ontario, Canada. The second set were irradiated in the Cd-lined CLICIT facility TRIGA reactor at Oregon State University, USA. Monitors were analyzed by total fusion with a focused CO_2 laser.

Single grains (200-400 µm) of unknowns (i.e., muscovite) were loaded into a Cu planchette in an ultra-high vacuum laser cell with a doubly pumped ZnSe window. Using a CO_2 laser, the muscovite crystals were fused. All gas fractions were subjected to 180 s of purification with two SAES GP50 getters (one at room temperature the other at 450˚C) and a cold finger maintained at -95.5˚C using a mixture of dry ice ($CO_{2[s]}$) and acetone. Argon isotope ratios (i.e., ion beam intensities) were measured using a MAP 215-50 mass spectrometer in peak jumping mode. The mass spectrometer has a measured sensitivity of 1.13×10^{-13} moles volt^{-1}. The extraction and cleanup line was automated, as were the mass spectrometer peak jumping routines and data acquisition. Blanks (full extraction line and mass spectrometer) were measured after every two analyses of unknowns. The average blank ± standard deviation of n = 812 from the entire blank run sequence was used to correct raw isotope measurements of unknowns (^{40}Ar 1.02×10^{-15} moles, ^{39}Ar 3.10×10^{-17} moles, ^{38}Ar 1.90×10^{-17} moles, ^{37}Ar 7.85×10^{-17} moles, ^{36}Ar 1.38×10^{-17} moles). Mass discrimination was monitored by analysis of air pipette aliquots after every ten sample analyses (n = 174, 7.21×10^{-14} moles ^{40}Ar, ^{40}Ar/^{36}Ar = 289.61 ± 0.57). The Ar isotope data were corrected for backgrounds, mass discrimination, and reactor-produced nuclides and processed using standard data reduction

protocols. The decay constants of Steiger and Jager (ref. 77) and atmospheric argon ratios of Nier (ref. 78) were employed.

The BGC software *MassSpec* was used for data regression. Raw Ar isotope data are presented in Supplementary Database 2.

Supplementary Note 1: KF and Multi-grain Quartz ages

K-feldspar ages Supplementary Table 8 shows all fading corrected ages obtained using the IR_{50} signal; these are shown on stratigraphic panels in Figures 5, 7 and 8, and shown as a function of depth in Supplementary Figure 11a for cores GS-10 and GS-11. The relative uncertainty (1 standard error) on these ages ranges between 4 and 13% with an average of 5.0±0.3% (n=52). The feldspar ages are generally in stratigraphic order with the exception of two young samples (111901/101901 and 111912). In GS-10 we appear to have a small but significant age inversion at the very top of the core; sample 111901 at ~2.3 m gives an age of 6.3±0.3 ka whereas sample 101901 at ~4.3 m gives an age of 4.9±0.2 ka. In core GS-7 (where only 3 fine-grained samples were measured) sample 111912 at ~5.1 m has an age of 4.0±0.2 ka and may be younger than the two overlying samples 4.5±0.2 ka and 4.7±0.2 ka). In core SRH-5 we have measured nine samples taken between ~2.4 and 44 m. The ages are all in stratigraphic order down to the bottom of the core which is dated to 67±3 ka.

In core GS-10 a total of 11 samples have been measured. These samples were taken between ~2.3 and 37 m (see Supplementary Figure 11a). Apart from the age inversion observed in the very top of the sequence (see above) the feldspar ages increase monotonically until a depth of ~14 m, below which the ages are all consistent with an average of 66±2 ka (n=6) until beyond 32 m. The deepest sample (111909) at 37 m, of aeolian origin, gives an age of 150±6 ka. The age of the uppermost medium sand sample in GS-10 is 23±2 ka.

In core GS-11 we have measured a total of 12 samples taken from depths between ~ 0.5 m and 37 m (see Supplementary Figure 11b). The ages increase monotonically with depth and as in core GS-10 there is evidence for a rapid sedimentation period at ~65 ka (mean age of samples between ~17 and ~32 m is 64±2 ka; n=3). The deepest sample (091905) taken at ~37 m from an aeolian deposit is 152±8 ka, which is consistent with the age obtained from the aeolian horizon in core GS-10. The age of the uppermost medium sand sample is 23.7±1.0 ka.

We have measured three samples from core GS-13 and five samples from core GS-14. All the ages are in stratigraphic order and the ages of the uppermost medium sand samples are 25.4±1.0 ka and 23.0±1.1 ka, respectively. The average age for all available uppermost fine/medium sand samples from all cores on the GS-transect is 23.7±1.0 ka (n=4).

Nine samples were measured for core MNK-6. A remarkable hiatus of ~55 ka is detected between 14.5 and 17.5 m (Figure 3, Supplementary Table 8). Below this break, the ages increase with depth and range from ~65 ka at 17.5 m to ~95 ka at the bottom of the core (35.2 m). The top of the core is characterised by a period of rapid deposition at 8.7±0.3 ka (n=4).

Quartz multi-grain ages Supplementary Table 8 summarizes the quartz multi-grain equivalent doses and resulting ages for all 38 samples. The uncertainty (1 se) on individual ages range between 5 and 25% with an average uncertainty of 9.0±0.6% (n=37). As discussed above, the majority of these samples give minimum ages; only 17 of the 37 core samples are regarded as giving bounded ages. In core SRH-5A, only sample 111923 (22.6±1.5 ka, n = 37) is regarded as a bounded age. However, all ages from a depth of ~3 m are consistent with an average age of 20.6±0.7 ka (n=5). The youngest age (not to be confused with modeled single-grain minimum ages) of the uppermost fine- to medium-grained sand sample is >20 ka.

In the very fine-grained section of core GS-7, all three samples have less than 3% of the aliquots in saturation and so all are bounded. As was seen in the feldspar data, the quartz multi-grain age for the deepest of these samples (111912, 4.9±0.3 ka) is significantly younger than the age of the overlying sample 111910 (6.0±0.4 ka); this suggests some mixing of well-

bleached material, or an error in dose rate, rather than incomplete bleaching (because a small degree of incomplete bleaching in feldspar – as seen here – should imply complete bleaching of quartz[10]).

In core GS-10, five samples give bounded age estimates (see Supplementary Figure 11c, closed symbols). At the top of the core, there is a small age inversion between samples 111901 (2.3 m; 8.0±0.6 ka) and 101901 (4.3 m; 6.3±0.3 ka). The same inversion is present in the feldspar data, and (as above) we deduce that this is unlikely to arise from incomplete bleaching. Sample 111901 showed a significant IR contamination, but unfortunately, we did not have sufficient quartz to re-measure this sample using POSL. Otherwise the ages are in stratigraphic order and the age of the uppermost coarse/medium sand sample is 20±2 ka.

In core GS-11 there are several stratigraphic inconsistencies for the ages obtained for samples taken from below 6 m (Supplementary Figure 11d). However, all of these ages are derived from samples with a significant number of unbounded dose estimates and we thus consider these to be minimum ages. The remaining five samples are in stratigraphic order. The age of the uppermost fine-medium-grained sand sample is 23±2 ka.

In core GS-13, two of the three multi-grain quartz ages are considered to be minimum ages, but they all appear to be in stratigraphic order. The minimum age of the uppermost fine-to medium-grained sand sample is >26 ka.

In core GS-14, the three samples are not in stratigraphic order, and the ages of the first two samples are both regarded as minimum ages. The age of the deepest sample is 24±2 ka and the minimum age of the uppermost fine-medium-grained sand sample is >19 ka.
Bearing in mind that two of the four ages for the uppermost fine-medium-grained sand samples from the GS-transect are considered to be minimum ages, the average age of these samples is 22.3±2.3 ka (n=4), completely consistent with the corresponding result from multi-grain feldspar, of 23.7±1.0 ka (n=4).

Supplementary Note 2: Single grain Quartz ages

Single grain quartz ages have been estimated using a range of different approaches. In Supplementary Table 9 we summarize average ages, both weighted (CAM) and unweighted (arithmetic) averages, minimum ages derived using three different minimum age models, the most prominent finite mixture model ages (FMM_{prom})[79] all derived from the data sets obtained using the standard rejection criteria (see section OSL Measurements).

In general, the rationale for carrying out single grain measurements is to enable the identification of minimum burial ages for samples expected to be incompletely bleached or to identify (and reject) grains likely to have undergone post-depositional transport. It has often also been stated that the single grain technique enables the OSL characteristics of each individual grain to be evaluated, so that only reliable dose estimates contribute to the age determination[50,73]. We suggested that a large fraction of the multi-grain quartz ages must be regarded as minimum age estimates because a significant fraction (>15%) of the individual aliquots were in saturation (see Supplementary Note 1). By undertaking single grain analysis, we might be able to come up with more accurate age estimates for all samples, because we are able to reject individual grains with L_n/T_n values in or above saturation.

Average single grain quartz ages For well-bleached samples that have not undergone significant post-depositional mixing, the best estimate of the burial age should be based on an average (usually weighted) dose estimate, even in the presence of a spatially variable dose rate[80]. As stated above, nine of the single grain dose distributions contain negative dose

estimates. The original CAM (i.e. CAM_{log}) assumes that natural dose distributions are log-normal and this prevents the application of this model to distributions containing non-positive dose estimates. For these nine samples, we calculate CAM (i.e. CAM_{UL} in the terminology of Arnold et al. (ref. 81)) ages assuming the dose distributions are normally distributed; this allows the inclusion of all values. However, we note that the assumption of normality as against log-normality has no significant effect on the estimated dose or OD. The ratio of CAM_{log} dose (calculated after the rejection of negative dose estimates) and the CAM_{UL} doses (including all accepted dose estimates) is 1.01±0.04 (n=9 samples) and the ratio between the corresponding ODs is 0.99±0.04 (n=9 samples). If we were to apply the $L_n>3\ \sigma_{BG}$ (refs. 73, 82) criterion, in an attempt to avoid the inclusion of aberrant "0 Gy" grains, then these ratios are 1.07±0.05 and 0.96±0.04, respectively. If we only include samples expected to be older than 10 ka (based on the KF ages for the same samples) then the ratios are 1.07±0.07 and 0.99±0.04 (n=6 samples). Thus, for the nine samples containing negative dose estimates, the CAM ages and ODs given in Supplementary Table 9 are, within uncertainties, insensitive to the underlying assumption of the shape of the dose distribution.

The CAM ages for the individual cores are broadly in stratigraphic agreement with each other. The three ages for SRH-5A are all consistent with an average age of 13.5±0.6 ka. The CAM age of the uppermost medium- coarse-grained sand sample (111922) is 14±2 ka (n=37).

Only two samples were measured for GS-7 (111910 and -12) and they give CAM ages of 6.1±0.4 ka (n=76) and 4.6±0.3 ka (n=73), respectively. This apparent age reversal is also seen in both the feldspar and the multi-grain quartz ages.

The CAM ages for GS-10 are all in stratigraphic order (with the exception of sample 111906), although the data becomes noisy below 15 m (see Supplementary Figure 11e). The CAM age from the uppermost medium-grained sand sample (111903) in this core is 16±2 ka (n=69).

In core GS-11, all ages are in stratigraphic order except for sample 091904, which appears to be significantly younger than the ages derived from the samples taken above and below (see Supplementary Figure 11f). The single grain dose distribution for this sample contains a significant number of grains consistent with zero, nine of which are negative. Thus, it is not surprising that the CAM age derived for this sample appears to be too young. Unfortunately, we have not been able to make single grain measurements for the uppermost medium sand sample in this core (101905) due to lack of sample material.

Three samples have been measured for GS-13 and GS-14, respectively. The single grain CAM ages are stratigraphically consistent and give ages for the uppermost fine- to medium-grained sand samples of 16±2.0 (n=24) and 20±2 (n=42) for GS-13 (111913) and GS-14 (111917), respectively.

The average CAM age for the uppermost fine- to medium-grained sand samples from the GS-transect is 17.3±1.4 ka (n=3).

In single grain measurements, there is often a large variability in the intrinsic luminescence sensitivity and consequently a large variability in the relative uncertainties assigned to each individual dose estimate. Thus, it seems prudent to weight each individual dose estimate with respect to its individual uncertainty as is done in the CAM. However, the large relative ODs observed for these samples, even in dose recovery experiments, raises doubts as to the validity of the weighting factors. Thus, we have also calculated average arithmetic single grain ages for the accepted grains. These ages follow the same stratigraphic order as the CAM ages but are on average 21±2% (n=30) older than the CAM ages and gives an average

arithmetic age of the uppermost fine- to medium-grained sand samples on the GS-transect of 20.9±1.8 ka (n=3).

Minimum single grain ages In our view, the main reason for undertaking single grain analysis is to establish whether incomplete bleaching is significantly affecting these samples. In the literature it has often been argued that well-bleached samples have a relative OD of 20% or less[58,83]; from this it is deduced that samples with a larger OD are likely to be affected by incomplete bleaching and thus that the burial age should be determined using minimum age models[83]. If the minimum uncertainty of 30% determined from the single grain dose recovery experiments is taken into account then all dose distributions (derived using standard rejection criteria) have relative ODs significantly larger than 20% with the exception of the two young samples measured from core GS-7 (samples 111910 and -12, which have relative ODs of 20±3% and 12±2%). Bailey and Arnold (ref. 84) suggested a decision tree making use of the relative OD, the skewness and the kurtosis of individual dose distributions to decide which model to apply to extract an accurate burial dose. Although this decision tree was developed for fluvial systems, it has since been applied to many different depositional environments including fluvial, glacio-fluvial, colluvial, alluvial, coastal and aeolian (e.g., Fuchs and Owen (ref. 85); Delong and Arnold (ref. 86); Fattahi et al. (ref. 87); Costas et al. (ref. 88); Stone et al. (ref. 89); Gaar and Preusser (ref. 90)) to determine whether minimum dose modelling is appropriate.

If we apply this decision tree to our fluvial samples then, a minimum age model (either MAM3, MAM4 or the lowest 5%) is chosen for all samples except 111912, 111913, and 111919 for which the CAM should be applied. Thus, from the literature it is to be expected that at least the majority of these samples are incompletely bleached and hence that minimum age models should be applied. Consequently, we have applied three different minimum age estimation methods: (i) the four parameter minimum age model (MAM4 (ref: 54)), (ii) the internal external consistency criterion (IEU) (refs. 59, 91), and (iii) the finite mixture model (FMM_{min}) (refs. 79, 92). Nine of the dose distributions contain negative dose estimates (111912, 101901, 111903, -09, 101903, -04, 091901, -04 and 111918) and for those distributions we have applied the original MAM script after doing a simple exponential transformation of the data[67]. Unfortunately, we were only able to derive reliable single-grain MAM4 estimates (i.e., good fits for all four parameters) for 19 of the 30 samples. We could not derive IEU minimum dose estimates for three of the samples (111902, 111917 and 111918) because of the presence of what appears to be low dose outliers. For the 17 samples where both MAM and IEU ages could be derived we obtain no significant differences between the two minimum dose estimation approaches; all dose estimates are consistent within two standard errors except sample 101901 which is consistent at three standard errors. Rodnight et al. (ref. 92) suggested that the FMM could be applied to identify the well-bleached component in incompletely bleached samples. They preferred the FMM to the MAM as they observed that the MAM was more sensitive to the presence of low dose outliers. Following Rodnight et al. (ref. 92), the FMM_{min} values given in Supplementary Table 9 are the lowest dose component derived by the FMM containing more than 10% of the grains in the dose population. That is, if the smallest dose component contained less than 10% of the dose population the next youngest component is given. The number of components that were used to fit the data was determined using the log likelihood (llik) and the Bayesian Information Criterion (BIC (ref. 93)). Because of the presence of negative dose estimates we could not directly apply the FMM to the nine samples given above. We can compare the FMM_{min} doses with the IEU doses for 19 samples (i.e. dose distributions with no negative dose estimates). The FMM_{min} doses tend to be larger than or consistent with the IEU

doses (average FMM_{min}/IEU=1.7±0.2, n=19); only 74% of the dose estimates are consistent with each other at two standard errors. Because of the good agreement between the MAM and IEU doses, we consider the FMM_{min} doses to be less reliable. In the following discussion, we choose to consider the IEU ages only, because we could determine these for the largest number of samples.

The minimum ages (determined using the IEU) result in significant age reversals in all cores. In core GS-11 an unrealistically young IEU age of 0.09±0.07 ka is obtained for sample 091901 taken from a depth of ~2 m. Of even more concern is the unrealistic young minimum age of 0.4±0.2 ka for sample 091904 (GS-11) taken at a depth of ~25 m. However, all the unrealistic young ages are all obtained from the dose distributions containing negative dose estimates. As stated above, we regard it as physically very unlikely that a significant fraction of modern grains were transported to a depth of 25 m in these cores.

A comparison of CAM and IEU ages gives useful insights towards understanding the meaning of minimum ages in these samples. In Supplementary Figure 19 we plot the (CAM – IEU) difference as a function of CAM age and it is clear that there is a correlation. All data can be well represented by a straight line with a slope of 0.62±0.04. This observation can be interpreted in two ways: (1) the absolute dispersion in the data gets bigger with age (e.g., for a constant relative dispersion), and the minimum age is just sampling an edge of such a distribution. This scenario is very likely for a situation such as ours in which all sources of dispersion have not been accounted for and therefore the uncertainty budget is not complete. (2) Alternatively, the difference between the CAM and the IEU represents an off-set caused by presumed incomplete bleaching, but this premise leads to the absurd conclusion that the degree of incomplete bleaching depends on the subsequent time of burial of the sample. We therefore reject this latter premise and rather interpret the observed trend to be an artefact of the application of minimum age models to those wide distributions for which the leading edge does not arise from an extrinsic phenomenon such as the degree of bleaching, but rather is an inherent part of a distribution whose central tendency is well represented by CAM. The fact that the two aeolian samples (091905 and 111909 from GS-10 and GS-11, respectively) fall on the same line as the fluvial samples further strengthens this view. Murray et al. (ref. 32) observed a similar relationship between MAM and CAM for a range of previously published samples and these authors concluded that the application of MAM without the clear identification of the physical process underlying the dose distribution is unjustified.

The inset to Supplementary Figure 19 shows the normalized difference between CAM and IEU (normalized by the CAM age) as a function of the proportion of the presumed well-bleached grains identified by the IEU (i.e., the proportion of grains used to derive the IEU age). All points are consistent with a straight line with a slope of 0.009±0.001 supporting our conclusion that the IEU does not identify an independent well-bleached population, but rather just extracts a part of it.

Based on this analysis we do not regard minimum age modeling of the data from these samples as physically meaningful.

Most prominent finite mixture model (FMM_{prom}) ages In the decision tree of Bailey and Arnold[84] it is implicitly assumed that natural dose distributions are normally distributed. However, Arnold et al. (ref. 81) argue that natural dose distributions are log-normal and that the skewness should be calculated using logged dose values. Arnold and Roberts (ref. 73) propose a dose estimation decision tree based on the relative OD as well as the log-weighted skewness. If we apply this decision tree to our samples we conclude that the FMM should be

applied to all samples except 111910, and -12 for which the CAM should be applied (based on the llik and the BIC score the FMM predicts that the CAM is more likely to be applicable than the FMM). In the past decade the FMM has widely been applied to samples suspected to be affected by post-depositional mixing[94]. The FMM identifies discrete dose components within a dose distribution; the most prominent dose component (FMM_{prom}) is usually assumed to be that relevant to the burial age, and this is calculated by dividing FMM_{prom} by the average dose rate[72,95-101]. It has also been argued that such dose components may arise from discrete dose-rate components, and some have suggested dividing FMM_{prom} by a modeled dose-rate component [102,103], but Guérin et al. (ref. 80) have pointed out errors in this latter approach. Although Galbraith (2015) (ref. 104) has criticised one of the several arguments raised by Guérin et al. (ref. 80), he nevertheless concludes that their other arguments may be valid. In our view, Guérin et al. (ref. 80) criticisms remain essentially unchallenged and this model should be avoided – a view now supported by the original authors of the dose rate component model[105]. The FMM_{prom} ages (using average dose rates) for all samples are given in Supplementary Table 9 (all non-positive dose estimates have been arbitrarily removed prior to running the FMM). The uncertainty (1 se) on individual ages range between 7 and 32% with an average uncertainty of 12.4±0.9% (n=30). The detailed results of the FMM are given in Supplementary Table 10. Here the number of components identified is given by k and p is the proportion of grains attributed to a given component. The FMM identifies a single component (i.e. $p = 1$) for three samples (111906, -10 and -12), and two and three components for 21 and 6 of the samples, respectively. Of the 21 samples with two dose components, 10 samples each contain, within 1 standard error, ~50% of the dose estimates (i.e. p is consistent with 0.5), making it difficult to decide which component is the most prominent one; we have chosen the component with $p>0.5$.

The most prominent dose component contains on average only 65±2% (n=27, not including the three samples where the FMM predicts that CAM is a more appropriate model) of the total accepted grain population implying that we have considerable (and in our view, unrealistic) post-depositional mixing in the majority of these samples. The average ratio of the FMM_{prom} to the CAM ages is 1.25±0.08 (n=27). In the following description of the apparent chronostratigraphy of the individual cores, we make use of the ages derived from FMM_{prom} (or CAM for the samples from GS-7).

In core SRH-5A, the three FMM_{prom} ages are all consistent with an average age of 20±3 ka. The age of the uppermost coarse/medium sand sample (111922) is 25±3 ka.

For both samples in core GS-7, the FMM predicts that the CAM is likely to provide the best age estimate (see above).

In core GS-10, the FMM_{prom} ages increase smoothly with depth until about 20 m; below this the ages are ~50 ka (see Supplementary Figure 11e). In this core, we appear to have two age inversions. The first inversion occurs for sample 111903, where the FMM identifies two components and gives a FMM_{prom} age of 10.1±1.1 ka (p=58±9%), which appears to be too young. However, if we choose the second component of 33.4±4.0 ka (p=42±9%), the inversion disappears. Thus, one could argue that the older component should be chosen based on stratigraphic consistency. In any case, the choice of the largest component is arbitrary. If one postulates gross post-depositional mixing between sedimentary units, there is no a priori reason that grains from the original unit must always be in the majority. Thus, it is possible to argue that the age of the uppermost medium sand sample (111903) is 33.4±4.0 ka. The second inversion occurs for sample 111906, where the FMM predicts a single dose component giving an age of 29±2 ka. As stated previously, we have added 30% additional uncertainty onto individual dose estimates to account for intrinsic sources of variability (see

section Single-grain dose recovery experiments). However, if one were to add 15% instead of 30% the FMM predicts two components, each containing ~50% of the grains, giving ages of 20±3 ka (p=47±15%) and 41±4 ka (p=53±15%), respectively. The average age of the samples taken from depths >18 m is 48±3 ka, so choosing the larger component removes this inversion. However, none of the beta dose recovery experiments resulted in dose distributions with ODs of ~15%, so we do not think it is justified to change the additional uncertainty parameter to 15%. It has been suggested that the appropriate additional uncertainty parameter can be determined by optimizing the llik and BIC scores provided by the FMM (ref. 93). In the following we test this approach by varying the additional uncertainty (in addition to the uncertainty assigned to individual dose estimates based on Poisson statistics and curve fitting uncertainties) between 5 and 60%. For sample 111906 it is not straightforward to determine the optimum uncertainty parameter as the llik score fluctuates considerably and the BIC score decreases smoothly for an additional uncertainty larger than 5% (see Supplementary Table 11). If we choose the optimum scores to occur at 25% then the resulting FMM_{prom} age is 36±5 ka (p=71±24%). This age is not significantly different from the result obtained using 30% and does not fall in stratigraphic order. In Supplementary Table 11 we show the FMM results for 16 of the samples obtained using a range of additional uncertainties. If we choose the additional uncertainty parameter based on the highest llik score and the lowest BIC score, the additional uncertainty parameters range between 15 and 50%. The ratio of the FMM_{prom} age determined using the optimized uncertainty parameter to the FMM_{prom} age using an additional uncertainty of 30% is 1.01±0.05 (n=16) implying that no significant benefit is obtained by optimization. Only for one sample (111909) is the individual ratio not consistent with unity at two standard errors. Here, the FMM predicts an additional uncertainty of 50% and two dose components with ages of 24.7±3.1 ka (p=51±10%) and 72.1±8.7 ka (p=49±10%; see Supplementary Table 11). Using an additional uncertainty of 30%, the FMM identifies three components (see Supplementary Table 10 and Supplementary Table 11) and predicts an age of 42±7 ka. Thus, for this sample one could argue for an age ranging between ~25 and 70 ka despite the fact that this sample is of aeolian origin.

In core GS-11, the FMM_{prom} ages (30% additional uncertainty) broadly appear to increase with depth although samples 101906 and 091906 appear to be too young giving ages of ~13 ka. However, for both samples the FMM identifies two components in almost equal proportions. If we choose the largest dose components we obtain ages of 34±11 ka (091906) and 38±6 ka (101906) and the FMM_{prom} ages are all in stratigraphic order and increase smoothly with depth (see Supplementary Figure 11f). Sample 091905, which is aeolian and expected to be much older, gives a FMM_{prom} age of 115±11 ka.

For cores GS-13 and GS-14, all FMM_{prom} ages are again in stratigraphic order and the ages of the uppermost fine- to medium-grained sand samples from the GS-transect are 12±2 ka and 15±2 ka, respectively.

The average FMM_{prom} age for the uppermost fine- to medium-grained sand samples from all GS cores is 12.4±1.3 ka (n=3), which is significantly younger than the average CAM age (17.3±1.4 ka, n=3).

It appears that for more than half the samples the FMM_{prom} gives ages consistent with the CAM ages. However, the results suggest that many of the samples (including those from the deepest aeolian sediments) have been mixed after deposition, so that the original sediment now only represents about half of the total; the model suggests that the other half usually come from sediment that is on average ~25 ka different in age without any significant contribution from intermediate horizons.

Supplementary Note 3: Comparison of quartz and feldspar ages following standard selection criteria

Given the results from the modern analogue sample and the comparatively large apparent age in most of our core samples, we consider it unlikely that incomplete bleaching is a significant issue in this study; it may affect some of the younger ages but as discussed above these are not relevant to the determination of the time when major river flow ceased.

Nevertheless, in the absence of independent age control, we do not know which of our luminescence ages are necessarily more accurate. However, we can use stratigraphic constraints and a comparison of independent chronometers (quartz and feldspar) to identify the more reliable ages.

Multi-grain quartz compared with feldspar In Supplementary Figure 20a we plot all the multi-grain quartz ages against the fading corrected feldspar ages obtained using the IR_{50} signal. The closed symbols (n=17) represent quartz ages which we term 'reliable' (<15% of aliquots giving an unbounded dose) whereas the open symbols (n=20) represent minimum ages (>15% unbounded dose estimates). The dashed line represents the 1:1 line and the shaded band around it represents ±10%. The inset shows the same data for feldspar ages less than ~35 ka. Although there is considerable scatter around the 1:1 line, the average ratio of all quartz to feldspar ages is 1.04±0.04 (relative σ=26%; n=37). The slope of the fitted (solid) line in Supplementary Figure 12a is 0.90±0.03. If we include only the 17 reliable quartz ages, the average ratio is 1.07±0.05 (relative σ=19%; n=17). These two quartz/feldspar ratios are both indistinguishable from unity, although the standard deviation is slightly smaller when using the "reliable" quartz ages. The quartz fast component and the IR_{50} feldspar signal are known to bleach at significantly different rates in daylight (~1 order of magnitude difference). This, taken together with the results from the modern analogue and the age agreement discussed above, strongly suggest that both minerals were bleached well enough at deposition; this conclusion is consistent with published observations[32]. This agreement gives additional support to our conclusion that minimum age models are not relevant for our data, despite the predictions of the Bailey and Arnold's (ref. 84) decision tree.

Single grain quartz (CAM) compared with feldspar In Supplementary Figure 20b, we plot the single-grain CAM quartz ages as a function of the feldspar ages. In the absence of significant incomplete bleaching and post-depositional mixing the single grain CAM is normally expected to give the most accurate ages. However, the CAM ages significantly underestimate the feldspar ages: the average quartz CAM to feldspar age is 0.67±0.05 (n=30) and the slope of the line in Supplementary Figure 12b is 0.46±0.02. Only 7 of the 30 ratios are consistent with unity within 2 standard errors. Given that the feldspar ages on average are ~70% older than the CAM ages; it is very difficult to attribute this disagreement to incomplete bleaching of feldspar as any effect of bleaching should, on average, be additive, not proportional. Moreover, the two oldest samples (111909 and 091905) known to be of aeolian origin, also appear to show that single-grain CAM quartz age underestimates the feldspar age by >40%. One explanation could be over-correction for anomalous fading in feldspars. The average fading correction is ~40% (based on a g_{value} of ~3.5 % per decade); this is a typical value for the IR_{50} signal[30,106]. Even if we assume that these samples do not suffer from any anomalous fading (despite the laboratory measurements), the average slope for all the samples is only increased to 0.65±0.01 (n=30, data not shown). However, the evidence for anomalous fading is unambiguous and we do not consider it likely that such over-correction is the cause of the difference between the single grain CAM ages and the feldspar ages. From these data alone it is difficult to decide which of the two data sets is

likely to be the more accurate, although the feldspar ages suffer less from stratigraphic inversions than the quartz ages. However, this analysis does not take into account the effect of bias resulting from the rejection of grains in dose saturation.

Single-grain (FMM) compared with feldspar and interpretation of FMM results In Supplementary Figure 20c, we plot the single grain FMM_{prom} quartz ages against the feldspar ages. The slope of the linear fit to these data (excluding the extreme outlier, sample 111909 open symbol) is 0.76±0.02 (n=29) and the average quartz to feldspar age ratio is 0.81±0.06 (n=29). There is significant scatter in these data and the agreement is not as good as for standard multi-grain aliquot quartz measurements. The overall tendency is that the FMM_{prom} ages are smaller than the feldspar ages. This is particularly pronounced for the deepest sample in GS-10 (111909) which is of aeolian origin. The FMM identifies three components for this sample with the most prominent component based on 47±7% of the grains and giving an age of 42±7 ka. The component with the largest dose contains 26±7% of the grains and gives an age of 104±13 ka. The feldspar age for this sample is 150±6 ka, so even if one were to choose the larger dose component for age estimation (despite the fact that this would not be advocated in the literature), the FMM_{prom} age would significantly underestimate the feldspar age. At this stage in the argument we cannot identify whether the feldspar age is in error or the single grain quartz FMM_{prom} age underestimates the deposition age.

The most widespread use of the FMM (refs. 73,94,99) is to identify discrete dose components arising as the result of post-depositional mixing. However, for >30% of the samples investigated here, the main dose component contains less than 60% of the grain population; this implies that more than 40% of the grains in the unit of interest have migrated from one or two discrete units often lying at a considerable distance (in some cases above, in some below) from the layer of interest.

Three examples serve to illustrate that this is stratigraphically extremely unlikely. Firstly, in core SRH-5A, the FMM gives an older age of 25±3 ka (54% of grains) for sample 111922 (429 cm depth). The younger component of the mixture is 7.2±1.4 ka (46% of grains). This sample is at the top of the grey fluvial sand succession and is immediately overlain by floodplain clay and silt (Fig. 8 and Supplementary Fig. 5). Thus, the younger component can only be sourced in the floodplain sediment. To dilute the original fluvial sand such that today 46% of the deposit is made up of grains derived from floodplain sediment would require (a) a large-scale process of very differentiated grain transport preferentially selecting only the sand grains from the overlying clay-silt, and (b) destruction of the distinct grain size transition between the two sedimentary facies. It is implausible that there has been mixing between the fluvial sands and the fine-grained deposits, because the latter do not contain any admixed fluvial medium- to coarse grained sand. Secondly, in core GS-11, the FMM gives an older age of 56±7 ka (51% of grains) for sample 091904 (2495 cm). The younger component of the mixture is 12±2 ka (49% of grains). This sample was taken from a grey fluvial sand unit and also lies immediately below a floodplain clay and silt unit (Fig. 5). In this case, the younger component could in principle be sourced from any of the fluvial sand units overlying this floodplain unit (see minor components in GS-11 in Supplementary Table 10). However, sediment of consistent age (13±2 ka) is not a major component of any deposit until a depth of ~920 cm (sample 101906) (Fig. 5). This more likely source unit is separated from sample 091904 by >15 m and four stratigraphically distinct floodplain clay and silt units. Again, to dilute the original fluvial sand such that today 49% of the deposit is made up of the material sourced from at least ~9 m depth would require the destruction of a number of grain size transitions observed in the sedimentary succession between the two units. Mixing of grain components between these two depths without homogenization of the succession is highly implausible. Finally, in core GS-13, the FMM gives an older age of 20±3 ka (71% of grains)

for sample 111914 (507 cm). The younger component of the mixture is 5±2 ka (29% of grains). This sample was taken from a grey very fine sand bed and also lies immediately below a floodplain clay and silt unit (Figs. 5 and 7; Supplementary Figs. 2 and 3). The youngest FMM age in the grey very fine sand (sample 111913; 245 cm depth) overlying this floodplain unit is 12±2 ka (68% of grains) which is too old to source the younger component of sample 111914. Thus, the potential source must lie closer to the surface, in the undated aeolian or red silty clay units. To dilute the original fluvial sand such that today 29% of the deposit is made up of the material sourced from <245 cm depth would require the destruction of at least three distinct grain size breaks separating the two units. In particular, a silty claystone unit that is interstratified between the sand units of samples 111913 and 111914 (and which represents about 1 m of section between 4 and 5 m depth bgl) does not contain any sand grains (Supplementary Fig. 2), thus invalidating a post-depositional mixing explanation. Thus, the presence of distinct grain size breaks in the stratigraphic succession clearly argues against any large-scale sediment mixing in this core as there is no evidence of grain size homogenization.

Animal burrows are frequently mentioned in justifying such results, but such gross mixing is undifferentiated and cannot explain the result from SRH-5A and, in any case, would have completely destroyed the observed grain size transitions observed in all examples. In summary, we conclude that physical mixing of these stacked units can be safely dismissed as an explanation for the apparent dose mixtures observed in our data.

Another explanation for the presence of components in a FMM analysis is that they are a consequence of extreme dose rate heterogeneity. However, this explanation can be dismissed with confidence, as Guérin et al. (ref. 80) have pointed out, modeling has shown that discrete dose rate populations do not exist except in very unusual conditions, and these do not apply to well mixed and homogeneous sands such as found in our samples.

The FMM components could arise from intrinsic characteristics such as differing luminescence properties. Although at this point it is difficult to see why some quartz grains should result is such a completely different dose estimate from other grains, but the possibility that, for instance, sensitivity changes during the measurement of the natural signal may be systematically different between two groups of grains cannot be dismissed.

Finally, the various components may be a statistical artifact resulting from the application of the model to a very broad dose distribution with unrealistic estimates of the intrinsic over-dispersion. In the Supplementary Table 11 we investigate the effects of using different estimates of the intrinsic over-dispersion on the FMM ages; from these data, it appears that the FMM predicts a single dose distribution if the OD is set to ~50%.

Given the unlikely results obtained using the Finite Mixture Model, we are unable at this stage in the argument to decide whether the most accurate ages are obtained from quartz single-grained, from quartz multi-grain aliquots or from multi-grain feldspar aliquots. In Supplementary Note 4 we present two additional single-grain selection criteria to address this problem.

Supplementary Note 4: Additional single grain rejection criteria

As discussed in section Single-grain OSL dose response curves, the D_0 values of the dose response curves for individual grains vary significantly between grains (see Supplementary Figure 17b), and grains which are rejected due to saturation tend to have lower D_0 values than those which are accepted. The fact that there is a difference in D_0 values between grains rejected only due to saturation and accepted grains is potentially of serious concern. For

instance, consider a group of grains all with a D_0 value of 75 Gy extracted from a sample with a dose of 100 Gy (see Supplementary Figure 17c). For ease of calculation we have assumed the saturation value *A* (see section B.4) to be unity. Suppose that the L_n/T_n values are normally distributed with a standard deviation of 10%. This distribution of L_n/T_n values will be centered on ~0.74. If we then extract 1,000 random L_n/T_n values from this normal distribution and interpolate them on to the dose response curve, we obtain a skewed dose distribution (grey histogram in Supplementary Figure 17c). The degree of skewness will vary depending on the relationship between the given dose and the D_0 value, but as Murray and Funder[37] pointed out, the median of this dose distribution will be closer to the given dose than the average. However, let us now suppose we have a group of grains all with dose response curves with a D_0 value of, say, 25 Gy. Thus, the L_n/T_n distribution will be centered on ~0.98 just 2% lower than the saturation value. Again, we assume that it is a normal distribution with a standard deviation of 10%. If we randomly select 1,000 values from this L_n/T_n distribution and interpolate them onto the dose response curve with a D_0 value of 25 Gy, we obtain dose estimates from only approximately 50% of the L_n/T_n values. These dose estimates are shown as the white histogram (and arise from the white part of the L_n/T_n distribution centered on 0.98). As can be clearly seen from Supplementary Figure 17c, this dose distribution is highly skewed with a mode of approximately 50 Gy. Thus, we deduce that it is entirely possible that at least some of the grains which have been accepted into our natural dose distribution actually come from a distribution of grains with low D_0 values, and so which have absorbed a saturation dose; inclusion of such grains will bias it to low doses. In an attempt to circumvent this problem, we suggest all grains with low D_0 values (compared to the natural dose) should be rejected. Gliganic et al. (ref. 72) suggested removing all dose estimates with a D_0 value less than 25 Gy, but this was suggested solely because of their inability to obtain acceptable dose recovery ratios for their samples. We suggest a more fundamental reason for using such a criterion.

Thomsen et al. (ref. 40) investigated the effect on single-grain dose estimation of rejecting individual grains based on their D_0 value in beta- and gamma dose recovery experiments using four known-age sedimentary quartz samples from the Bordes-Fitte rockshelter in France. These authors found that in their gamma dose recovery experiments a dose dependent decrease in the dose recovery ratio was eliminated by rejecting all grains with $D_e<D_0$. Applying this approach to the natural dose distributions led to agreement between the expected dose and the measured dose. To determine an appropriate D_0 cut-off value we have calculated the average (both arithmetic and CAM) dose as a function of D_0 > x Gy for sample 111909 (see Supplementary Figure 17d). For x = 0 Gy all grains are included in the average irrespective of D_0 value. For x = 50 only grains with a D_0 value greater than 50 Gy are included. The average dose increases with increasing D_0 cut-off value until only grains with a D_0 value greater than 100 Gy are used. Increasing the D_0 cut-off value further does not result in a significantly different average dose. The CAM dose is increased from 94±5 Gy (n=229) to 136±9 Gy (n=120), but the relative OD does not change significantly, i.e. from 72±6% to 64±7%. This is in contrast to the observation by Gliganic et al. (ref. 72) who reported a reduction in relative OD from ~30% to ~15% in their dose recovery experiments. In line with the observations of Thomsen et al. (ref. 40), we choose the appropriate D_0 cut-off value to be equal to the sample average D_e.

For the OSL signal to be a useful dosimeter it is important that it is both optically and thermally stable over geological time scale. The fast component in quartz OSL has been shown to derive from the 325 °C TL peak which has a life time of ~10^8 y (ref. 12). However, other less thermally stable traps may contribute to the OSL signal[51,60] but it is anticipated that most of these contributions are eliminated by the chosen thermal pre-treatment (preheat).

However, Moskva and Murray (ref. 107) reported that when they sensitized the fast component in their previously insensitive samples a large fraction of their resulting fast component OSL signal was unstable. An unstable rapidly decaying OSL component has also been reported in heated quartz[108]. In section B.1.3 we showed for large multi-grain aliquot measurements that a preheat of 260 °C, 10 s and a cutheat of 220°C was an appropriate thermal treatment for these samples. However, it is possible that this may not be true for every grain[109]. To investigate the thermal stability of individual grains we measured pulse anneal curves for 2,400 grains from sample 111909. Prior to these measurements, we measured the natural doses in these grains as described in section OSL Measurements. The pulse anneal curves were measured using the SAR procedure in which the entire signal is measured for each preheat temperature. The preheat temperatures ranged from 200 to 340 °C and were held for 10 s. After the final measurement at 340 °C four repeat measurements at 220, 260, 280 and 320 °C were made. Any sensitivity change was monitored using a test dose with a preceding cutheat of 220 °C. High temperature blue stimulation at 280 °C for 40 s was inserted after each L_x and T_x cycle to minimize recuperation effects. The regeneration and test doses were both 50 Gy in this experiment. The shapes of these pulse anneal curves varies considerably. In Supplementary Figure 17e we show representative pulse anneal curves for four selected single grains from sample 111909 to illustrate this variability. The pulse anneal curve for grain D24G43 (circles) appears to be similar in shape to published data from multi-grain aliquots[51,110], whereas the other three grains appear to be less thermally stable to varying degrees. The apparently least thermally stable grain (D2G100, squares) has the smallest estimate of equivalent dose of 46±18 Gy, whereas the most apparently thermally stable grain (D24G43, circles) has the highest equivalent dose of 254±13 Gy (as observed by Fan et al. (ref. 109)). Although it probably would be advantageous to measure pulse anneal curves for every grain to establish the thermal stability on a grain-by-grain basis, we recognize that this would be very labor and time consuming. Instead we propose to make a single measurement using the chosen thermal treatment (in this case a preheat temperature of 260 °C and a cutheat temperature of 220 °C) and the same doses for both L_x and T_x. Since the regeneration and test doses are the same, if the signal is stable one would expect a similar response during both the regeneration and test dose measurement, despite their different prior thermal treatment. Thus, we can subsequently reject grains with a low value of this L_x/T_x ratio (hence forth called the L_{ts}/T_{ts} ratio) due to the possibility of low thermal stability (although of course some of this difference could result from sensitivity change). In Supplementary Figure 17f we investigate the effect of rejecting grains based on the L_{ts}/T_{ts} ratio on the average (arithmetic) dose. If we apply this stability criterion to the dose distribution obtained using the standard rejection criteria ($D_0 > 0$, squares) then we see little effect of rejecting grains based on the L_{ts}/T_{ts} ratio (Supplementary Figure 17f, green squares). However, if we apply it to the dose distribution only containing grains with dose response curves with $D_0 > 100$ Gy, we see that the average dose increases steadily from ~140 Gy to ~200 Gy for L_{ts}/T_{ts} values greater than 0.8. If we tighten the additional D_0 criteria so to only include dose estimates with D_0 values greater than 200 Gy, we see a similar pattern but the average dose increases to ~330 Gy (CAM dose of ~310 Gy). The shaded band in Supplementary Figure 17f is the quartz dose predicted from measurement of feldspar (Supplementary Note 1). In Supplementary Figure 17a the effect on the dose distribution of applying the $L_{ts}/T_{ts} > 0.8$ criterion in combination with the D_0>100 Gy (light and dark grey symbols) or D_0>200 Gy (only dark grey symbols) criteria is shown. When these criteria are invoked we see that we primarily reject grains with low D_e values. Of the original 229 dose estimates, we are left with just 40 and 9 dose estimates when applying the additional criteria for D_0>100 and D_0>200 Gy, respectively. We have carried out the same analysis for all 30 single-grain dose distributions using a L_{ts}/T_{ts} ratio > 0.8 and determined the D_0 cut-off value

appropriate for each sample (see Supplementary Table 9). The ratio of the doses estimated using the two new additional rejection criteria to the doses estimated using the standard rejection criteria is 1.39±0.08 (n=30) for the arithmetic average and 1.46±0.09 (n=30) for the CAM average. In general, the younger the sample, the smaller the effect of applying these criteria, e.g. for samples with KF ages <10 ka (i.e.101901, -03, 111910, -12) the average ratio of the arithmetic mean is 1.03±0.01 (n=4) and 1.06±0.04 (n=4) for the CAM. In Supplementary Figure 11e and Supplementary Figure 11f we show the derived ages as a function of depth for cores GS-10 and GS-11, respectively.

Although the single-grain quartz dose distributions obtained using the additional rejection criteria generally contain only a few grains (between 4 and 139) it is still worthwhile considering the average effect on the relative OD. The average ratio of the OD calculated for the dose distributions obtained with the new and the standard rejection criteria is 0.70±0.05, i.e., the relative OD is on average decreased by ~30%.

We also applied these additional two rejection criteria to 6 of the dose recovery data sets (111910, -12, -15, 101903 and -06). For sample 111916 we are left with just 3 dose estimates and this data set is subsequently excluded from the following analysis. The average dose recovery ratio for the five samples using the additional rejection criteria is 1.09±0.04 and the average relative OD is 22±3%. These values are completely consistent with the results obtained using the standard rejection criteria for the same five samples, i.e. a dose recovery ratio of 1.01±0.06 and an average relative OD of 27±3%.

In Supplementary Figure 20d we show the average single grain quartz ages obtained using the two additional rejection criteria as a function of the feldspar ages. The slope of the linear fit to these data is 0.98±0.02 (n=30) and the average quartz to feldspar age ratio is 1.04±0.05 (n=30) with 85% of the ratios falling within 2 standard errors of unity. The corresponding numbers for the CAM ages using the additional rejection criteria are 0.89±0.02 and 0.90±0.04. Thus, it would appear that we obtain a good agreement with feldspar if we employ the two proposed rejection criteria, despite the fact that we are left with very few quartz grains in each dose distribution. This gives us sufficient confidence in our feldspar ages to use these as the best estimates of burial ages.

Supplementary Note 5: Comparison of OSL results

We used OSL to obtain burial ages for 52 samples extracted from seven individual sediment cores. We dated these samples using the IR_{50} signal from multi-grain K-feldspar aliquots (2 mm) and the blue/green OSL signals from multi-grain (8 mm) and single-grain quartz aliquots.

The fading corrected IR_{50} feldspar ages are in stratigraphic order except for the two younger fine-grained samples (111912 and 111901/101901) in cores GS-10 and GS-11, respectively. These apparent inversions are also observed in the quartz ages suggesting that these minor inversions of ~1.5 ka most likely result from dose rate measurement problems. The measured dose rates for samples 111912 and 101901 are ~25% higher than those measured for the samples immediately above (111911 and 111901, respectively). If one were to use the dose rates determined for the samples directly above the age inversions would disappear. However, given the small absolute off-set of these inversions (~1.5 ka) and the otherwise good stratigraphic relationship between the feldspar ages, we do not regard these inversions as important.

To assess the importance of any potential incomplete bleaching we dated a modern analogue taken from where the modern Sutlej river leaves the Siwalik foothills and enters the Indo-

Gangetic plain and determined a burial age of ~0.7 ka. Thus, we would regard these samples as probably well-bleached although it must be recognized that the feldspar ages for the very young samples (<10 ka) may be slightly overestimated. Multi-grain quartz analysis of the modern analogue gave a residual dose of 0.9±0.2 Gy (n=24), corresponding to an apparent burial age of ~400 years, which also indicates that the sediments delivered by this fluvial system are likely to be relatively well-bleached. Given the significant difference in bleaching rates between quartz and feldspar (approximately one order of magnitude), we interpret this agreement as indicating that both minerals were well-bleached when deposited.

The ages derived from multi-grain quartz measurements are broadly in stratigraphic order, but with the exception of the two young samples mentioned above as well as samples 091906 and 111916, which both appear too old. However, a significant fraction (23%) of the measured multi-grain aliquots from many samples were in or above saturation on the laboratory dose response curves and thus no bounded dose estimates could be derived. We have arbitrarily chosen only to consider ages to be reliable if we can derive bounded dose estimates for more than 85% of the measured aliquots. The remainder are regarded as minimum ages; of the 37 samples measured only 17 samples are bounded (Supplementary Table 8). For the latter, we obtain a broadly good agreement with the corresponding feldspar ages (Supplementary Figure 20a), although there are two out of 17 samples (i.e. samples 111906 and -23) for which the quartz to feldspar age ratio is more than 3 standard errors away from unity.

To investigate whether or not these discrepancies arose because of intrinsic problems such as poor luminescence characteristics and/or extrinsic processes such as incomplete bleaching or post-depositional mixing, single grain dose distributions were measured for 30 of the 37 samples (Supplementary Table 9). A comparison of all of the single grain CAM ages with feldspar (Supplementary Figure 20b) gives us a systematic fractional underestimate (with no significant intercept on the feldspar axis); this is not consistent with the expected effects of incomplete bleaching. This conclusion is supported by the application of minimum age models to the single grain quartz data which gives unrealistically low ages with several very significant stratigraphic inversions (Supplementary Table 9). By comparison with the CAM ages we also observe an effect of apparent incomplete bleaching which is a constant fraction of age (~60%; see Supplementary Figure 19). This is clearly unrealistic and the minimum ages are dismissed as inappropriate and inaccurate.

It is difficult to explain the differences between the multi-grain aliquot quartz and K-feldspar ages in terms of post-depositional mixing, because such mixing should have affected both ages in a similar manner. Thus, post-depositional mixing may result in stratigraphic inconsistencies but these should not be visible in a comparison of the two methods such as the one shown in Supplementary Figure 20a. Nevertheless, it may be that presumably intrinsic characteristics have led to the discrepancies in Supplementary Figure 20a and it may be possible to identify these “good” and “bad” grains using the FMM. However, application of the FMM results in significantly larger dispersion in the data compared to feldspar than that observed in the multi-grain quartz data, although it must be recognized that in the multi-grain analyses samples that had a large number of aliquots in saturation were rejected completely, whereas in the FMM only grains that did not give bounded dose estimates were rejected (this did not result in the rejection of any entire sample). Nevertheless, when using standard rejection criteria, it remains unclear whether the FMM results or the K-feldspar results are to be preferred, although the mixing implications of the FMM are physically unrealistic.

Finally, we tested two additional single grain quartz rejection criteria based on the shape of the dose response curve and apparent thermal stability. Application of these criteria identified the majority of grains as unable to record accurately doses in the range of interest. The ages based on the few remaining accepted grains compare well with feldspar ages (Supplementary Figure 20d) with no detectable intercept on the feldspar axis and an average ratio of 1.04±0.04 (CAM ratio of 0.90±0.04). This agreement strongly suggests that these single grain quartz and K-feldspar ages should be preferred as the best estimates of deposition age. While we reject the result of the FMM analysis as indicating unrealistic degrees of mixing, the agreement in Supplementary Figure 20d does not necessarily rule out entirely the possibility of some degree of post-depositional mixing which presumably could affect quartz and feldspar to the same degree. Unfortunately, there are not enough single quartz grains identified as reliable dosimeters in the range of interest to allow for the application of the FMM to the accepted dataset. Nevertheless, the stratigraphic consistency of the feldspar data suggests that post-depositional mixing is not likely to have affected the ages significantly.

Given the absence of any suggestion of a systematic difference between the accepted quartz ages (both single grain and multi-grain) and fading-corrected multi-grain feldspar ages, and the fact that residual doses in related modern sediment are negligible, we consider the feldspar ages as the most accurate and precise.

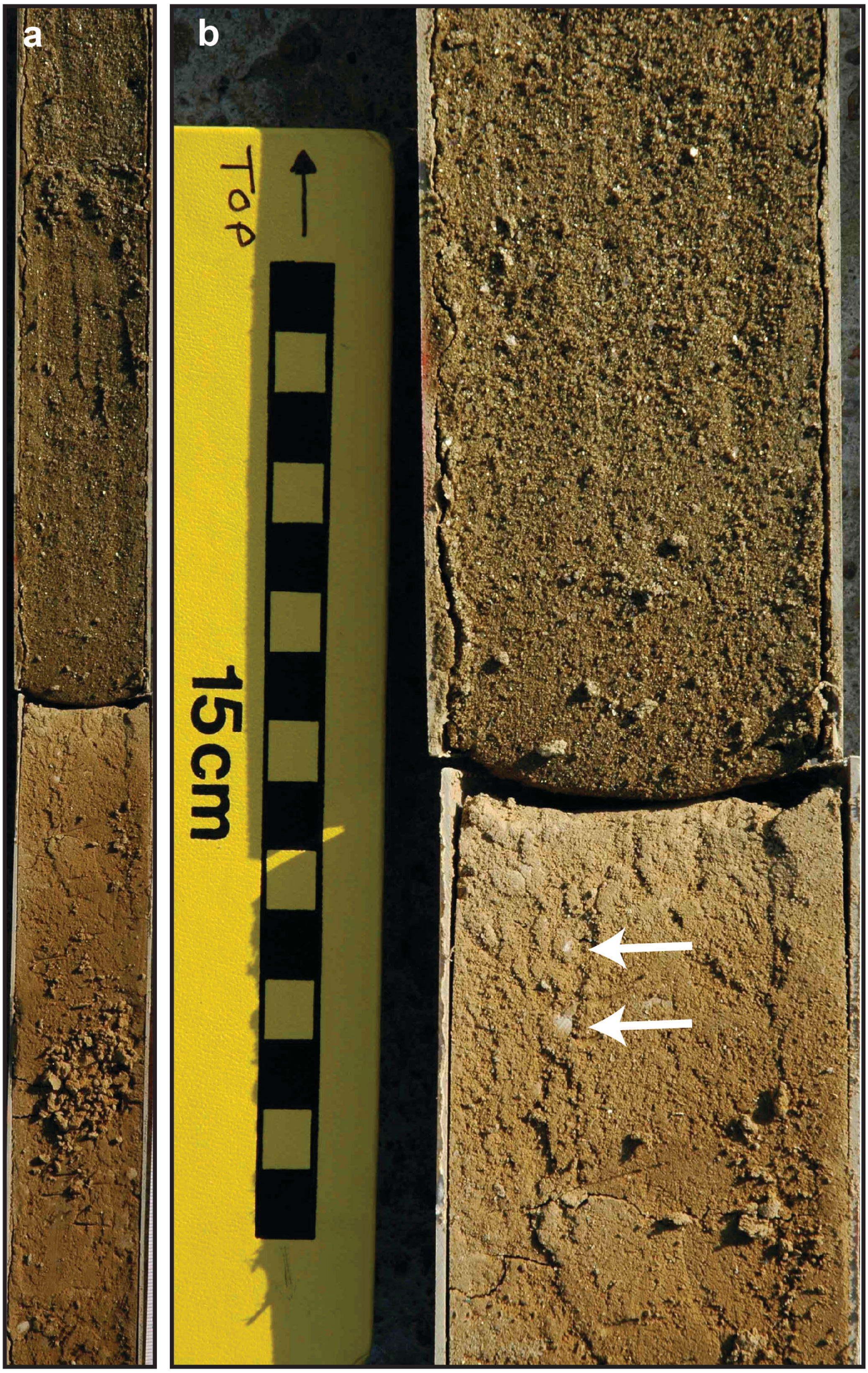

Supplementary Figure 1 Characteristics of sediments at base of GS cores. a, Photograph of core section at base of GS11 core at depth of ~35 m bgl showing abrupt transition from light yellow brown well sorted, fine-grained aeolian sand to dark grey, mica-rich, medium-coarse-grained fluvial sand. **b,** Detail of facies transition. Note the light grey, calcrete nodules at top of aeolian sand (arrowed). The abrupt transition to fluvial sands records an incursion of a fluvial system into the area.

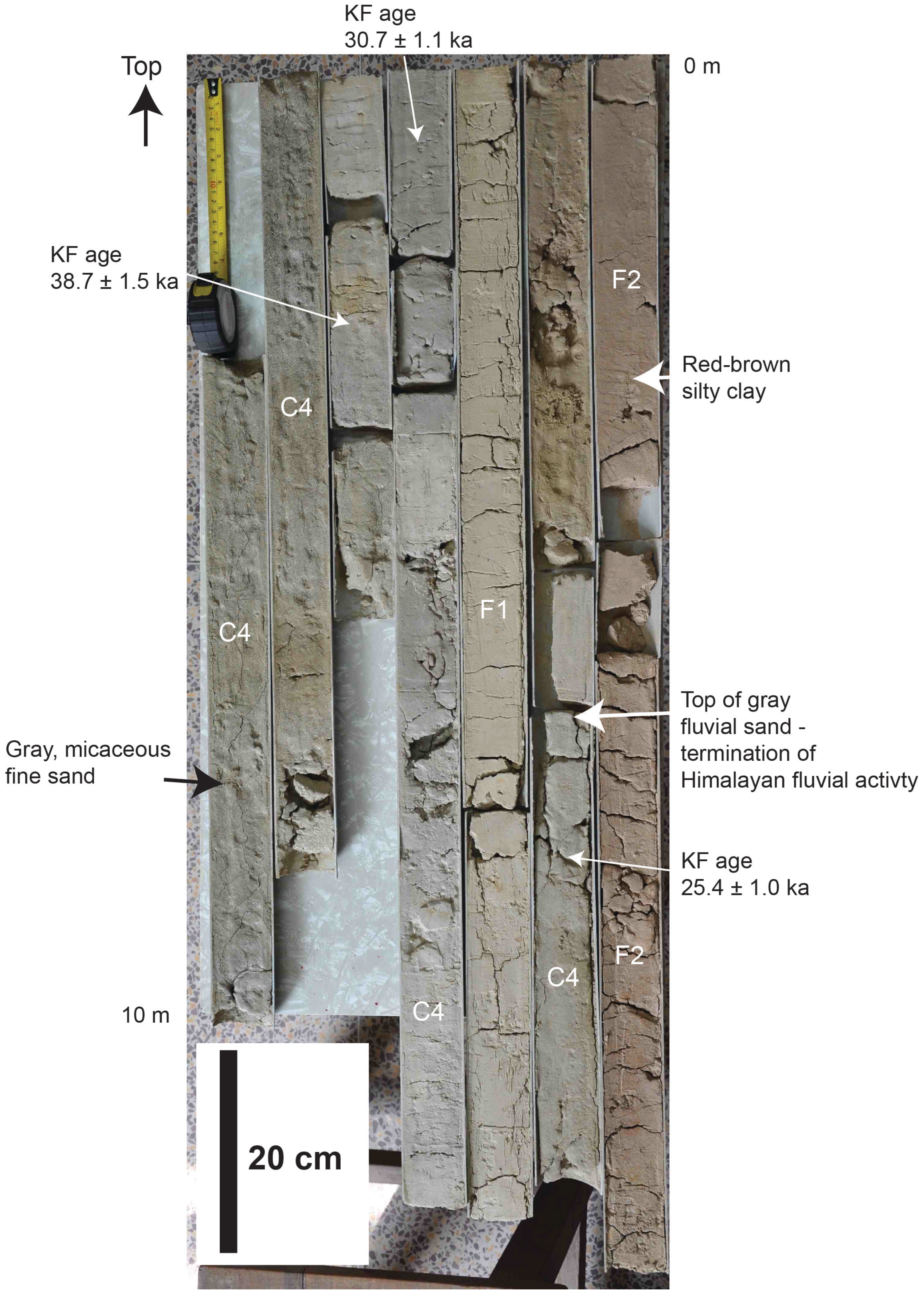

Supplementary Figure 2 Characteristics of sediments in core GS13. Core recovered from GS13 at Kalibangan at a depth of 0 - 10 m bgl. The core is located at the margin of the incised valley. Unconsolidated grey fluvial sands (C4) are present at the base with interstratified fine-grained floodplain sediments (F1). Near the top there is an abrupt transition into brown silts and red-brown silty clays (F2) indicative of very low energy depositional environments and termination of major fluvial activity. K-feldspar OSL ages are indicated.

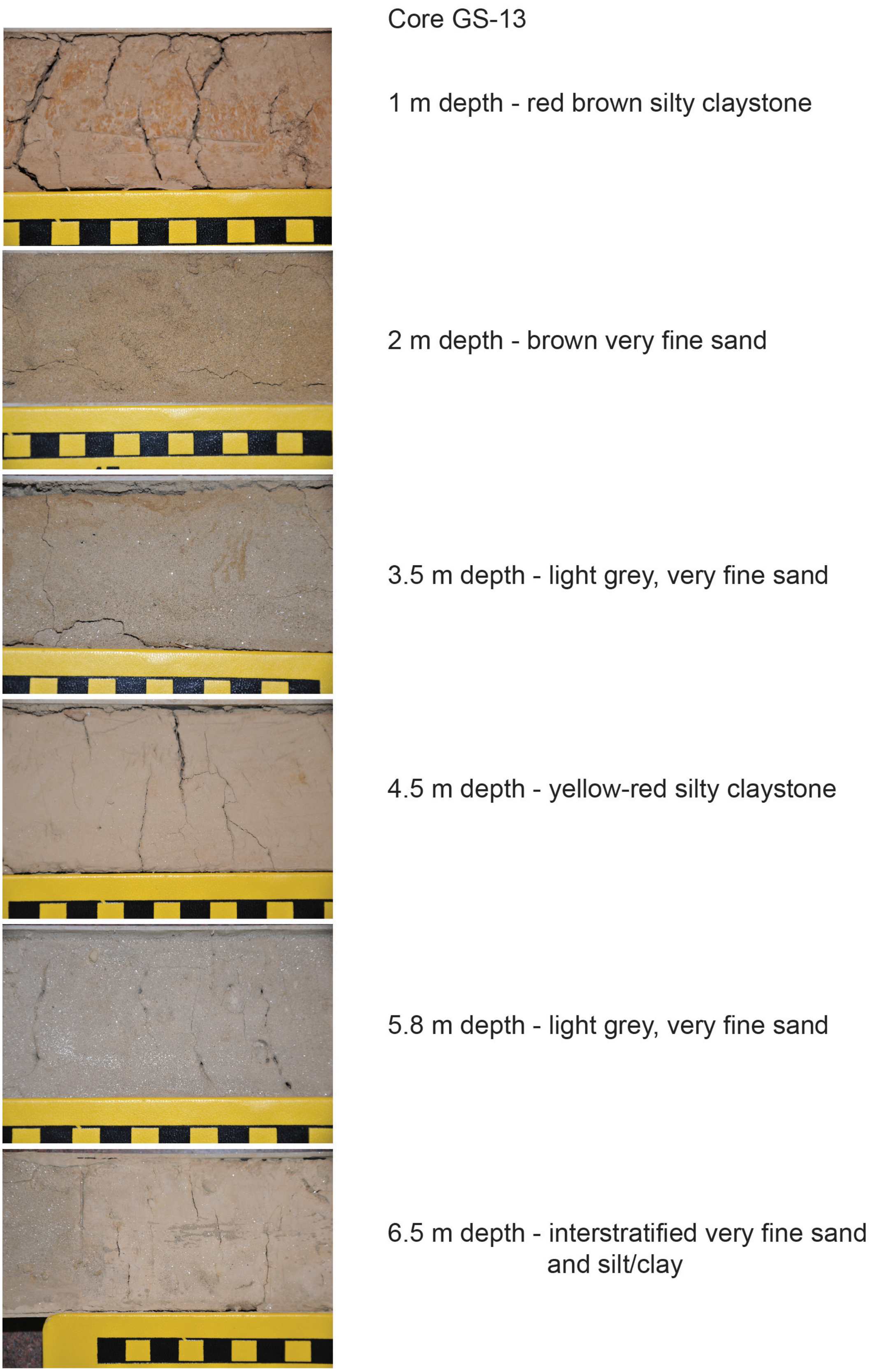

Supplementary Figure 3 Characteristics of sediments in core GS13 Detailed lithology and sedimentary features of core at a depth of 0 – 6.5 m bgl recovered from GS13 at Kalibangan.

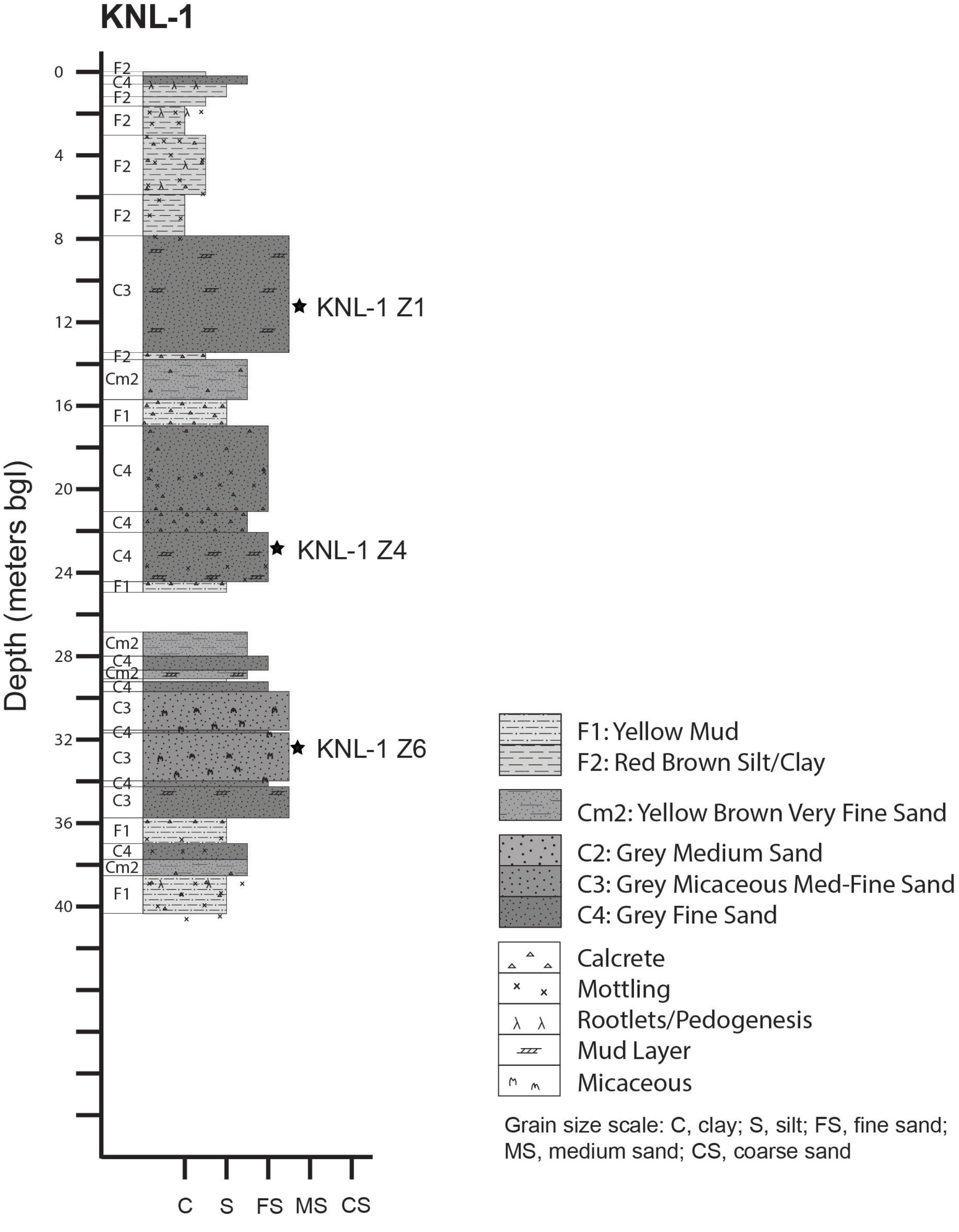

Supplementary Figure 4 Sedimentary log of core KNL1. Sedimentary section in core KNL1 comprises stacked fluvial channel sands with interstratified fine-grained floodplain deposits from base of core to a depth of 8 m bgl. Above this, high-energy fluvial sediments are overlain by low-energy red-brown silt and clay deposits similar to those observed in core GS10 and MNK6, and indicating termination of high-energy fluvial deposition. Depths of detrital zircon samples are indicated.

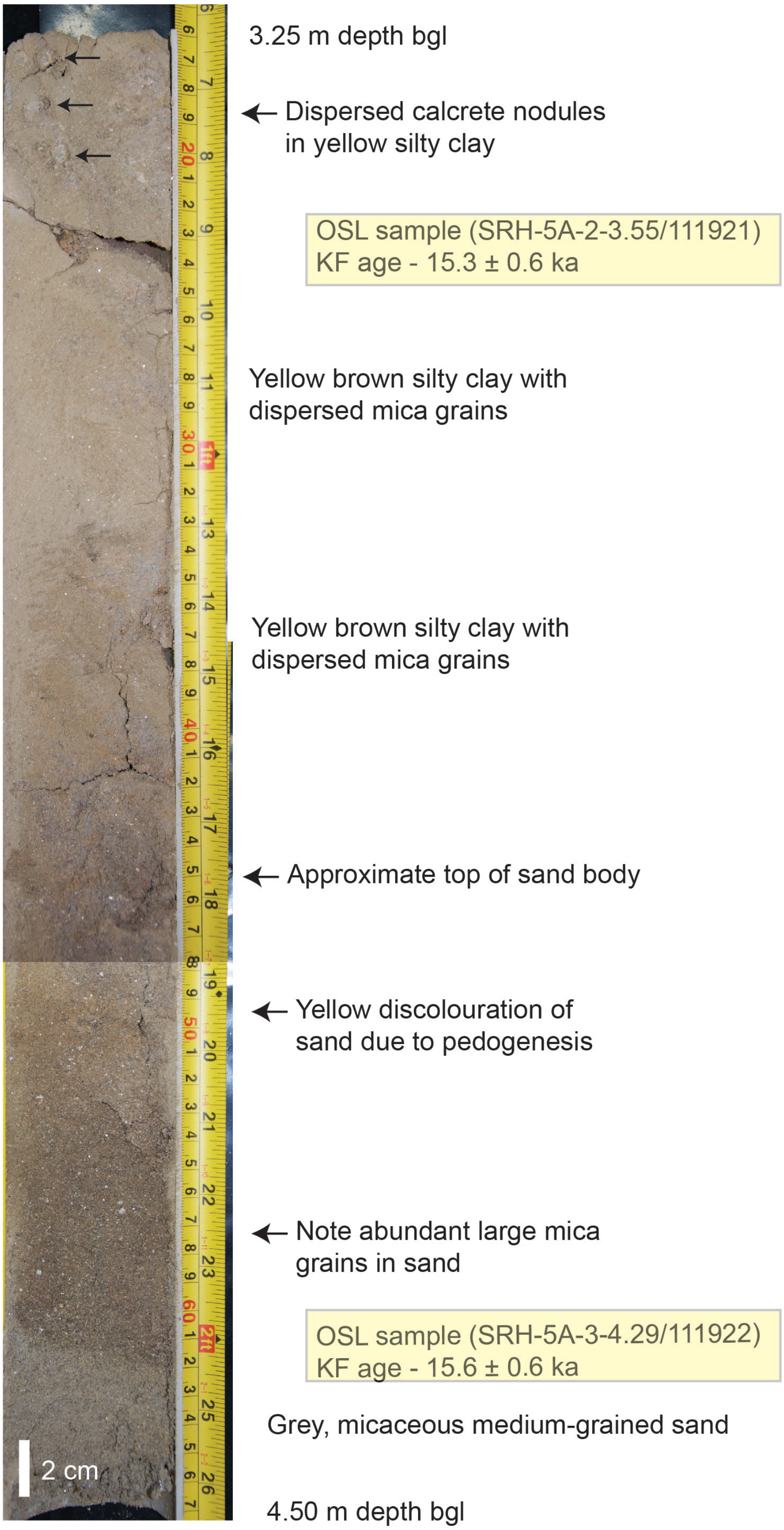

Supplementary Figure 5 Characteristics of sediments in core SRH5 Photograph of sedimentary features in upper part of core SRH5 showing transition from grey, micaceous medium-grained fluvial sands to yellow brown silty clays recording termination of higher energy fluvial deposition at this site. K-feldspar OSL ages are indicated.

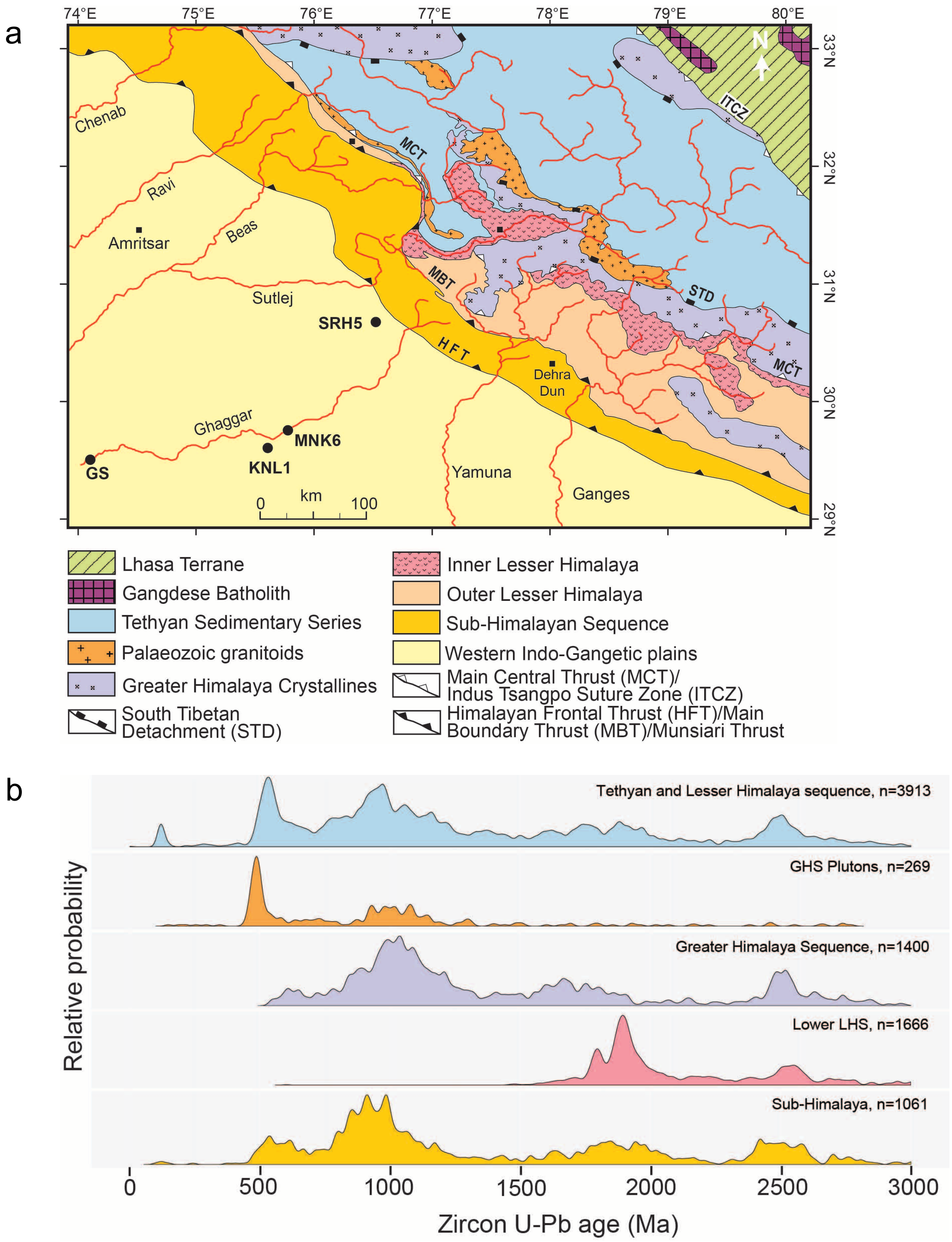

Supplementary Figure 6 Bedrock geology of NW Himalaya and bedrock U-Pb age distributions. a, Simplified geological map of Sutlej River catchment in NW Himalaya along with other major drainages. The map is compiled from Vannay et al. (ref. 111); Webb et al. (ref. 112); White et al. (ref. 113) and Yin (ref. 114) and shows major lithotectonic units and major structures in NW Himalaya with respect to drill sites. **b, U-Pb age distributions of bedrock from different lithotectonic units in the Himalaya.** Probability distributions of U-Pb ages of zircons in different lithotectonic assemblages in the Himalayas. The ages for Tethyan Himalayan Sequence, Greater Himalayan Sequence (GHS), and Lesser Himalayan Sequence (LHS) are from Gehrels et al. (2011) (ref. 115) and references therein. The ages for Sub-Himalaya are from DeCelles et al. (2004) (ref. 116) and Ravikant et al. (2011) (ref. 117).

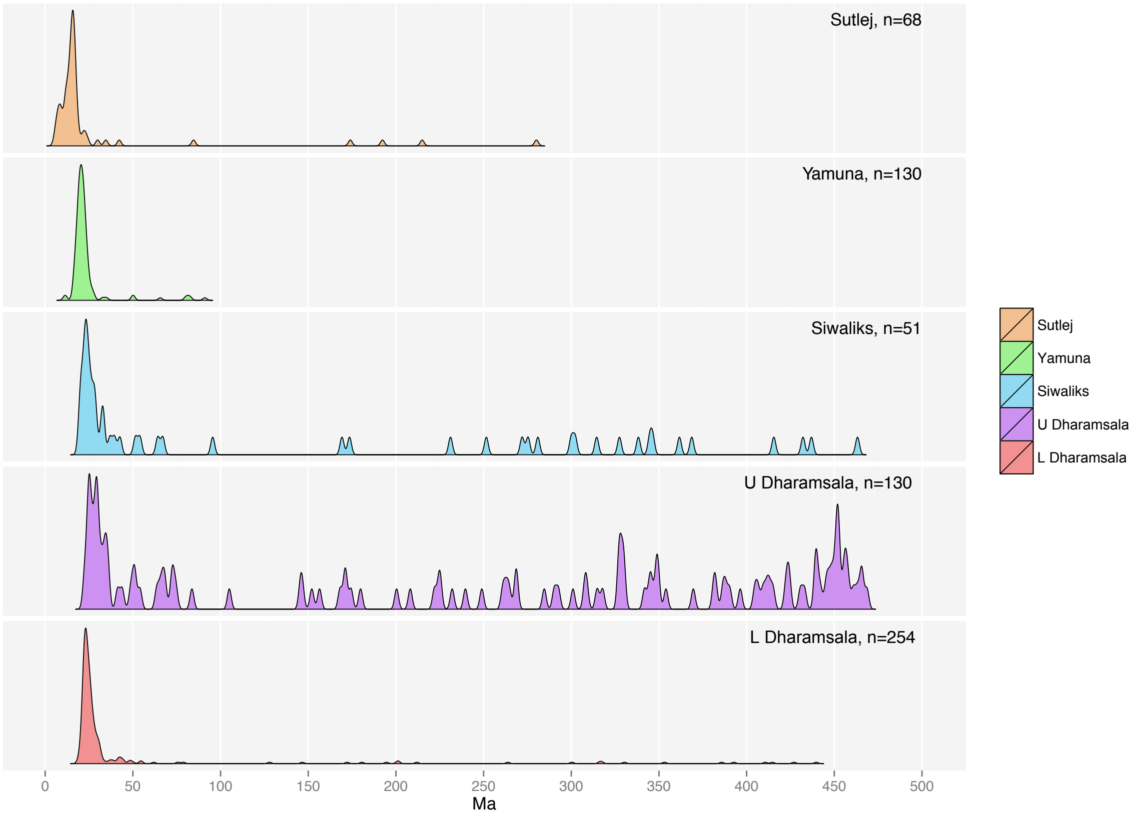

Supplementary Figure 7 ^{40}Ar/^{39}Ar muscovite age distributions of Sub-Himalayan Sequence rocks compared with detrital muscovite ages of Sutlej and Yamuna rivers. Muscovites from Sub-Himalayan Sequence rocks show abundant >30 Ma age distributions that are not observed in the age distribution patterns of detrital micas from our core samples or in the modern Sutlej and Yamuna rivers implying that the grey fluvial sands in Ghaggar-Hakra palaeochannel are not derived from re-working of Sub-Himalayan foreland basin deposits. The presence of <10 Ma age muscovites in core samples and the Sutlej River sample also indicates that the sands are not reworked from the Sub-Himalayan Sequence. Data for Sub-Himalaya from White et al. (2002)[113].

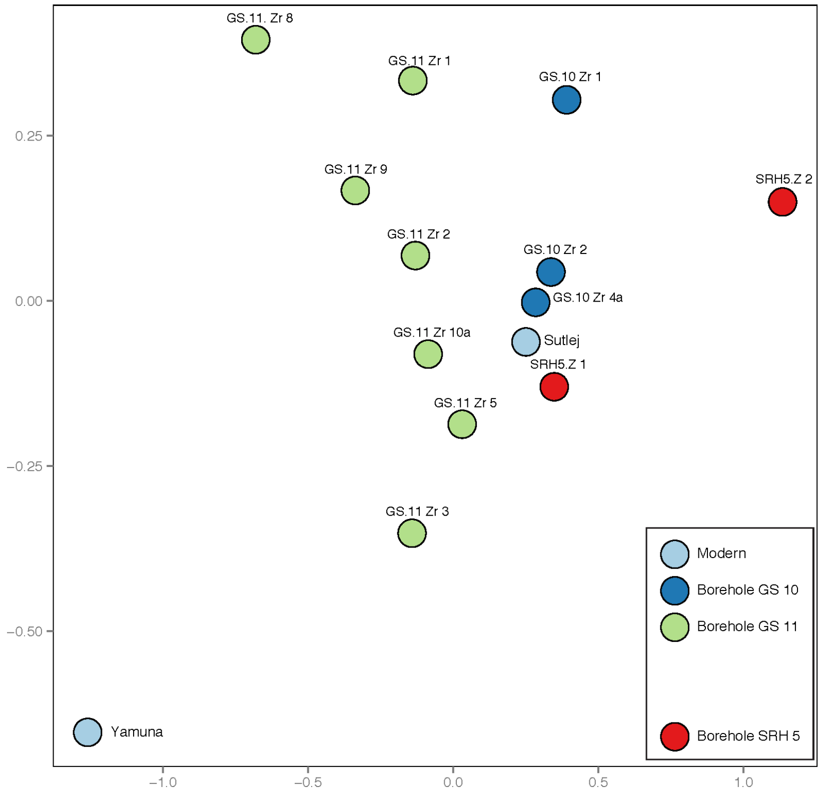

Supplementary Figure 8 Three-way multi-dimensional scaling (MDS) map of the pattern of similarity or dissimilarity for combined detrital zircon U/Pb and detrital mica $^{40}Ar/^{39}Ar$ age distributions. The plot groups samples with similar age distributions, and pulls apart samples with different distributions, using the Kolmogorov-Smirnov (KS) effect size as a dissimilarity measure[118]. Axes are in dimensionless 'KS units' ($0 < KS < 1$) of distance between samples. Detrital zircon and mica samples from cores cluster close to the sand sample from the modern Sutlej River and plot away from the modern Yamuna river sand sample. This demonstrates that the core samples have a similar source to the modern Sutlej River sand sample.

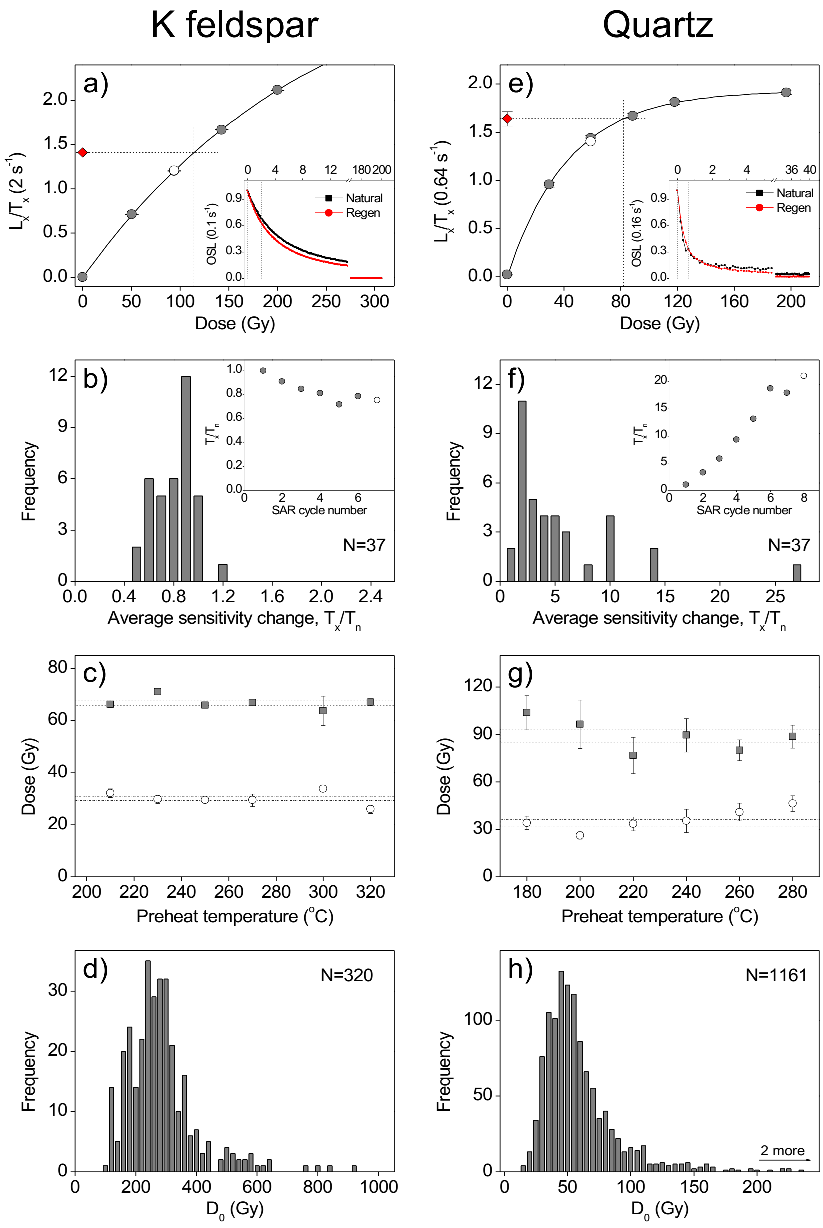

Supplementary Figure 9.

K-feldspar (a,b,c,d) and quartz multi-grain (e,f,g,h) aliquot luminescence characteristics. a) and e) Typical dose response curves (circles) for an aliquot from sample 101906. The recycling point is indicated as an open symbol. The insets show the natural and a laboratory regenerated OSL signal. The dashed vertical lines indicate the signal summation interval. b) and f) Frequency histograms of the average sample total sensitivity change (37 samples). The insets show the sensitivity change as a function of SAR cycle number for the aliquot given in a) and e). c) Preheat plateau data for samples 111902 (circles) and 111913 (squares). d) and h) Frequency histograms of the D_0 values for individual multi-grain aliquots. g) Preheat plateau data for samples 111902 (circles) and 101906 (squares).

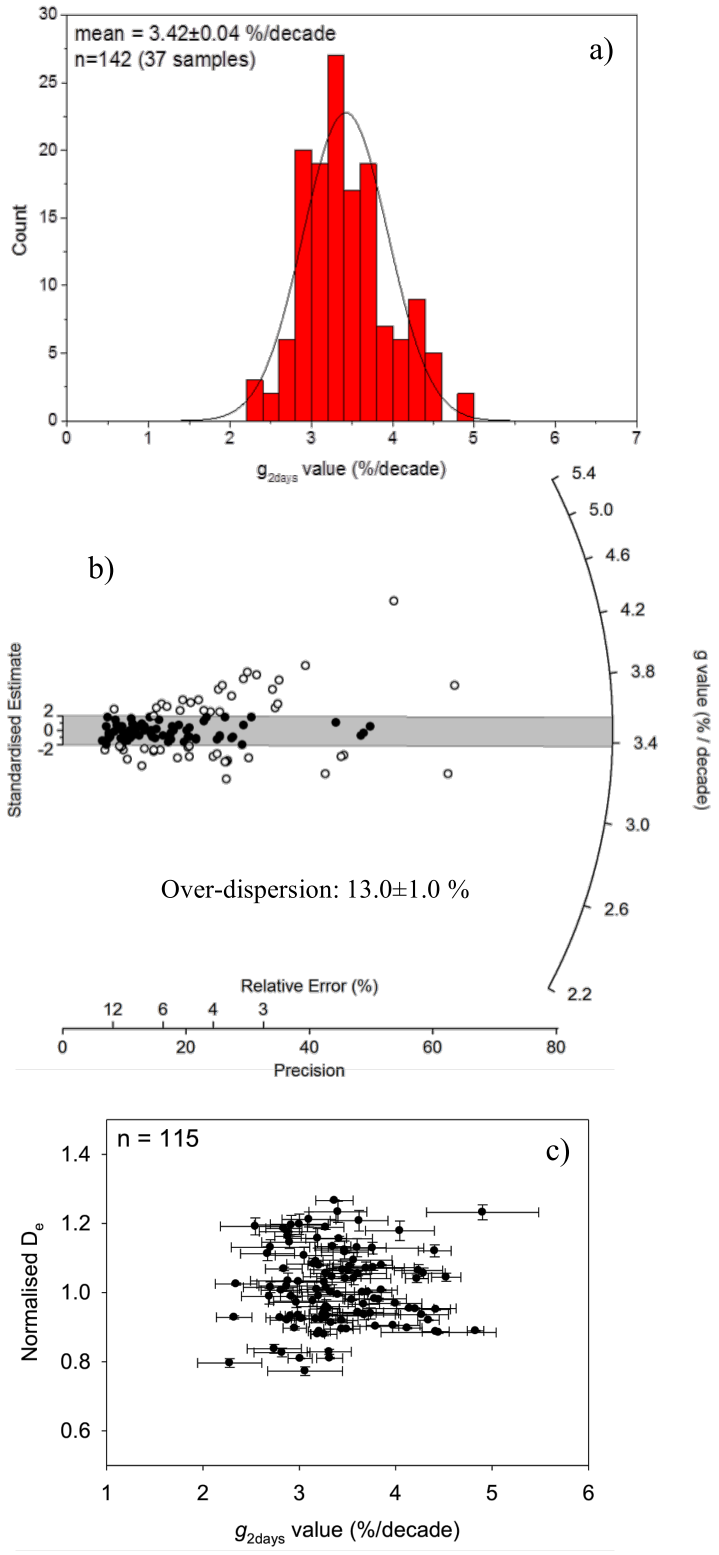

Supplementary Figure 10.
Summary of fading rate (g_{2days}) measurements. a) Histogram of all fading rate measurements with relative uncertainties ≤15%. b) Same data as in a) but shown as a radial plot. c) Equivalent dose (normalised to average D_e of each sample) as a function of fading rate.

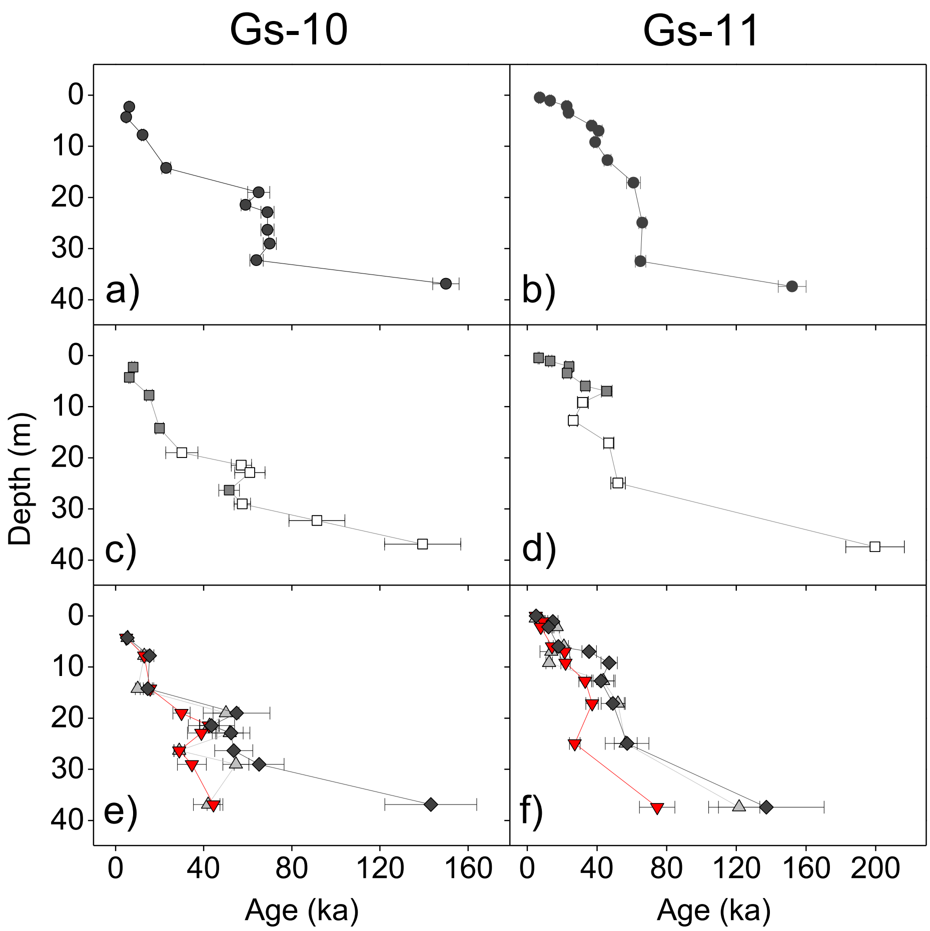

Supplementary Figure 11.

A comparison of ages derived from cores GS-10 and GS-11 using a) and b) multi-grain K-rich feldspar, c) and d) multi-grain quartz. The closed squares represent the quartz ages considered to be reliable and the open symbols represent the ages derived from sample where bounded dose estimates could not be derived for more than 15% of the otherwise accepted aliquots. e) and f) Single grain quartz. An additional uncertainty of 30% has been added prior to dose estimation. The downward facing triangles represent the ages obtained using the CAM, the upward facing triangles the most prominent dose component identified by the FMM (FMM_{prom}) and the diamonds the CAM ages derived using the two additional selection criteria.

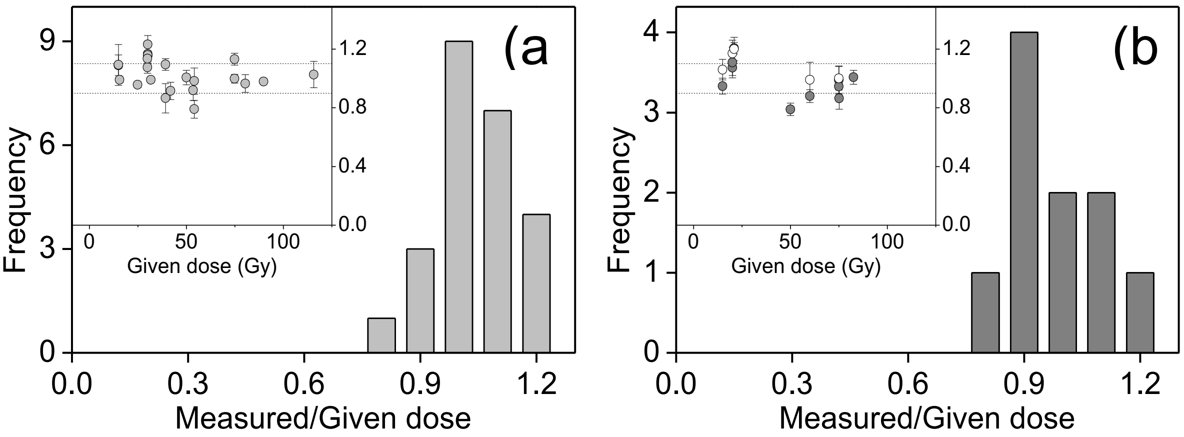

Supplementary Figure 12.

Quartz dose recovery data. (a) Quartz multi-grain beta dose recovery results for 24 samples (140 aliquots). Here the average dose recovery value is given for each sample. The average dose recovery ratio (measured/given) is 1.03±0.02 (n=24). The inset shows the dose recovery ratio as a function of given dose. (b) Quartz single-grain beta dose recovery results for 10 samples (091903, -06, 101903, -06, 111903, -04, -10, -12, -15 and -16). A total of 21,800 individual grains were measured. The average dose recovery ratio (measured/given) is 0.98±0.04 (n=10). The inset shows the dose recovery ratio as a function of given dose. The filled symbols represent the data obtained using the standard rejection criteria, whereas the open symbols represent the data obtained using the additional rejection criteria based on thermal stability and dose response curve characteristics.

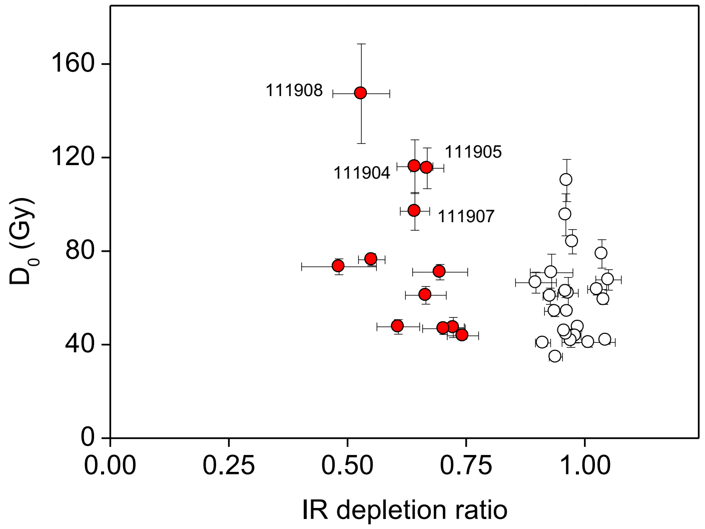

Supplementary Figure 13.

Relationship between average D_0 value and average IR depletion ratio for all multi-grain quartz samples except sample 111901 for which the IR depletion ratio was not measured explicitly.

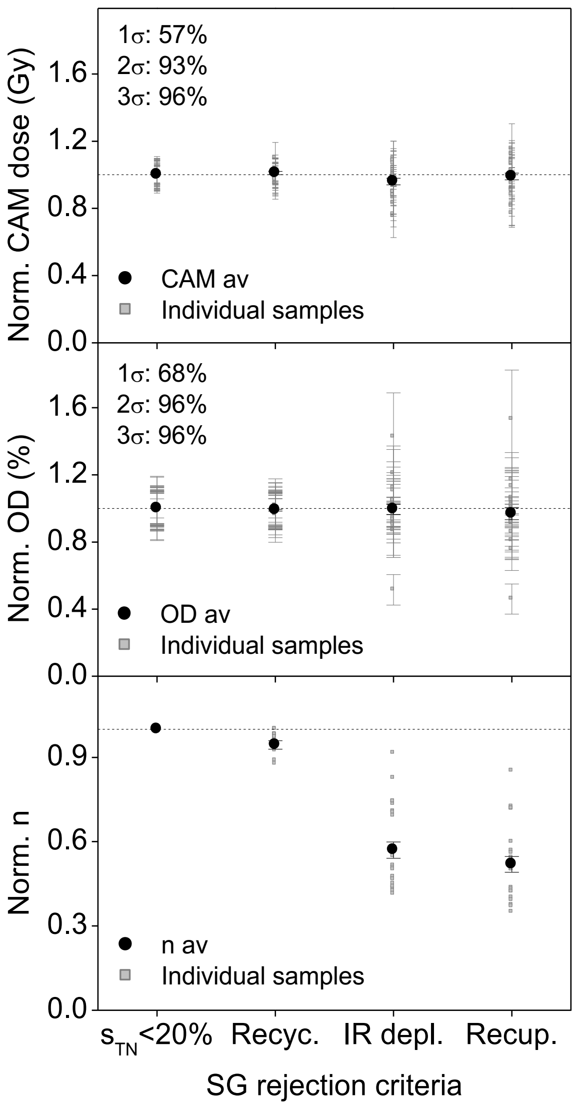

Supplementary Figure 14.

Effect of applying the various single grain (SG) rejection criteria on the normalized central dose (CAM), the normalised over-dispersion (OD) and the number of accepted dose estimates (n) for 30 natural samples (squares). The circles indicate the average values for all samples. All average values for CAM and OD are consistent with unity. "s_{TN}<20%" includes all grains with a bounded dose estimate with uncertainties on the natural test dose signal of less than 20%. "Recyc." includes all grains from "s_{TN}<20%" except the ones with recycling values not consistent with unity at 2σ. "IR depl." includes all grains from "Recyc." except the ones with IR depletion ratios not consistent with unity at 2 σ. "Recup" includes all grains from "IR depl." except the ones with recuperation doses statistically greater than 1 Gy.

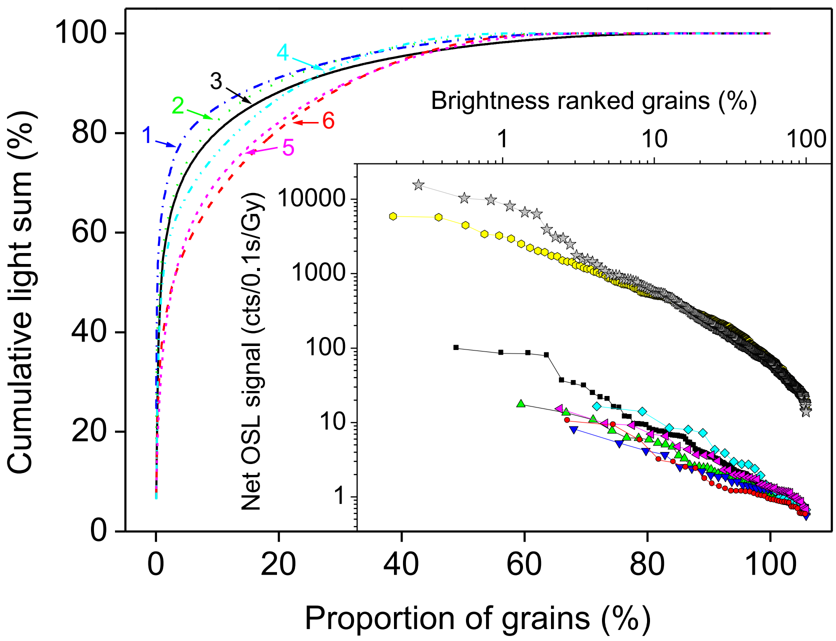

Supplementary Figure 15.

Cumulative light sums for the natural test dose signal for samples (1) 091902 (downward triangles; GS-11), (2) 111910 (upward triangles; GS-7), (3) 101901 (squares; GS-10), (4) 111913 (diamonds; GS-13), (5) 111917 (left facing triangles; GS-14) and (6) 111922 (circles; SRH-5A). The inset shows the net OSL signal per Gy from the natural test dose signal for the accepted grains ranked from the brightest to the dimmest grain for the same samples as shown in the main graph. Also shown are the results from a fluvial sample from Namibia (031304; hexagonal) and a coastal deposit from Denmark (Rømø; stars).

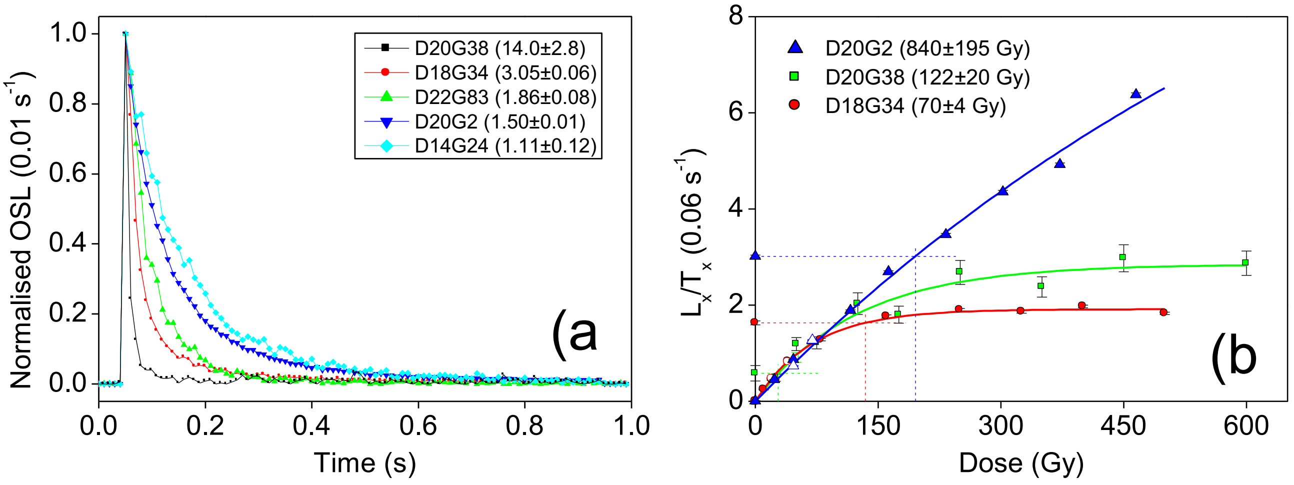

Supplementary Figure 16.

Single grain luminescence characteristics. (a) Examples of single-grain regenerated stimulation curve shapes for grains from sample 111909. The numbers given in the legend in brackets are the fast ratio F_R values. Please note that these F_R values have been obtained using different summation limits and a higher stimulation power than applied by Duller (2012) (ref. 70) and thus the F_R values cannot be directly compared. (b) Examples of dose response curves for three of the grains shown in a). The open symbols indicate the recycling points. The sensitivity-corrected natural OSL signals are also shown. All dose response curves have been fitted using a single saturating exponential function. The numbers given in brackets are the D_0 values.

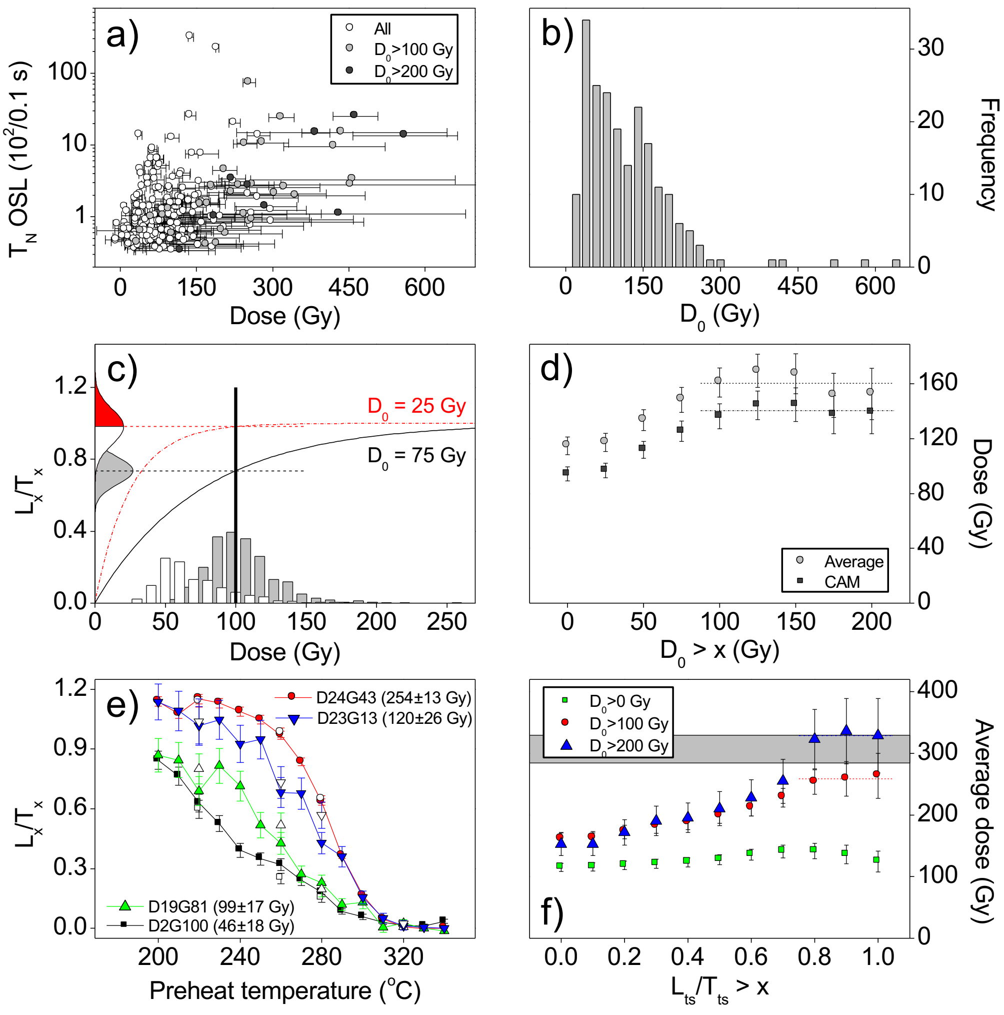

Supplementary Figure 17.

Single grain results from sample 111909. (a) The natural test dose response as a function of the dose estimated for individual grains. The light grey symbols indicate grains with a D_0 value larger than 100 Gy and an L_{ts}/T_{ts} ratio larger than 0.8, whereas the dark grey symbols indicate grains with a D_0 value larger than 200 Gy and an L_{ts}/T_{ts} ratio larger than 0.8 (see text for details). (b) Frequency histogram of D_0 values known to better than 30% for the accepted grains. (c) Theoretical relationship between L_n/T_n, dose response curves and dose estimates. The dose in the sample is set to be 100 Gy. Solid black line is a saturating exponential with a D_0 value of 75 Gy. The normal distribution centered on ~0.74 on the y-axis represents L_n/T_n measurements with an overall standard deviation of 10%. The grey shaded histogram represents the doses obtained from 1,000 random L_n/T_n values extracted from the normal distribution and the dose response curve with D_0 = 75 Gy. Also shown is a saturating exponential function with D_0 = 25 Gy (dash dotted line). The normal distribution centered on ~0.98 on the y-axis represents L_n/T_n measurements with an overall standard deviation of 10% expected from a given dose of 100 Gy. The lower part of the distribution (white) can result in dose estimates (white histogram) when interpolated onto a dose response curve with a D_0 value of 25 Gy. (d) Average dose (circles) and CAM dose (squares) as a function of D_0 greater than the value indicated on the x-axis. The dashed lines indicate the average of the last five points. (e) Pulse anneal curves. L_x/T_x ratios as a function of preheat temperature for four different grains. Open symbols indicate recycling points measured after the highest preheat temperature measurement. f) The effect of excluding grains on the basis of thermal stability on the average dose for various D_0 rejection criteria. The dashed line indicates the average of the last three measurements. The grey horizontal band indicates the quartz dose predicted from the feldspar measurements of the same sample.

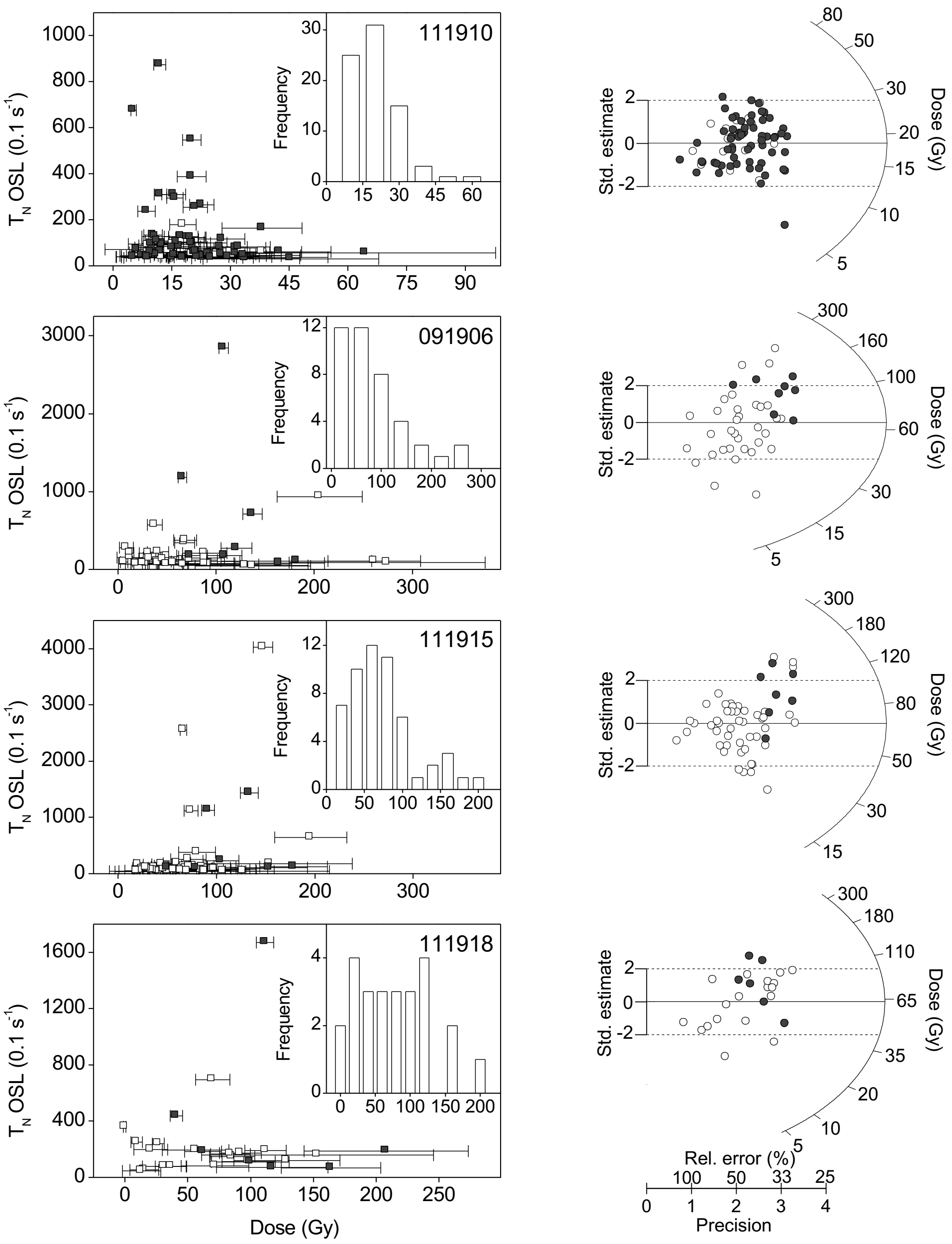

Supplementary Figure 18.
Single grain natural dose distributions. On the left the natural test dose signal is plotted as a function of the measured dose (open and closed symbols). The assigned uncertainties are based on counting statistics and curve fitting errors. The closed symbols show the grains that pass the additional rejection criteria outlined in the section *Additional single grain rejection criteria*. The insets show histograms of the same data. On the right radial plots of the same data are shown. A single negative dose estimate (-0.2±1.0 Gy) has been omitted in the radial plot for sample 111918. The assigned uncertainties have an additional uncertainty of 30% added (see text for details).

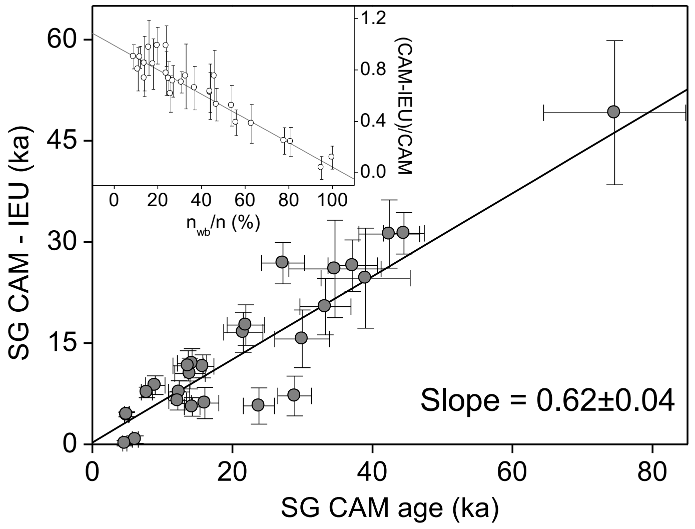

Supplementary Figure 19.

Comparison of single grain quartz ages obtained using the CAM and the IEU after assigning an additional uncertainty of 30% to the uncertainty of individual dose points. The solid line shows a linear fit to the data. The inset shows the proportion of well-bleached grains against the normalized amount of incomplete bleaching.

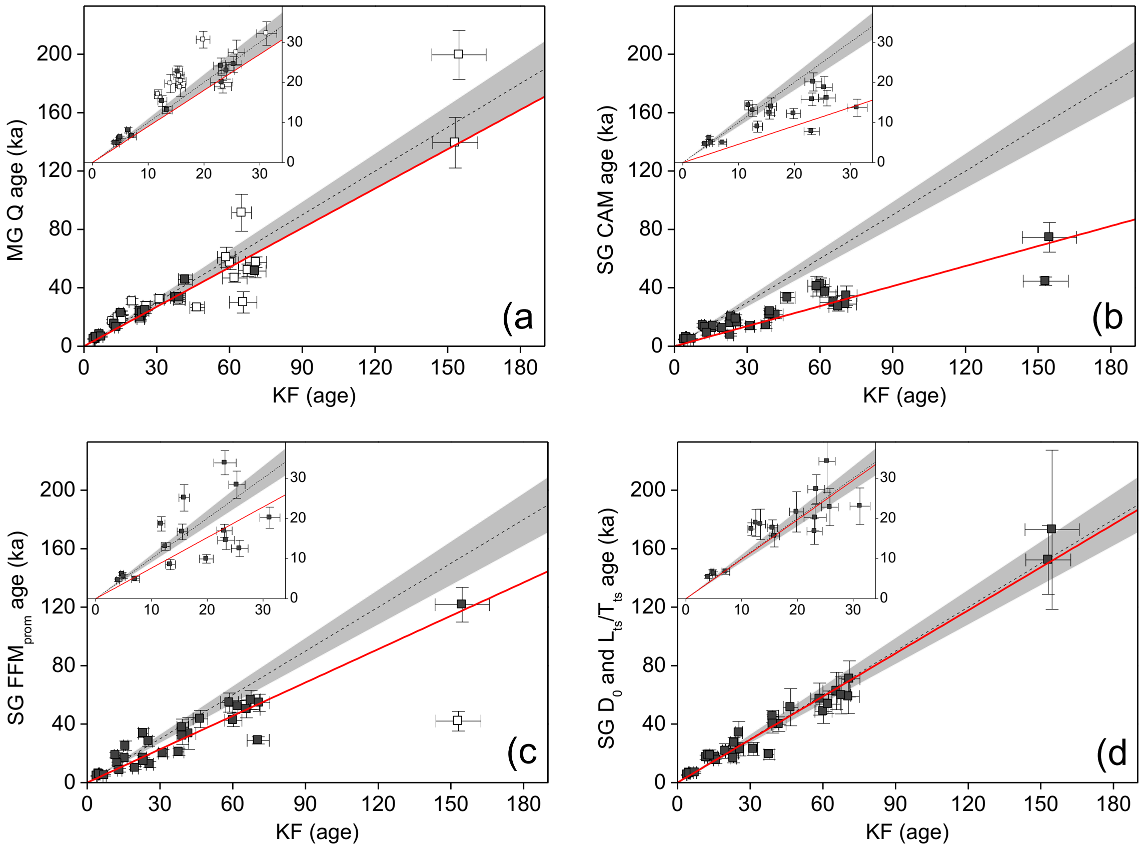

Supplementary Figure 20.

A comparison of all ages derived for multi-grain K-rich feldspar (MG KF), multi-grain quartz (MG Q), and single grain (SG) quartz. Quartz ages are shown as a function of feldspar ages. The dashed lines indicate the 1:1 line and the shaded area surrounding it indicates ± 10 %. The solid lines indicate the best linear fit to the data. The insets show the same data as the main graphs but only for feldspar ages less than ~35 ka. (a) The closed symbols represent the quartz ages with <15% unbounded dose estimates and the open symbols represent the ages derived from sample where bounded dose estimates could not be derived for >15% of the otherwise accepted aliquots. The linear fit has been obtained using all 37 data points. (b) Single grain quartz ages obtained using the CAM. (c) Single grain quartz ages obtained using the most prominent dose component identified by the FMM using an additional uncertainty of 30%. For samples 091906, 101906 and 111903 the component with the largest dose (see text for details) is shown. The linear fit omits the age for sample 111909 (open symbol). (d) Average quartz ages obtained using the two additional rejection criteria.

Name	UTM Coordinates WGS84/NAD83	Depth of drill hole (m)	Description
Near Kalibangan (Rajasthan)			
GS-7	3261617 N 412947 E	38.44	Located on the right bank (active floodplain), about 1.5 km northwest of modern Ghaggar River channel.
GS-10	3260754N 414628E	39.02	Located on the left bank (active floodplain), about 400 m southwest of modern Ghaggar River channel.
GS-11	3259899N 415029E	43.32	Located on the left bank, about 1.3 km southwest of modern Ghaggar River channel.
GS-13	3263962N 411152E	42.32	Located near the Pilibanga Polytechnic College in the Pilibanga town, on the right bank high, about 9 km northwest of the modern Ghaggar River channel.
GS-14	3265635N 409348E	36.95	Located further 2.5 km northwest of site GS-13.
Near Sirhind (Punjab)			
SRH-5	3390379N 639545E	51.4	Located near village Balari Khurd in Sirhind, Punjab at centre of the trace of the palaeochannel belt
Near Kunal (Haryana)			
KNL-1	3280343N 563528E	40.33	Located near village Chimun in Haryana; it lies at the right edge of the trace of the palaeochannel.
Near Moonak (Punjab)			
MNK-6	3297993N 589075E	36.19	Located near village Moonak; it lies at the centre of the trace of the palaeochannel belt.

Supplementary Table 1. Location of drill sites.

Supplementary Table 2. Description of sedimentary facies identified in drill cores.

FACIES	CHARACTERISTICS	INTERPRETATION
	Floodplain fines	
F1 Yellow Mud	Mix of silt and clay. Very few carbonate nodules (2–8 mm). At few horizons irregular lenses of fine sand. The beds are compact and structureless. White patches of calcareous cement. Weakly pedogenised and very few roots at some places.	Mainly floodplain deposits with weak pedogenised character developed during minor breaks in deposition.
F2 Red Brown Silty clay	Compact, tough, stiff and sticky silty clay. Sometimes mix with patches of silt and very fine sand. Fe–nodules along with very few carbonate nodules. Dark mottles.	Abundant in most of the cores near the modern Ghaggar channel. Floodplain deposits, slightly pedogenised with presence of nodules and soil organic matter.
F3 Red–Brown Very Fine Sand	Fragile and perforated very fine sand. Very fine mica grains. Carbonate nodules absent. Dark mottles. Mainly occurs in GS cores.	Floodplain deposit with no pedogenesis.
F4 Calcrete zone	Zones/Layers of subangular to subrounded carbonate nodules (5–10 mm) in the matrix of poorly sorted sand. Occurs at very thin layers of few cm in GS and MNK cores.	Deposition of calcrete mostly due to groundwater fluctuation.
	Channel deposits	
Cm1 Red Brown Silt	Reddish brown unsorted and loose silt with grain size up to very fine sand. Very fine mica grains (dominantly muscovite). Non–cohesive. No calcrete.	Mostly limited to shallow depths in GS-transects. Channel margin deposits; locally aeolian reworked.
Cm2 Yellow Brown Very Fine Sand	Fine grained micaceous sand. Yellow brown and fairly sorted grains. No inclusions and no pedogenesis.	
C1 Grey coarse Sand	Coarse-grained grey sand with salt & pepper appearance. Mica rich sand. Carbonate nodules and muddy layers only occasionally present. Mica mineral content is variable at different depths.	Most abundant facies and occurs as thick sand bodies between depths of 8–30 m. Typical channel deposit of Himalayan-sourced rivers.
C2 Grey Medium Sand	Light grey to grey, medium sand. Salt and pepper appearance. Mica rich sand. Carbonate nodules absent. Infrequent calcretised sand nodule. Organic matter or pedogenesis absent.	Mainly associated with C1 facies with minor association with C4 facies. Typical channel deposit of Himalayan sourced rivers.

C3<br>Grey Micaceous Fine–Medium Sand	Fine to medium grained grey sand. Highly micaceous. Calcrete nodules or organic matter absent. No mottling or pedogenesis.	Mostly associated with C1 and C4 facies. Lower energy channel deposit.
C4<br>Grey Fine Sand	Very fine mica grains include muscovite and biotite. Black and brown mottles. Calcrete nodules or any other inclusions absent.	C4 variably occurs till shallow depth of 20m and is mainly associated with F1 facies. C5 is not much abundant.<br>Low energy channel deposit; sub-aerial exposure for short periods and locally aeolian reworking during low discharge as suggested by brown and black mottles and lack of pedogenesis.
C5<br>Yellow–Brown Fine Sand	Fairly sorted fine sand. Very fine mica grains. Non–cohesive. Calcrete nodules absent.	
	Aeolian sand	
A1<br>Yellow Silty Sand	Homogeneous and non–cohesive, fairly well–sorted silty sand. Poor in mica relative to channel sands. It occurs at depths of about 32 to 36 meters in different cores. In top part it becomes yellow silt mixed with irregular lenses of very fine grey sand having small carbonate nodules disseminated within the lenses. Carbonate nodules are sub–rounded and elongated (0.5 to 3 cm).	Windblown sand deposition prior to onset of channel activity.

Supplementary Table 2 (continued). Description of sedimentary facies identified in drill cores.

Name	UTM Coordinates WGS84/NAD83	Description
Sutlej	3429548N 644576E	Modern sand bar at mountain exit (near Ropar town) comprising fine-medium grained sand.
Ghaggar	3398826N 679966E	Modern sand bar at mountain exit (near Panchkula town) comprising fine-medium grained sand.
Yamuna	3360005N 749602E	Modern sand bar at mountain exit (near Khara Village) comprising fine-medium grained sand.
Ganges	3321156N 230663.6E	Modern sand bar at mountain exit (near village Haripur Kalan) comprising medium grained sand.
Dune sand	3258503N 416110E	Sand from inactive dunes adjacent to Ghaggar-Hakra palaeochannel in Hanumangarh district of Rajasthan. Dunes are at northern fringe of Thar Desert.

Supplementary Table 3. Location of sand samples from modern rivers and modern dune.

Sample	Depth (m)	Lithology
		SRH–5 Core
SRH–5 Z1	5.2	Grey micaceous coarse sand. Horizon thickness ~1.5 m.
SRH–5 Z2	12.1	Grey micaceous medium sand. Horizon thickness ~2 m.
SRH–5 Z3	19.8	Grey micaceous medium sand. Horizon thickness ~2 m.
SRH–5 Z4	29.5	Grey micaceous medium–fine sand. Horizon is ~2 m thick.
SRH–5 Z5	35	Grey micaceous medium sand. Horizon thickness <8 m .
SRH–5 Z6	39.1	Grey micaceous coarse sand. Horizon thickness ~1.8 m.
		KNL–1 Core
KNL–1 Z1	11.1	Grey micaceous medium–fine sand. Horizon thickness ~5.5 m.
KNL–1 Z4	23.9	Grey micaceous fine sand. Horizon thickness ~2.5 m.
KNL–1 Z6	32.4	Grey micaceous medium–fine sand. Horizon is ~4 m thick.
		GS–11 Core
GS–11 Zr1	7.2	Grey micaceous fine sand. Horizon thickness ~3 m.
GS–11 Zr8	9.0	Grey micaceous medium–fine sand. Horizon is ~1 m thick.
GS–11 Zr2	15.4	Grey micaceous fine sand. Horizon thickness ~8 m.
GS–11 Zr9	19.6	Grey micaceous fine sand. Horizon thickness ~8 m.
GS–11 Zr3	23.5	Grey micaceous medium sand. Horizon thickness ~2 m.
GS–11 Zr10a	32.4	Grey micaceous coarse sand. Horizon thickness ~4 m.
GS–11 Zr5	33.9	Grey micaceous fine sand. Horizon thickness ~3 m.
GS–11 Zr6	40.3	Yellow silty aeolian sand.
		GS–10 Core
GS–10 Zr8	8.2	Red–brown very fine sand. Horizon thickness ~2 m.
GS–10 Zr3	11.4	Grey micaceous fine sand. Horizon thickness ~3.5 m.
GS–10 Zr2	17.9	Grey micaceous medium–fine sand. Horizon is ~5 m thick.
GS–10 Zr1	20.3	Grey micaceous medium–fine sand. Horizon is ~5 m thick.
GS–10 Zr4a	23.6	Grey micaceous coarse sand. Horizon thickness ~2 m.
GS–10 Zr5a	26.7	Grey micaceous medium sand. Horizon thickness ~4 m.
		GS–7 Core
GS–7 Zr1	3.1	Light brown very fine sand. Horizon thickness ~1 m.
GS–7 Zr2	5.5	Grey micaceous fine sand. Horizon thickness ~4 m.
GS–7 Zr3	9.6	Grey micaceous fine sand. Horizon thickness ~3 m.
		Modern Sands
Sutlej River	–	Medium–fine sand collect from modern sand bar.
Ghaggar River	–	Medium–fine sand collect from modern sand bar.
Yamuna River	–	Medium–fine sand collect from modern sand bar.
Ganges River	–	Medium sand collect from modern sand bar.
Thar Desert dune sand	–	Sand from inactive dunes near the Ghaggar river.

Supplementary Table 4. Detrital zircon samples. Details of sediment samples collected from different drill cores, and modern river sands, for U–Pb detrital zircon single grain dating.

Sample	Depth (m)	Lithology
		SRH–5 Core
SRH–5 Z1	5.2	Grey micaceous coarse sand. Horizon thickness ~1.5 m.
SRH–5 Z2	12.1	Grey micaceous medium sand. Horizon thickness ~2 m.
		GS–11 Core
GS–11 Z1M	7.2	Grey micaceous fine sand. Horizon thickness ~3 m.
GS–11 Z8M	9.0	Grey micaceous medium–fine sand. This highly mica rich horizon is ~1 m thick.
GS–11 Z2M	15.4	Grey micaceous fine sand. Horizon thickness ~8 m with presence of few mud layers.
GS–11 Z9M	19.6	Grey micaceous fine sand. Horizon thickness ~8 m with presence of few mud layers.
GS–11 Z3M	23.5	Grey micaceous medium sand. Horizon thickness ~2 m.
GS-11 Z4M	26.4	Grey micaceous medium sand. Horizon thickness ~0.5 m.
GS–11 Z10M	32.4	Grey micaceous coarse sand. Horizon thickness ~4 m.
GS–11 Z5M	33.9	Grey micaceous fine sand. Horizon thickness ~3 m.
		GS–10 Core
GS–10 XR3	11.4	Grey micaceous fine sand. Horizon thickness ~3.5 m with a calcrete zone below.
GS–10 XR2	17.9	Grey micaceous medium–fine sand. This highly mica rich horizon is ~5 m thick.
GS–10 XR1	20.3	Grey micaceous medium–fine sand. This highly mica rich horizon is ~5 m thick.
		Modern river samples
Yamuna River sand		Medium–fine sand collected from modern sand bar.
Sutlej River sand		Medium–fine sand collected from modern sand bar.

Supplementary Table 5. Details of detrital mica samples for Ar-Ar dating.

Samples		Depth (m)	Lithology
Field Code	Risø Lab Code		
		Core GS–11	
OSL–11–1–0.0	101903	0.5	Red brown silt
OSL–11–2–1.1	101904	1.1	Mud/silty sand
OSL–11–3–2.16	091901	2.2	Silty fine sand
OSL–11–4–3.47	101905	3.5	Grey fine sand
OSL–11–6–5.97	091902	6.0	Grey fine sand
OSL–11–7–6.97	091906	7.0	Grey fine sand,
OSL–11–9–9.19	101906	9.2	Grey medium–fine sand
OSL–11–12–12.72	091903	12.7	Grey medium sand
OSL–11–16–17.12	091907	17.1	Grey fine-medium sand
OSL–11–23–25.95	091904	26.0	Grey coarse sand
OSL-11-30-32.6	131913	32.6	Grey coarse sand
OSL–11–34–37.42	091905	37.4	Aeolian yellow brown sand
		Core GS–10	
OSL–10–4–2.29	111901	2.3	Red brown very fine sand
OSL–10–7–4.31	101901	4.3	Red brown very fine sand
OSL–10–11–7.8	111902	7.8	Red brown very fine sand
OSL–10–21–14.24	111903	14.2	Grey fine sand
OSL–10–26–18.99	111904	19.0	Grey medium–fine sand
OSL–10–29–21.46	101902	21.5	Grey medium sand
OSL–10–31–22.89	111905	22.9	Grey coarse sand
OSL–10–34–26.34	111906	26.3	Grey medium–coarse sand
OSL–10–37–29.04	111907	29.0	Grey Coarse sand
OSL–10–40–32.26	111908	32.3	Grey coarse sand
OSL–10–44–36.89	111909	36.9	Aeolian yellow brown sand
		Core GS–7	
OSL-7-4-2.34	111910	2.3	Yellow brown very fine sand
OSL-7-5-3.71	111911	3.7	Yellow brown very fine sand
OSL-7-6-5.10	111912	5.1	Yellow brown very fine sand

Supplementary Table 6. Details of optically stimulated luminescence samples.

		Core GS–13	
OSL-13-4-2.45	111913	2.5	Grey fine sand
OSL-13-6-5.07	111914	5.0	Grey fine sand
OSL-13-8-7.16	111915	7.2	Grey fine sand
		Core GS–14	
OSL-14-1-0.4	121906	0.4	Yellow sandy silt
OSL-14-1-1.5	121907	1.5	Yellow sandy silt
OSL-14-4-2.10	111916	2.1	Yellow sandy silt
OSL-14-6-4.12	111917	4.1	Yellow brown very fine sand
OSL-14-8-5.85	111918	5.9	Grey fine sand
		Core SRH-5	
SRH-5A-1-2.35	111919	2.4	Yellow mud/silt
SRH-5A-1-3.05	111920	3.0	Yellow mud/silt
SRH-5A-2-3.55	111921	3.6	Yellow mud/silt
SRH-5A-3-4.29	111922	4.3	Grey medium sand
SRH-5A-7-8.42	111923	8.4	Grey fine sand
SRH-5A-8-9.27	111924	9.3	Grey medium sand
SRH-5-16-20.7	131901	22	Grey medium sand
SRH-5-21-30.4	131902	31	Grey medium sand
SRH-5-27-43.2	131903	44	Grey coarse sand
		Core MNK–6	
MNK-6-6-3.9	131907	4.5	Yellow brown very fine sand
MNK-6-7-5.9	131904	6.5	Grey fine sand
MNK-6-11-11	131908	12.5	Grey fine sand
MNK-6-12-12.9	131909	14.5	Grey coarse sand
MNK-6-14-16.9	131910	17.5	Grey fine sand
MNK-6-17-21.6	131905	21.7	Grey fine sand
MNK-6-19-25.5	131911	26.2	Grey medium sand
MNK-6-23-32.2	131912	33.5	Grey medium sand
MNK-6-24-34.2	131906	35.2	Aeolian yellow brown sand

Supplementary Table 6 (continued). Details of optically stimulated luminescence samples.

Core	Sample	Grain size (µm)			w.c.	Radionuclide concentrations (Bq kg^{-1})				Dose rates (Gy ka^{-1})				
		KF	MG Q	SG Q	(%)	^{238}U	^{226}Ra	^{232}Th	^{40}K	Internal	Dry gamma	Dry beta	Total quartz	Total feldspar
	Modern	180-250	180-250	180-250	25†	20 ± 11	27.4 ± 0.9	43.1 ± 1.1	487 ± 14	0.81	1.11 ± 0.03	1.70 ± 0.06	2.40 ± 0.10	3.20 ± 0.10
	111919	180-250	180-250	180-250	31	54 ± 6	53.8 ± 0.7	74.2 ± 0.9	591 ± 13	0.81	1.77 ± 0.05	2.62 ± 0.05	3.19 ± 0.12	3.99 ± 0.12
	111920	180-250	180-250	-	35	50 ± 9	53.9 ± 0.9	75.7 ± 0.1	665 ± 14	0.81	1.84 ± 0.05	2.80 ± 0.06	3.25 ± 0.12	4.07 ± 0.12
SRH-5A	**111921**	**180-250**	**180-250**	**180-250**	**27**	**35 ± 7**	**46.7 ± 0.7**	**67.6 ± 0.8**	**624 ± 12**	**0.81**	**1.66 ± 0.04**	**2.53 ± 0.05**	**3.14 ± 0.12**	**3.96 ± 0.13**
	**111922**	**180-250**	**180-250**	**180-250**	**32**	**31 ± 6**	**38.6 ± 0.6**	**50.5 ± 0.8**	**667 ± 14**	**0.81**	**1.42 ± 0.03**	**2.46 ± 0.05**	**2.79 ± 0.10**	**3.62 ± 0.11**
	111923	180-250	180-250	-	35	27 ± 7	36.8 ± 0.8	55.4 ± 0.9	595 ± 15	0.81	1.41 ± 0.03	2.28 ± 0.05	2.56 ± 0.10	3.44 ± 0.10
	111924	180-250	180-250	-	38	31 ± 11	45.0 ± 0.9	55.7 ± 1.1	621 ± 16	0.81	1.49 ± 0.04	2.41 ± 0.07	2.63 ± 0.10	3.51 ± 0.11
	131901	180-250	-	-	*30*	25 ± 9	31.1 ± 0.8	46.7 ± 1.0	613 ± 15	0.81	1.25 ± 0.03	2.15 ± 0.05	-	3.24 ± 0.10
	131902	180-250	-	-	*30*	23 ± 16	42.6 ± 1.3	78.8 ± 1.7	562 ± 19	0.81	1.67 ± 0.04	2.29 ± 0.08	-	3.62 ± 0.12
	131903	180-250	-	-	*30*	31 ± 10	33.4 ± 0.9	53.7 ± 1.1	629 ± 15	0.81	1.36 ± 0.03	2.27 ± 0.06	-	3.38 ± 0.11
	**131907**	**90-250**	**-**	**-**	***30***	**54 ± 24**	**49.7 ± 2.0**	**79.8 ± 2.1**	**543 ± 30**	**0.67**	**1.72 ± 0.05**	**2.43 ± 0.12**	**-**	**3.73 ± 0.14**
	**131904**	**180-250**	**-**	**-**	***30***	**33 ± 13**	**27.0 ± 1.1**	**44.0 ± 1.3**	**372 ± 15**	**0.81**	**1.00 ± 0.03**	**1.53 ± 0.07**	**-**	**2.72 ± 0.09**
	131908	90-250	-	-	*30*	27 ± 15	33.6 ± 1.3	54.2 ± 1.5	390 ± 17	0.67	1.18 ± 0.03	1.66 ± 0.08	-	2.90 ± 0.10
	131909	180-250	-	-	*30*	23 ± 15	23.9 ± 1.2	34.9 ± 1.4	505 ± 22	0.81	0.98 ± 0.03	1.75 ± 0.08	-	2.79 ± 0.10
MNK-6	131910	90-250	-	-	*30*	64 ± 18	54.2 ± 1.6	80.9 ± 1.5	551 ± 22	0.67	1.77 ± 0.05	2.53 ± 0.09	-	3.75 ± 0.13
	131905	90-180	-	-	*30*	37 ± 14	41.1 ± 1.2	58.3 ± 1.4	716 ± 19	0.53	1.54 ± 0.04	2.59 ± 0.08	-	3.51 ± 0.12
	131911	180-250	-	-	*30*	27 ± 10	27.1 ± 0.8	36.4 ± 1.0	570 ± 17	0.81	1.07 ± 0.03	1.95 ± 0.06	-	2.97 ± 0.10
	131912	180-250	-	-	*30*	36 ± 10	39.8 ± 1.2	56.5 ± 1.4	990 ± 24	0.81	1.72 ± 0.04	3.26 ± 0.08	-	4.29 ± 0.14
	131906	90-250	-	-	*30*	71 ± 15	16.0 ± 1.3	68.2 ± 1.5	564 ± 15	0.67	1.57 ± 0.04	2.46 ± 0.08	-	3.54 ± 0.12
	111910	180-250	90-180	150-180	31	68 ± 9	47.9 ± 0.8	68.7 ± 1.0	610 ± 13	0.81	1.68 ± 0.04	2.66 ± 0.06	3.00 ± 0.11	3.82 ± 0.12
Gs-7	111911	180-250	150-180	-	30	29 ± 7	42.1 ± 0.6	65.1 ± 0.8	484 ± 10	0.81	1.48 ± 0.04	2.10 ± 0.04	2.97 ± 0.11	3.78 ± 0.12
	111912	180-250	150-250	180-250	25	64 ± 5	59.4 ± 0.7	92.0 ± 0.9	604 ± 11	0.81	2.04 ± 0.05	2.85 ± 0.05	3.03 ± 0.12	3.83 ± 0.12
	111901	125-180	63-300	-	32	34 ± 7	39.3 ± 0.7	60.5 ± 0.8	560 ± 11	0.58	1.47 ± 0.04	2.27 ± 0.05	2.72 ± 0.10	3.34 ± 0.10
	101901	90-180	90-180	150-180	27	40 ± 6	51.1 ± 0.7	71.3 ± 0.8	764 ± 14	0.53	1.85 ± 0.05	2.97 ± 0.05	3.56 ± 0.14	4.16 ± 0.14
	**111902**	**125-180**	**180-250**	**180-250**	**29**	**45 ± 10**	**48.0 ± 0.9**	**75.4 ± 1.1**	**462 ± 12**	**0.58**	**1.64 ± 0.04**	**2.22 ± 0.06**	**2.81 ± 0.11**	**3.43 ± 0.12**
	**111903**	**180-250**	**180-250**	**180-250**	**39**	**33 ± 7**	**35.5 ± 0.6**	**58.6 ± 0.8**	**475 ± 10**	**0.81**	**1.36 ± 0.03**	**2.01 ± 0.04**	**2.25 ± 0.08**	**3.06 ± 0.09**
	111904	180-250	180-250	180-250	30	32 ± 8	33.9 ± 0.7	48.6 ± 0.8	603 ± 13	0.81	1.32 ± 0.03	2.26 ± 0.05	2.53 ± 0.10	3.34 ± 0.11
Gs-10	101902	180-250	180-250	180-250	34	32 ± 5	31.1 ± 0.5	47.3 ± 0.6	552 ± 11	0.81	1.24 ± 0.03	2.10 ± 0.04	2.30 ± 0.09	3.11 ± 0.10
	111905	180-250	180-250	180-250	28	21 ± 5	27.7 ± 0.4	36.4 ± 0.6	570 ± 11	0.81	1.07 ± 0.03	1.80 ± 0.04	2.17 ± 0.09	2.98 ± 0.09
	111906	180-250	180-250	180-250	30	18 ± 6	26.2 ± 0.5	36.0 ± 0.6	619 ± 12	0.81	1.12 ± 0.02	2.11 ± 0.04	2.28 ± 0.09	3.09 ± 0.10
	111907	180-250	180-250	180-250	27	26 ± 6	31.9 ± 0.6	47.2 ± 0.7	495 ± 10	0.81	1.20 ± 0.03	1.93 ± 0.04	2.27 ± 0.09	3.08 ± 0.10
	111908	180-250	180-250	-	22	25 ± 7	26.7 ± 0.6	34.9 ± 0.7	611 ± 13	0.81	1.10 ± 0.03	2.11 ± 0.05	2.42 ± 0.10	3.23 ± 0.11
	111909	180-250	180-250	180-250	31	23 ± 6	30.5 ± 0.6	50.5 ± 0.7	456 ± 10	0.81	1.20 ± 0.03	1.83 ± 0.04	2.12 ± 0.08	2.93 ± 0.09
	101903	180-250	90-250	180-250	26	49 ± 7	45.9 ± 0.7	61.2 ± 0.9	648 ± 13	0.81	1.60 ± 0.04	2.61 ± 0.05	3.32 ± 0.12	4.13 ± 0.13
	101904	90-180	90-250	180-250	36	69 ± 11	64.3 ± 1.0	71.9 ± 1.1	899 ± 19	0.53	2.06 ± 0.06	3.53 ± 0.08	3.87 ± 0.14	4.48 ± 0.15
	**091901**	**45-180**	**90-180**	**125-180**	**29**	**56 ± 5**	**53.6 ± 0.6**	**81.7 ± 0.8**	**629 ± 11**	**0.43**	**1.89 ± 0.05**	**2.78 ± 0.05**	**3.45 ± 0.12**	**3.96 ± 0.13**
	**101905**	**180-250**	**90-250**	**-**	**35**	**41 ± 5**	**43.8 ± 0.5**	**65.3 ± 0.7**	**592 ± 10**	**0.81**	**1.59 ± 0.04**	**2.45 ± 0.04**	**2.83 ± 0.10**	**3.64 ± 0.11**
	091902	90-180	90-180	125-180	37	36 ± 6	42.3 ± 0.7	62.8 ± 0.8	619 ± 14	0.53	1.56 ± 0.04	2.47 ± 0.05	2.77 ± 0.09	3.35 ± 0.10
Gs-11	091906	90-180	180-250	180-250	32	47 ± 5	45.5 ± 0.6	71.3 ± 0.8	598 ± 11	0.81	1.68 ± 0.04	2.54 ± 0.04	3.00 ± 0.11	3.58 ± 0.11
	101906	180-250	180-250	180-250	33	53 ± 9	41.0 ± 0.8	57.5 ± 0.9	571 ± 13	0.81	1.46 ± 0.04	2.37 ± 0.06	2.67 ± 0.10	3.48 ± 0.11
	091903	180-250	180-250	180-250	31	30 ± 8	42.2 ± 0.8	62.5 ± 0.9	529 ± 12	0.53	1.49 ± 0.04	2.20 ± 0.05	2.64 ± 0.10	3.45 ± 0.11
	091907	180-250	180-250	180-250	33	39 ± 9	41.9 ± 0.8	66.4 ± 1.0	548 ± 13	0.53	1.55 ± 0.04	2.32 ± 0.06	2.68 ± 0.10	3.49 ± 0.11
	091904	180-250	180-250	180-250	36	26 ± 6	27.4 ± 0.5	42.1 ± 0.9	514 ± 10	0.81	1.12 ± 0.03	1.92 ± 0.04	2.05 ± 0.07	2.86 ± 0.08
	131913	180-250	-	-	*30*	45 ± 9	42.4 ± 0.8	65.1 ± 0.8	636 ± 13	0.81	1.57 ± 0.04	2.48 ± 0.05	-	3.67 ± 0.12
	091905	180-250	90-180	90-180	24	28 ± 6	25.8 ± 0.6	40.4 ± 0.6	413 ± 11	0.81	1.01 ± 0.02	1.64 ± 0.04	1.48 ± 0.06	2.55 ± 0.08
	**111913**	**90-180**	**125-250**	**180-250**	**22**	**79 ± 7**	**57.3 ± 0.7**	**85.5 ± 0.9**	**640 ± 12**	**0.53**	**1.98 ± 0.05**	**2.96 ± 0.05**	**3.86 ± 0.15**	**4.46 ± 0.16**
Gs-13	111914	125-180	150-250	180-250	29	61 ± 6	56.6 ± 0.7	68.2 ± 0.8	732 ± 13	0.58	1.83 ± 0.05	2.99 ± 0.05	3.49 ± 0.13	4.13 ± 0.14
	111915	90-180	150-250	180-250	28	28 ± 7	33.4 ± 0.7	46.5 ± 0.8	698 ± 17	0.53	1.36 ± 0.03	2.48 ± 0.06	2.81 ± 0.11	3.40 ± 0.11
	121906	90-180	-	-	26	36 ± 8	48.3 ± 0.8	64.4 ± 1.0	632 ± 13	0.53	1.64 ± 0.04	2.55 ± 0.06	-	3.83 ± 0.13
	121907	90-180	-	-	48	48 ± 13	50.5 ± 1.2	76.7 ± 1.4	606 ± 18	0.53	1.79 ± 0.05	2.63 ± 0.08	-	3.42 ± 0.10
Gs-14	**111916**	**90-180**	**150-250**	**180-250**	**22**	**52 ± 14**	**48.8 ± 1.2**	**72.7 ± 1.4**	**570 ± 17**	**0.53**	**1.70 ± 0.05**	**2.52 ± 0.08**	**3.31 ± 0.14**	**3.90 ± 0.14**
	**111917**	**90-180**	**180-250**	**180-250**	**29**	**58 ± 13**	**57.5 ± 1.2**	**76.0 ± 1.4**	**758 ± 21**	**0.53**	**1.95 ± 0.05**	**3.11 ± 0.08**	**3.66 ± 0.14**	**4.26 ± 0.15**
	111918	90-180	180-250	180-250	25	33 ± 10	51.0 ± 0.9	77.2 ± 1.2	637 ± 15	0.53	1.81 ± 0.05	2.65 ± 0.06	3.37 ± 0.13	3.96 ± 0.14

Table 1 † assumed value

Supplementary Table 7.

Summary of the grain size, estimated water content (w.c.), and radionuclide concentrations used to calculate the total quartz and feldspar dose rates given in the last two columns. "KF" is potassium feldspar, "MG Q" is multi-grain quartz and "SG Q" single-grain quartz. Uncertainties represent one standard error. †assumed value. A water content of 30% is used for the 1319 samples based on the mean saturation water content of all other samples in this study. The transition between fine- and medium-grained sand and very fine-grained facies lies between the two entries in bold in each core, except for core GS-13.

Core	Sample	Depth (cm)	Multi-grain K-feldspar				Multi-grain quartz				
			Dose (Gy)	KF_{uncor} age (ka)	KF age (ka)	n	Mode	In sat (%)	Dose (Gy)	MG Q age (ka)	n
Modern	121915	20	2.2 ± 0.3	0.69 ± 0.11	0.9 ± 0.2	10	CW	0%	0.9 ± 0.2	0.38 ± 0.07	24
	111919	235	33.8 ± 0.7	8.5 ± 0.3	11.6 ± 0.4	9	POSL	54%	54 ± 2.9	17.0 ± 1.2	22
	111920	305	41 ± 2	10.1 ± 0.6	13.8 ± 0.8	6	POSL	23%	64 ± 7.1	19.7 ± 2.3	17
	**111921**	**355**	**44.1 ± 0.9**	**11.1 ± 0.4**	**15.3 ± 0.6**	**6**	**POSL**	**27%**	**68 ± 7.1**	**21.6 ± 2.5**	**8**
	**111922**	**429**	**41.2 ± 1.0**	**11.4 ± 0.4**	**15.6 ± 0.6**	**12**	**POSL**	**25%**	**57 ± 4.0**	**20.3 ± 1.6**	**15**
SRH-5A	111923	842	37.6 ± 0.9	10.9 ± 0.4	15.0 ± 0.6	18	POSL	5%	58 ± 2.9	22.6 ± 1.5	37
	111924	927	39.7 ± 0.9	11.3 ± 0.4	15.5 ± 0.6	15	POSL	15%	49 ± 5.8	18.7 ± 2.3	17
	131901	2200	72 ± 2	22.2 ± 0.9	30.8 ± 1.3	12	-	-	-	-	-
	131902	3100	100 ± 3	27.6 ± 1.2	38 ± 2	11	-	-	-	-	-
	131903	4400	163 ± 4	48 ± 2	67 ± 3	12	-	-	-	-	-
	**131907**	**450**	**25 ± 3**	**6.7 ± 0.8**	**9.1 ± 1.2**	**12**	**-**	**-**	**-**	**-**	**-**
	**131904**	**650**	**16 ± 1**	**5.9 ± 0.4**	**8.0 ± 0.6**	**11**	**-**	**-**	**-**	**-**	**-**
	131908	1250	18 ± 1	6.2 ± 0.4	8.5 ± 0.6	12	-	-	-	-	-
	131909	1450	19 ± 2	6.8 ± 0.8	9.3 ± 1.0	12	-	-	-	-	-
MNK-6	131910	1750	172 ± 5	46 ± 2	64 ± 3	12	-	-	-	-	-
	131905	2170	176 ± 6	50 ± 2	70 ± 3	12	-	-	-	-	-
	131911	2620	173 ± 5	58 ± 3	82 ± 4	12	-	-	-	-	-
	131912	3350	265 ± 10	62 ± 3	86 ± 4	9	-	-	-	-	-
	131906	3520	242 ± 11	68 ± 4	96 ± 5	12	-	-	-	-	-
	111910	234	13.3 ± 0.3	3.48 ± 0.14	4.7 ± 0.2	12	CW	0%	18.0 ± 0.7	6.0 ± 0.4	41
GS-7	111911	371	12.6 ± 0.2	3.33 ± 0.12	4.5 ± 0.2	12	CW	3%	14.3 ± 0.4	4.8 ± 0.2	38
	111912	510	11.3 ± 0.3	2.94 ± 0.12	4.0 ± 0.2	11	CW	0%	14.9 ± 0.6	4.9 ± 0.3	48
	111901	229	15.6 ± 0.4	4.67 ± 0.18	6.3 ± 0.3	18	CW	0%	21.8 ± 1.5	8.0 ± 0.6	14
	101901	431	15.2 ± 0.3	3.66 ± 0.14	4.9 ± 0.2	19	CW	0%	22.4 ± 0.6	6.3 ± 0.3	48
	**111902**	**780**	**30.9 ± 1.0**	**9.0 ± 0.4**	**12.3 ± 0.6**	**15**	**CW**	**0%**	**43 ± 3**	**15.3 ± 1.4**	**23**
	**111903**	**1424**	**51 ± 3**	**16.5 ± 1.2**	**22.8 ± 1.7**	**9**	**CW**	**6%**	**45 ± 3**	**20 ± 2**	**16**
	111904	1899	155 ± 10	46 ± 3	65 ± 5	6	POSL	83%	76 ± 18	30 ± 7	4
GS-10	101902	2146	132 ± 3	42.5 ± 1.5	59 ± 2	12	CW	31%	131 ± 9	57 ± 5	27
	111905	2289	147 ± 4	49 ± 2	69 ± 3	6	POSL	68%	132 ± 14	61 ± 7	9
	111906	2634	153 ± 5	50 ± 2	69 ± 3	6	CW	8%	118 ± 10	52 ± 5	22
	111907	2904	154 ± 4	50 ± 2	70 ± 3	6	CW	33%	131 ± 7	58 ± 4	32
	111908	3226	148 ± 4	46 ± 2	64 ± 3	9	CW	22%	221 ± 29	91 ± 13	14
	111909	3689	314 ± 7	107 ± 4	150 ± 6	9	CW	73%	295 ± 35	139 ± 18	7
	101903	50	21.4 ± 1.7	5.2 ± 0.5	7.1 ± 0.6	17	CW	0%	21.8 ± 0.6	6.6 ± 0.3	38
	101904	110	43.2 ± 1.5	9.6 ± 0.5	13.2 ± 0.6	11	CW	7%	51 ± 3	13.1 ± 1.0	42
	**091901**	**216**	**65.2 ± 1.5**	**16.5 ± 0.7**	**22.7 ± 0.9**	**12**	**CW**	**0%**	**83 ± 6**	**24 ± 2**	**30**
	**101905**	**347**	**62 ± 2**	**17.1 ± 0.7**	**23.7 ± 1.0**	**15**	**CW**	**13%**	**65 ± 5**	**23 ± 2**	**20**
	091902	597	89.8 ± 1.1	26.8 ± 0.9	37.2 ± 1.2	17	CW	13%	93 ± 6	33 ± 2	42
GS-11	091906	697	106 ± 5	29.7 ± 1.6	41 ± 2	12	CW	10%	137 ± 8	46 ± 3	27
	101906	919	97 ± 2	27.9 ± 1.1	38.7 ± 1.5	18	CW	57%	85 ± 8	32 ± 3	12
	091903	1272	114 ± 4	33.0 ± 1.5	46 ± 2	15	CW	32%	70 ± 5	26 ± 2	32
	091907	1712	153 ± 9	43.9 ± 2.9	61 ± 4	12	CW	47%	125 ± 6	47 ± 3	19
	091904	2495	136 ± 3	47.6 ± 1.7	66 ± 2	12	CW	16%	107 ± 8	52 ± 4	43
	131913	3246	172 ± 5	46.9 ± 2.1	65 ± 3	11	-	-	-	-	-
	091905	3742	275 ± 12	107.8 ± 5.9	152 ± 8	11	CW	68%	295 ± 22	200 ± 17	13
	**111913**	**245**	**82.2 ± 1.1**	**18.4 ± 0.7**	**25.4 ± 1.0**	**9**	**POSL**	**50%**	**106 ± 11**	**27 ± 3**	**8**
GS-13	111914	507	91.8 ± 1.3	22.2 ± 0.8	30.7 ± 1.1	9	CW	17%	112 ± 9	32 ± 3	20
	111915	716	95 ± 2	27.9 ± 1.1	38.7 ± 1.5	6	CW	8%	94 ± 7	33 ± 3	24
	121906	40	9.4 ± 0.6	2.46 ± 0.17	3.3 ± 0.2	8	-	-	-	-	-
	121907	150	45.0 ± 1.1	13.2 ± 0.5	18.1 ± 0.7	9	-	-	-	-	-
GS-14	**111916**	**210**	**55.4 ± 1.3**	**14.2 ± 0.6**	**19.5 ± 0.8**	**12**	**CW**	**28%**	**101 ± 5**	**31 ± 2**	**28**
	**111917**	**412**	**71 ± 2**	**16.7 ± 0.8**	**23.0 ± 1.1**	**12**	**CW**	**18%**	**69 ± 5**	**19 ± 2**	**32**
	111918	585	71.5 ± 0.6	18.1 ± 0.7	24.9 ± 0.9	15	CW	7%	82 ± 6	24 ± 2	28

Supplementary Table 8.

Multi-grain (MG) dose rates and ages for K-feldspar (2 mm) and quartz (8 mm). The transitions between very fine-grained samples and fine- medium-grained samples in each core have been highlighted in bold, except in GS13. For feldspar the "KFuncor age" is the age obtained with no correction for anomalous fading, and "KF age" has been corrected for anomalous fading using an average g_{2days}-value of 3.42±0.04 % per decade (n=142). All quartz samples were measured in continuous wave (CW) mode, but because of a significant IR contamination of some of the quartz samples, these were re-measured using a double SAR protocol in pulsed (POSL) mode. Although sample 111907 had a significant IR contamination the CW data is given for this sample, because no bounded dose estimates were derived in POSL mode. The column "In sat" gives the percentage of

multi-grain aliquots for which no bonded dose estimates could be derived because of saturation. The number of aliquots accepted into the respective dose distributions are given as “n”.

Core	Sample	Depth (cm)	Grain size (μm)	Hole Ø (μm)	# neg. D_e	N	In Sat (%)	OD (%)	n	Average age (ka)	CAM age (ka)	FMM_{prom} age (ka)	MAM age (ka)	IEU age (ka)	FMM_{min} age (ka)	Additional rejection criteria: D_0>x (Gy)	Average age (ka)	CAM age (ka)	n
SRH-5A	111919	235	180-250	300	0	4500	29%	43 ± 6	55	15.5 ± 1.2	14.3 ± 1.2	19 ± 2	8 ± 2	8.7 ± 0.8	8.3 ± 1.5	50	17 ± 2	16 ± 2	26
	**111921**	**355**	**180-250**	**300**	**0**	**4700**	**13%**	**55 ± 11**	**34**	**14 ± 2**	**12 ± 2**	**17 ± 2**	**5 ± 2**	**4.6 ± 0.7**	**5 ± 2**	**50**	**18 ± 2**	**16.6 ± 1.3**	**16**
	**111922**	**429**	**180-250**	**300**	**0**	**4800**	**20%**	**77 ± 13**	**37**	**18 ± 3**	**14 ± 2**	**25 ± 3**	**5 ± 2**	**3.5 ± 0.7**	**7.2 ± 1.4**	**50**	**16 ± 3**	**15 ± 3**	**13**
GS-7	111910	234	90-150	200	0	1200	12%	36 ± 5	76	6.7 ± 0.5	6.1 ± 0.4	6.1 ± 0.4	-	5.4 ± 0.4	6.1 ± 0.4	25	6.7 ± 0.5	6.1 ± 0.5	62
	111912	510	180-212	300	1	3500	4%	32 ± 6	73	5.1 ± 0.4	4.6 ± 0.3	4.6 ± 0.3	-	4.5 ± 0.3	4.6 ± 0.3	25	5.4 ± 0.4	5.3 ± 0.3	62
Gs-10	101901	431	150-180	200	1	2100	14%	57 ± 6	202	6.0 ± 0.4	4.9 ± 0.3	5.5 ± 0.5	0.3 ± 0.1	0.50 ± 0.09	1.9 ± 0.3	25	6.3 ± 0.5	5.3 ± 0.4	117
	**111902**	**780**	**180-250**	**300**	**0**	**1200**	**16%**	**61 ± 8**	**41**	**16 ± 2**	**13 ± 2**	**13.0 ± 1.0**	**1.7 ± 0.7**	**-**	**13.0 ± 1.0**	**50**	**19 ± 3**	**15 ± 2**	**25**
	**111903**	**1424**	**180-250**	**300**	**1**	**5900**	**8%**	**63 ± 11**	**69**	**22 ± 2**	**16 ± 2**	**10.1 ± 1.1**	**-**	**4.2 ± 0.7**	**10.1 ± 1.1**	**25**	**20 ± 3**	**15 ± 2**	**35**
	111904	1899	180-250	300	0	3800	25%	68 ± 10	39	38 ± 5	30 ± 4	50 ± 6	14 ± 3	14 ± 2	15 ± 3	100	63 ± 13	55 ± 15	6
	101902	2146	180-250	300	0	2300	21%	53 ± 8	42	48 ± 5	42 ± 4	43 ± 5	9 ± 4	11 ± 3	43 ± 5	100	49 ± 8	44 ± 11	6
	111905	2289	180-250	300	0	2400	36%	58 ± 14	18	46 ± 8	41 ± 7	55 ± 7	13 ± 5	15 ± 4	14 ± 5	100	57 ± 11	55 ± 9	5
	111906	2634	180-250	300	0	2400	40%	45 ± 6	59	32 ± 3	29 ± 2	29 ± 2	-	22 ± 2	29 ± 2	100	59 ± 12	54 ± 9	6
	111907	2904	180-250	300	0	3600	23%	82 ± 16	24	48 ± 8	35 ± 7	55 ± 6	8 ± 2	9 ± 3	9 ± 2	150	71 ± 12	65 ± 11	6
	111909	3689	180-250	300	3	7200	51%	72 ± 6	229	49 ± 4	44 ± 3	42 ± 7	-	13.2 ± 0.9	17 ± 3	200	152 ± 24	143 ± 21	9
GS-11	101903	50	180-212	300	2	3100	10%	72 ± 7	209	6.5 ± 0.5	4.9 ± 0.4	5.0 ± 0.5	0.2 ± 0.3	0.49 ± 0.10	1.9 ± 0.2	25	6.7 ± 0.6	5.0 ± 0.4	139
	101904	110	180-212	300	2	1800	22%	105 ± 19	61	12 ± 2	9.0 ± 1.4	8.5 ± 1.3	0.20 ± 0.05	0.23 ± 0.11	1.7 ± 0.2	50	19 ± 4	15 ± 3	29
	**091901**	**216**	**125-180**	**200**	**6**	**4800**	**19%**	**86 ± 11**	**115**	**10.2 ± 1.1**	**7.8 ± 0.8**	**17 ± 2**	**0.06 ± 0.07**	**0.09 ± 0.07**	**1.3 ± 0.3**	**75**	**17 ± 3**	**12 ± 2**	**23**
	091902	597	125-180	200	0	2400	47%	75 ± 12	34	18 ± 2	14 ± 2	21 ± 3	2.3 ± 1.2	2.2 ± 0.6	4.8 ± 1.4	75	19 ± 3	18 ± 3	4
	091906	697	180-250	300	0	1800	47%	70 ± 10	41	27 ± 3	22 ± 3	14 ± 7	4 ± 2	4.9 ± 1.0	14 ± 7	100	40 ± 5	36 ± 4	8
	101906	919	180-250	300	0	2400	26%	60 ± 9	37	26 ± 3	22 ± 3	13 ± 2	-	4.3 ± 1.4	13 ± 2	100	45 ± 4	47 ± 5	10
	091903	1272	180-250	300	0	3600	31%	52 ± 8	34	38 ± 5	33 ± 4	44 ± 6	-	13 ± 2	18 ± 6	100	52 ± 13	42 ± 8	8
	091907	1712	180-250	300	0	6900	46%	60 ± 8	63	44 ± 4	37 ± 4	53 ± 4	14 ± 2	10.7 ± 1.4	15 ± 2	125	54 ± 7	49 ± 7	14
	091904	2495	180-250	300	9	5700	19%	98 ± 12	99	31 ± 3	27 ± 3	56 ± 7	0.3 ± 0.2	0.4 ± 0.2	12 ± 2	125	60 ± 13	57 ± 13	8
	091905	3742	90-180	200	0	2400	63%	74 ± 10	41	98 ± 15	75 ± 10	122 ± 12	30 ± 6	25 ± 3	32 ± 5	200	173 ± 55	137 ± 33	7
GS-13	**111913**	**245**	**90-150**	**200**	**0**	**3300**	**47%**	**46 ± 9**	**24**	**18 ± 2**	**16 ± 2**	**12 ± 2**	**-**	**10.0 ± 1.2**	**12 ± 2**	**75**	**23 ± 5**	**19 ± 4**	**8**
	111914	507	90-125	200	0	7200	46%	75 ± 13	29	18 ± 3	14 ± 2	20 ± 3	1.8 ± 1.1	2.0 ± 0.6	5 ± 2	50	23 ± 5	17 ± 5	11
	111915	716	90-150	200	0	2300	48%	51 ± 7	54	26 ± 2	24 ± 2	32 ± 6	-	18.1 ± 1.5	15 ± 5	100	40 ± 6	38 ± 5	7
GS-14	**111916**	**210**	**180-212**	**300**	**0**	**1200**	**38%**	**64 ± 8**	**60**	**16 ± 2**	**12.2 ± 1.3**	**9.9 ± 1.1**	**-**	**5.7 ± 0.6**	**9.9 ± 1.1**	**50**	**22 ± 5**	**15 ± 3**	**19**
	**111917**	**412**	**180-212**	**300**	**0**	**2400**	**14%**	**58 ± 9**	**42**	**24 ± 3**	**20 ± 2**	**15 ± 2**	**-**	**-**	**15 ± 2**	**75**	**27 ± 4**	**23 ± 3**	**22**
	111918	585	180-212	300	1	2400	36%	59 ± 16	25	23 ± 3	19 ± 3	28 ± 3	-0.04 ± -0.36	-	9 ± 2	100	34 ± 8	28 ± 7	6

Supplementary Table 9. Summary of single grain data. The transition between fine medium sand and very fine grained facies lies between the two entries in bold in each core, except for core GS-13. The single grain measurements were made in single grain discs with hole diameters Ø of either 200 or 300 μm. "# neg. D_e" is the number of negative dose estimates in each dose distribution. "N" is the total number of grains measured. "In Sat" is the number of grains rejected solely because bonded dose estimates could not be derived. "OD" is the relative over-dispersion calculated solely based on counting statistics and curve fitting errors. "n" is the number of grains accepted into each dose distributions. "Average age" is the age calculated using the arithmetic mean of all accepted dose estimates. "CAM age" is the age calculated using all dose estimates and the Central Age Model (*52*). The CAM ages calculated for samples containing non-positive dose estimates is based on an assumption of normality. "FMM_{prom} age" is the age calculated based on the most prominent dose component identified by the Finite Mixture Model (FMM, (*72*)). All non-positive dose estimates were removed prior to application of the FMM. The FMM model was applied after an additional uncertainty of 30% was added in quadrature to individual dose estimates. "MAM age" is the age calculated based on the burial dose estimate identified by the Minimum Age Model (MAM, (*52*)). For the dose distributions containing dose estimated the MAM age has been derived by applying the original MAM scripts after doing a simple exponential transformation (*66*). The MAM model was applied after an additional uncertainty of 30% was added in quadrature to individual dose estimates. "MAM age (no neg)" are the MAM ages calculated after elimination of negative dose estimates. "IEU age" is the age calculated using the Internal/external consistency criterion (IEU, (*58*), (*78*)). "FMM_{min} age" is the age calculated based on the lowest dose component containing more than 10% of the grains identified by the FMM. All non-positive dose estimates were removed prior to application of the FMM. The FMM model was applied after an additional uncertainty of 30% was added in quadrature to individual dose estimates. "Average age" and "CAM age" using the two additional rejection criteria is based on the arithmetic mean and the CAM mean of the dose estimates left after rejecting grains with D_0 < 100 Gy (or 200 Gy for samples 111909 and 091905) and L_{ts}/T_{ts} ratios < 0.8, respectively.

Core	Sample	$n_{neg.}$	k	k_i	Age	se	p	se
SRH-5A	111919	0	2	1	8.3	1.5	0.29	0.12
				2	**19**	**2**	0.71	0.12
	111921	0	2	1	5	2	0.28	0.14
				2	**17**	**2**	0.72	0.14
	111922	0	2	1	7.2	1.4	0.46	0.12
				2	**25**	**3**	0.54	0.12
Gs-7	111910	0	1	1	**6.1**	**0.4**	1.00	0.00
	111912	1	1	1	**4.6**	**0.3**	1.00	0.00
Gs-10	101901	1	3	1	1.9	0.3	0.19	0.05
				2	**5.5**	**0.5**	0.65	0.07
				3	11.6	1.8	0.16	0.07
	111902	0	2	1	1.6	0.6	0.03	0.03
				2	**13.0**	**1.0**	0.97	0.03
	111903	1	2	1	10.1	1.1	0.58	0.09
				2	**33**	**4**	0.42	0.09
	111904	0	2	1	15	3	0.44	0.11
				2	**50**	**6**	0.56	0.11
	101902	0	3	1	9	4	0.06	0.05
				2	**43**	**5**	0.78	0.10
				3	105	25	0.16	0.10
	111905	0	2	1	14	5	0.24	0.12
				2	**55**	**7**	0.76	0.12
	111906	0	1	1	**29**	**2**	1.00	0.00
	111907	0	2	1	9	2	0.27	0.10
				2	**55**	**6**	0.73	0.10
	111909	3	3	1	17	3	0.27	0.09
				2	**42**	**7**	0.47	0.07
				3	104	13	0.26	0.07
Gs-11	101903	2	3	1	1.9	0.2	0.27	0.07
				2	**5.0**	**0.5**	0.52	0.06
				3	16.7	1.7	0.22	0.04
	101904	2	3	1	1.7	0.2	0.36	0.07
				2	**8.5**	**1.3**	0.42	0.09
				3	26	5	0.22	0.08
	091901	6	3	1	1.3	0.3	0.09	0.04
				2	5.3	0.9	0.35	0.09
				3	**16.9**	**1.7**	0.56	0.09
	091902	0	2	1	4.8	1.4	0.25	0.11
				2	**21**	**3**	0.75	0.11

Core	Sample	$n_{neg.}$	k	k_i	Age	se	p	se
Gs-11	091906	0	2	1	14	7	0.51	0.39
				2	**34**	**11**	0.49	0.39
	101906	0	2	1	13	2	0.52	0.13
				2	**38**	**6**	0.48	0.13
	091903	0	2	1	18	6	0.30	0.20
				2	**44**	**6**	0.70	0.20
	091907	0	2	1	15	2	0.29	0.07
				2	**53**	**4**	0.71	0.07
	091904	9	2	1	12	2	0.49	0.10
				2	**56**	**7**	0.51	0.10
	091905	0	2	1	32	5	0.37	0.09
				2	**122**	**12**	0.63	0.09
Gs-13	111913	0	2	1	**12**	**2**	0.68	0.19
				2	27	7	0.32	0.19
	111914	0	2	1	5	2	0.29	0.12
				2	**20**	**3**	0.71	0.12
	111915	0	2	1	15	5	0.38	0.32
				2	**32**	**6**	0.62	0.32
Gs-14	111916	0	2	1	**9.9**	**1.1**	0.78	0.10
				2	29	7	0.22	0.10
	111917	0	2	1	**15**	**2**	0.65	0.15
				2	38	7	0.35	0.15
	111918	1	2	1	9	2	0.29	0.12
				2	**28**	**3**	0.71	0.12

Supplementary Table 10.

FMM results for all natural single-grain dose distributions using an additional uncertainty of 30%. "$n_{neg.}$" is the number of non-positive dose estimates removed prior to running the FMM. "k" is the number of components. The "Age" of each component is given in units of ka at 1 standard error and "p" is the proportion of grains attributed to each dose component. The ages highlighted in boldface are the most prominent ages with the exception of 111903, 091906 and 101906 (marked in red), where we have chosen the larger dose component.

Supplementary Table 11

Core	Sample	$n_{neg.}$	llik	BIC	Add (%)	k	FMM_{prom}					$FMM_{prom,2nd}$				
							k_i	Age (ka)	se	p	se	k_i	Age (ka)	se	p	se
SRH-5A	111919	0	-46	105	0	2	2	20.1	1.1	0.67	0.08	1	8.2	0.5	0.33	0.08
			-42	96	10	2	2	20.3	1.3	0.66	0.08	1	8.3	0.7	0.34	0.08
			**-41**	**94**	**20**	**2**	**2**	19.6	1.5	0.68	0.09	1	8.2	1.0	0.32	0.09
			-42	97	30	2	2	18.6	1.8	0.71	0.12	1	8.3	1.5	0.29	0.12
			-45	93	40	1	1	46.5	3.3	1.00						
	111921	0	-39	90	10	2	2	17.6	1.2	0.62	0.10	1	6.6	1.0	0.38	0.10
			-38	86	20	2	2	17.5	1.7	0.66	0.12	1	6.0	1.4	0.34	0.12
			**-37**	**85**	**30**	**2**	**2**	**16.6**	**2.0**	**0.72**	**0.14**	1	5.3	1.7	0.28	0.14
			-40	84	40	1	1	12.4	1.5	1.00						
	111922	0	-54	118	10	2	2	24.8	1.9	0.52	0.10	1	8.6	1.0	0.48	0.10
			-51	112	20	2	2	25.5	2.5	0.52	0.10	1	7.8	1.1	0.48	0.10
			-50	110	30	2	2	25.1	3.3	0.54	0.12	1	7.2	1.4	0.46	0.12
			**-49**	**110**	**40**	**2**	**2**	**23.3**	**4.2**	**0.60**	**0.16**	1	6.4	2.1	0.40	0.16
			-53	109	50	1	1	14.3	2.2	1.00						
Gs-7	111910	0	-62	137	5	2	2	7.1	0.3	0.78	0.07	1	3.3	0.1	0.22	0.07
			-59	131	10	2	2	7.2	0.5	0.78	0.07	1	3.3	0.3	0.22	0.07
			-56	125	20	2	2	6.9	0.6	0.83	0.12	1	3.3	0.6	0.17	0.12
			-55	122	30	1	1	6.1	0.4	1.00						
Gs-10	111902	0	-31	81	5	3	2	11.6	0.7	0.82	0.07	3	29.4	5.2	0.15	0.07
			-30	78	10	3	2	11.5	0.7	0.82	0.07	3	28.9	5.5	0.15	0.07
			-30	78	15	3	2	11.5	0.8	0.83	0.08	3	28.4	5.8	0.14	0.07
			-30	79	20	3	2	11.5	0.9	0.83	0.08	3	28.0	6.4	0.14	0.07
			-31	80	25	3	2	11.6	1.0	0.84	0.08	3	27.6	7.3	0.13	0.08
			-34	78	30	2	2	13.0	1.0	0.97	0.03	1	1.6	0.7	0.03	0.03
			-33	78	35	2	2	13.0	1.1	0.97	0.03	1	1.6	0.7	0.03	0.03
			-34	78	40	2	2	12.9	1.2	0.97	0.03	1	1.6	0.8	0.03	0.03
			-38	80	45	1	1	12.4	1.3	1.00						
			-38	79	50	1	1	12.4	1.3	1.00						
	111903	1	-81	191	5	4	2	10.4	0.8	0.45	0.08	3	27.3	2.2	0.29	0.08
			-80	190	10	4	2	10.8	4.9	0.45	0.09	3	26.4	7.8	0.29	0.09
			-80	191	15	4	2	11.1	1.1	0.45	0.10	3	26.1	3.4	0.29	0.09
			-86	185	20	2	1	9.8	0.9	0.57	0.08	2	33.8	3.1	0.43	0.08
			-84	181	25	2	1	9.9	1.0	0.58	0.08	2	33.6	3.5	0.42	0.08
			-83	179	30	2	1	10.1	1.1	0.58	0.09	2	33.4	4.0	0.42	0.09
			-83	178	35	2	1	10.3	1.3	0.58	0.10	2	33.0	4.7	0.42	0.10
			-82	177	40	2	1	10.5	1.5	0.58	0.12	2	32.5	5.5	0.42	0.12
			-82	178	45	2	1	10.8	1.8	0.59	0.15	2	31.7	6.4	0.41	0.15
			-83	178	50	2	1	11.1	2.2	0.59	0.19	2	30.5	7.6	0.41	0.19
	111904	0	-50	111	10	4	2	51.1	3.7	0.55	0.10	1	15.0	1.9	0.45	0.10
			-46	104	20	3	2	51.0	4.7	0.56	0.10	1	14.9	2.1	0.44	0.10
			-45	101	30	2	2	50.1	6.0	0.56	0.11	1	15.0	2.6	0.44	0.11
			-45	101	40	2	2	48.3	7.6	0.58	0.14	1	15.3	3.4	0.42	0.14

Supplementary Table 11. Detailed FMM results for 16 of the 30 natural single-grain dose distributions (see full caption below)

Supplementary Table 11 continued

Core	Sample	$n_{neg.}$	llik	BIC	Add (%)	k	FMM_{prom}					$FMM_{prom,2nd}$				
							k_i	Age (ka)	se	p	se	k_i	Age (ka)	se	p	se
Gs-10	111906	0	-50	128	5	4	2	23.1	3.3	0.40	0.22	4	45.9	3.8	0.34	0.12
			-54	120	10	2	1	20.9	2.3	0.52	0.12	2	42.6	3.6	0.48	0.12
			-53	118	15	2	2	41.2	4.0	0.53	0.15	1	19.8	2.9	0.47	0.15
			-52	116	20	2	2	39.3	4.3	0.59	0.17	1	18.6	3.5	0.41	0.17
			-51	115	25	2	2	36.5	5.1	0.71	0.24					
			-55	113	30	1	1	28.9	2.5	1.00						
			-53	109	35	1	1	28.9	2.5	1.00						
			-52	107	40	1	1	28.9	2.5	1.00						
	111907	0	-26	81	0	5	4	47.1	4.0	0.44	0.13	5	97.9	12.5	0.22	0.10
			-29	74	10	3	2	42.0	3.7	0.49	0.12	1	9.2	1.9	0.27	0.10
			-29	73	20	3	2	43.5	5.2	0.50	0.14	1	8.9	2.0	0.27	0.10
			-30	69	30	2	2	54.6	6.0	0.73	0.10	1	8.8	2.2	0.27	0.10
			-29	68	40	2	2	54.4	7.3	0.74	0.10	1	8.4	2.5	0.26	0.10
			-30	69	50	2	2	53.1	8.5	0.76	0.10	1	8.1	3.0	0.24	0.10
	111909	3	-310	669	5	5	3	32.7	2.2	0.29	0.05	4	62.7	3.4	0.27	0.05
			-287	622	10	5	3	33.9	2.7	0.31	0.06	4	66.9	4.5	0.28	0.05
			-285	609	15	4	2	25.2	1.7	0.44	0.05	3	59.4	4.7	0.34	0.05
			-280	599	20	4	2	24.8	1.9	0.43	0.06	3	58.3	5.7	0.35	0.05
			-279	595	25	4	2	24.6	2.3	0.43	0.07	3	57.6	7.6	0.35	0.06
			-284	594	27.5	3	2	43.8	5.7	0.47	0.07	3	108.4	11.7	0.24	0.06
			-282	592	30	3	2	42.1	6.8	0.47	0.07	3	104.4	12.8	0.26	0.07
			-281	590	32.5	3	2	28.9	2.9	0.55	0.06	3	87.9	7.5	0.39	0.06
			-280	587	35	3	2	28.8	3.1	0.55	0.06	3	86.8	7.8	0.39	0.06
			-282	581	40	2	1	24.3	2.3	0.54	0.06	2	75.6	7.9	0.48	0.08
			-280	577	45	2	1	24.4	2.6	0.52	0.08	2	75.6	7.9	0.48	0.08
			-280	576	50	2	1	24.7	3.1	0.51	0.10	2	72.1	8.8	0.49	0.10
			-280	576	55	2	2	67.8	9.9	0.51	0.13	1	25.8	5.4	0.46	0.19
			-280	577	60	2	2	62.3	11.1	0.54	0.19	1	25.8	5.4	0.46	0.19
Gs-11	091906	0	-41	100	10	3	2	22.0	2.1	0.38	0.13	1	11.0	1.7	0.33	0.12
			-44	98	20	2	1	16.7	1.8	0.67	0.11	2	39.9	4.9	0.33	0.11
			-42	96	30	2	1	13.9	6.6	0.51	0.39	2	33.5	10.9	0.49	0.39
			-42	95	40	1	1	21.5	2.8	1.00	0.00					
	101906	0	-43	105	5	3	2	17.2	2.1	0.47	0.12	3	50.0	3.2	0.36	0.10
			-41	101	10	3	2	16.8	2.2	0.45	0.12	3	46.0	3.7	0.39	0.10
			-44	99	15	2	1	13.1	1.3	0.57	0.10	2	43.1	4.0	0.43	0.10
			-43	97	20	2	1	12.9	1.5	0.56	0.10	2	41.4	4.5	0.44	0.10
			-43	96	25	2	1	12.7	1.6	0.54	0.11	2	39.7	5.1	0.46	0.11
			-42	96	30	2	2	12.6	1.9	0.52	0.13	2	37.8	5.7	0.48	0.13
			-42	96	35	2	2	35.4	6.5	0.52	0.16	1	12.3	2.4	0.48	0.16
			-42	96	40	2	2	32.5	7.3	0.58	0.23	1	11.8	3.3	0.42	0.23
			-46	95	45	1	1	21.9	2.7	1.00						
	091903	0	-39	95	0	3	2	33.4	2.3	0.49	0.11	3	52.8	2.9	0.33	0.09
			-34	86	10	3	2	32.5	2.6	0.51	0.11	3	58.1	4.7	0.31	0.09
			-33	83	15	3	2	32.7	3.7	0.53	0.12	3	60.0	7.0	0.29	0.11
			-35	81	20	2	2	46.6	4.9	0.62	0.16	1	19.3	3.9	0.38	0.16
			-33	76	30	2	2	43.6	6.0	0.70	0.20	1	17.6	6.0	0.30	0.20
			-34	72	40	1	1	33.3	3.7	1.00						

Supplementary Table 11 (continued).

Detailed FMM results for 16 of the 30 natural single-grain dose distributions (see full caption below)

Supplementary Table 11 continued

Core	Sample	$n_{neg.}$	llik	BIC	Add (%)	k	FMM_{prom}					$FMM_{prom,2nd}$				
							k_i	Age (ka)	se	p	se	k_i	Age (ka)	se	p	se
Gs-11	091907	0	-52	124	5	3	3	54.8	2.9	0.68	0.07	1	11.6	1.4	0.19	0.06
			-52	124	10	3	3	55.2	3.1	0.68	0.07	1	11.6	1.5	0.19	0.07
			-55	123	15	2	2	55.2	3.4	0.69	0.07	1	16.4	1.6	0.31	0.07
			-55	123	20	2	2	55.0	3.7	0.69	0.07	1	15.9	1.7	0.31	0.07
			-56	125	25	2	2	54.4	4.1	0.70	0.07	1	15.4	2.0	0.30	0.07
			-58	128	30	2	2	53.5	4.5	0.71	0.07	1	14.9	2.3	0.29	0.07
			-59	131	35	2	2	52.2	4.8	0.73	0.08	1	14.3	2.6	0.27	0.08
			-61	133	40	2	2	50.7	5.1	0.75	0.08	1	13.8	3.0	0.25	0.08
			-62	136	45	2	2	49.2	5.4	0.77	0.08	1	13.5	3.4	0.23	0.08
			-67	138	50	1	1	37.6	3.8	1.00	0.00					
	091904	9	-183	380	10	2	1	16.2	0.6	0.61	0.06	2	70.4	2.8	0.39	0.06
			-151	316	20	2	1	15.0	0.6	0.58	0.06	2	65.7	2.6	0.42	0.06
			-138	290	30	2	2	56.5	6.7	0.51	0.10	1	12.1	2.4	0.49	0.10
			-133	280	40	2	2	51.4	5.6	0.56	0.09	1	10.6	2.1	0.44	0.09
	091905	0	-72	156	5	2	2	115.1	7.2	0.63	0.08	1	32.8	3.0	0.37	0.08
			-67	145	10	2	2	117.6	7.9	0.63	0.08	1	32.5	3.2	0.37	0.08
			-62	134	15	2	2	119.7	8.8	0.63	0.08	1	32.2	3.4	0.37	0.08
			-57	126	20	2	2	121.0	9.8	0.63	0.08	1	32.0	3.7	0.37	0.08
			-55	121	25	2	2	121.6	10.9	0.63	0.08	1	31.8	4.1	0.37	0.08
			-53	118	30	2	2	121.6	12.1	0.63	0.09	1	31.8	4.7	0.37	0.09
			-52	116	35	2	2	121.1	13.5	0.63	0.09	1	32.1	5.4	0.37	0.09
			-52	115	40	2	2	119.9	15.3	0.63	0.10	1	32.7	6.5	0.37	0.10
			-52	115	45	2	2	118.2	17.5	0.63	0.12	1	33.8	8.0	0.38	0.12
			-52	115	50	2	2	115.9	20.4	0.62	0.15	1	35.5	10.3	0.38	0.15
			-52	116	55	1	1	74.6	10.3	1.00						

Supplementary Table 11 (continued).

Detailed FMM results for 16 of the 30 natural single-grain dose distributions for the most prominent (FMM_{prom}) and second most prominent ($FMM_{prom,2nd}$) grain populations." n_{neg}." is the number of non-positive dose estimates removed prior to running the FMM. "llik" and "BIC" are the log likelihood and the Bayesian Information Criterion, respectively. "Add (%)" is the additional uncertainty added on to the uncertainties assigned to individual dose estimates based on Poisson statistics and curve fitting uncertainties. "k" is the total number of components. The "Age" of each component is given in units of ka at 1 standard error and "p" is the proportion of grains attributed to each dose component. "FMM_{prom}" is the most prominent component (i.e. the component with the highest value of p), and "$FMM_{prom,2nd}$" is the second largest component. The ages highlighted in boldface are the ages obtained by optimising llik and BIC.

Supplementary References

1 Pal, Y., Sahai, B., Sood, R. K. & Agrawal, D. P. Remote sensing of the lost Sarasvati river. *Proc. Ind. Acad. Sci.* **89**, 317-331 (1980).

2 Liu, J. G. Balance contrast enhancement technique and its application in image colour composition. *Int. J. Rem. Sen.* **12**, 2133-2151 (1990).

3 Liu, J. G. Direct decorrelation stretch technique for RGB colour composition. *Int. J. Rem. Sen.* **17**, 1005-1018 (1996).

4 Farr, T. G. *et al.* The shuttle radar topography mission. *Reviews of Geophysics* **45**, doi:10.1029/2005rg000183 (2007).

5 Wentworth, C. K. A Scale of Grade and Class Terms for Clastic Sediments. *The Journal of Geology* **30**, 377-392 (1922).

6 Blatt, H. *Sedimentary Petrology*. 514 (Freeman, 1992).

7 Bøtter-Jensen, L., Thomsen, K. J. & Jain, M. Review of optically stimulated luminescence (OSL) instrumental developments for retrospective dosimetry. *Radiation Measurements* **45**, 253-257, doi:10.1016/j.radmeas.2009.11.030 (2010).

8 Lapp, T., Jain, M., Ankjaergaard, C. & Pirtzel, L. Development of pulsed stimulation and Photon Timer attachments to the Riso TL/OSL reader. *Radiation Measurements* **44**, 571-575, doi:10.1016/j.radmeas.2009.01.012 (2009).

9 Ankjaergaard, C., Jain, M., Thomsen, K. J. & Murray, A. S. Optimising the separation of quartz and feldspar optically stimulated luminescence using pulsed excitation. *Radiation Measurements* **45**, 778-785, doi:10.1016/j.radmeas.2010.03.004 (2010).

10 Bøtter-Jensen, L., Andersen, C. E., Duller, G. A. T. & Murray, A. S. Developments in radiation, stimulation and observation facilities in luminescence measurements. *Radiation Measurements* **37**, 535-541, doi:10.1016/s1350-4487(03)00020-9 (2003).

11 Lapp, T., Jain, M., Thomsen, K. J., Murray, A. S. & Buylaert, J. P. New luminescence measurement facilities in retrospective dosimetry. *Radiation Measurements* **47**, 803-808, doi:10.1016/j.radmeas.2012.02.006 (2012).

12 Murray, A. S. & Wintle, A. G. Luminescence dating of quartz using an improved single-aliquot regenerative-dose protocol. *Radiation Measurements* **32**, 57-73, doi:10.1016/s1350-4487(99)00253-x (2000).

13 Auclair, M., Lamothe, M. & Huot, S. Measurement of anomalous fading for feldspar IRSL using SAR. *Radiation Measurements* **37**, 487-492 (2003).

14 Thiel, C. *et al.* Luminescence dating of the Stratzing loess profile (Austria) - Testing the potential of an elevated temperature post-IR IRSL protocol. *Quaternary International* **234**, 23-31, doi:10.1016/j.quaint.2010.05.018 (2011).

15 Murray, A. S. & Wintle, A. G. The single aliquot regenerative dose protocol: potential for improvements in reliability. *Radiation Measurements* **37**, 377-381, doi:10.1016/s1350-4487(03)00053-2 (2003).

16 Cunningham, A. C. & Wallinga, J. Selection of integration time intervals for quartz OSL decay curves. *Quaternary Geochronology* **5**, 657-666, doi:10.1016/j.quageo.2010.08.004 (2010).

17 Duller, G. A. T. Assessing the error on equivalent dose estimates derived from single aliquot regenerative dose measurements. *Ancient TL* **25**, 15-24 (2007).

18 Duller, G. A. T. Distinguishing quartz and feldspar in single grain luminescence measurements. *Radiation Measurements* **37**, 161-165, doi:10.1016/s1350-4487(02)00170-1 (2003).

19 Murray, A. S., Marten, R., Johnson, A. & Martin, P. Analysis for naturally occurring radionuclides at environmental concentrations by gamma spectrometry *Journal of Radioanalytical and Nuclear Chemistry Articles* **115**, 263-288 (1987).

20 Guérin, G., Mercier, N. & Adamiec, G. Dose-rate conversion factors: update. *Ancient TL* **29**, 5-8 (2011).
21 Huntley, D. J. & Baril, M. R. The K content of the K-feldspars being measured in optical dating or in thermoluminescence dating *Ancient TL* **15**, 11-13 (1997).
22 Aitken, M. J. *Thermoluminescence Dating*. 359 p. (Academic Press, 1985).
23 Prescott, J. R. & Hutton, J. T. Cosmic-ray contributions to dose-rates for luminescence and esr dating - large depths and long-term time variations. *Radiation Measurements* **23**, 497-500, doi:10.1016/1350-4487(94)90086-8 (1994).
24 Jain, M., Guralnik, B. & Andersen, M. T. Stimulated luminescence emission from localized recombination in randomly distributed defects. *Journal of Physics-Condensed Matter* **24**, doi:10.1088/0953-8984/24/38/385402 (2012).
25 Buylaert, J. P. *et al.* A robust feldspar luminescence dating method for Middle and Late Pleistocene sediments. *Boreas* **41**, 435-451, doi:10.1111/j.1502-3885.2012.00248.x (2012).
26 Sohbati, R. *et al.* Na-rich feldspar as a luminescence dosimeter in infrared stimulated luminescence (IRSL) dating. *Radiation Measurements* **51-52**, 67-82, doi:10.1016/j.radmeas.2012.12.011 (2013).
27 Buylaert, J. P., Huot, S., Murray, A. S. & Van den Haute, P. Infrared stimulated luminescence dating of an Eemian (MIS 5e) site in Denmark using K-feldspar. *Boreas* **40**, 46-56, doi:10.1111/j.1502-3885.2010.00156.x (2011).
28 Godfreysmith, D. I., Huntley, D. J. & Chen, W. H. Optical dating studies of quartz and feldspar sediment extracts. *Quaternary Science Reviews* **7**, 373-380, doi:10.1016/0277-3791(88)90032-7 (1988).
29 Thomsen, K. J., Murray, A. S., Jain, M. & Botter-Jensen, L. Laboratory fading rates of various luminescence signals from feldspar-rich sediment extracts. *Radiation Measurements* **43**, 1474-1486, doi:10.1016/j.radmeas.2008.06.002 (2008).
30 Thomsen, K. J. *et al.* Minimizing feldspar OSL contamination in quartz UV-OSL using pulsed blue stimulation. *Radiation Measurements* **43**, 752-757, doi:10.1016/j.radmeas.2008.01.020 (2008).
31 Buylaert, J. P., Murray, A. S. & Huot, S. Optical dating of an Eemian site in Northern Russia using K-feldspar. *Radiation Measurements* **43**, 715-720, doi:10.1016/j.radmeas.2008.01.027 (2008).
32 Murray, A. S., Thomsen, K. J., Masuda, N., Buylaert, J. P. & Jain, M. Identifying well-bleached quartz using the different bleaching rates of quartz and feldspar luminescence signals. *Radiation Measurements* **47**, 688-695, doi:10.1016/j.radmeas.2012.05.006 (2012).
33 Alexanderson, H. & Murray, A. S. Problems and potential of OSL dating Weichselian and Holocene sediments in Sweden. *Quaternary Science Reviews* **44**, 37-50, doi:10.1016/j.quascirev.2009.09.020 (2012).
34 Blomdin, R. *et al.* Timing of the deglaciation in southern Patagonia: Testing the applicability of K-Feldspar IRSL. *Quaternary Geochronology* **10**, 264-272, doi:10.1016/j.quageo.2012.02.019 (2012).
35 Ollerhead, J. & Huntley, D. J. Optical dating of young feldspars: the zeroing question. *Ancient TL* **29** (2011).
36 Jain, M., Murray, A. S. & Bøtter-Jensen, L. Optically stimulated luminescence dating: How significant is incomplete light exposure in fluvial environments? *Quaternaire* **15**, 143-157 (2004).
37 Murray, A. S. & Funder, S. Optically stimulated luminescence dating of a Danish Eemian coastal marine deposit: a test of accuracy. *Quaternary Science Reviews* **22**, 1177-1183, doi:10.1016/s0277-3791(03)00048-9 (2003).

38 Murray, A. S., Wintle, A. G. & Wallinga, J. Dose estimation using quartz OSL in the non-linear region of the growth curve. *Radiation Protection Dosimetry* **101**, 371-374 (2002).
39 Wintle, A. G. & Murray, A. S. A review of quartz optically stimulated luminescence characteristics and their relevance in single-aliquot regeneration dating protocols. *Radiation Measurements* **41**, 369-391, doi:10.1016/j.radmeas.2005.11.001 (2006).
40 Thomsen, K. J. *et al.* Testing single-grain quartz OSL methods using known age samples from the Bordes-Fitte rockshelter (Roches d'Abilly site, Central France) *Quaternary Geochronology.* **31**, 77-96, doi:10.1016/j.quageo.2015.11.002 (2016).
41 Galbraith, R. F. & Roberts, R. G. Statistical aspects of equivalent dose and error calculation and display in OSL dating: an overview and some recommendations. *Quaternary Geochronology* **11**, 1-27 (2012).
42 Banerjee, D., Murray, A. S., Botter-Jensen, L. & Lang, A. Equivalent dose estimation using a single aliquot of polymineral fine grains. *Radiation Measurements* **33**, 73-94, doi:10.1016/s1350-4487(00)00101-3 (2001).
43 Wallinga, J., Murray, A. S. & Bøtter-Jensen, L. *Measurement of the dose in quartz in the presence of feldspar contamination*. Proceeding Solid State Dosimetry, A367-A370 (2002).
44 Sanderson, D. C. W. & Clark, R. J. Pulsed photostimulated luminescence of alkali feldspars. *Radiation Measurements* **23**, 633-639, doi:10.1016/1350-4487(94)90112-0 (1994).
45 Clark, R. J., Bailiff, I. K. & Tooley, M. J. A preliminary study of time-resolved luminescence in some feldspars. *Radiation Measurements* **27**, 211-220, doi:10.1016/s1350-4487(96)00123-0 (1997).
46 Denby, P. M., Botter-Jensen, L., Murray, A. S., Thomsen, K. J. & Moska, P. Application of pulsed OSL to the separation of the luminescence components from a mixed quartz/feldspar sample. *Radiation Measurements* **41**, 774-779, doi:10.1016/j.radmeas.2006.05.017 (2006).
47 Murray, A. S. & Roberts, R. G. Determining the burial time of single grains of quartz using optically stimulated luminescence. *Earth and Planetary Science Letters* **152**, 163-180, doi:10.1016/s0012-821x(97)00150-7 (1997).
48 Olley, J. M., Caitcheon, G. G. & Roberts, R. G. The origin of dose distributions in fluvial sediments, and the prospect of dating single grains from fluvial deposits using optically stimulated luminescence. *Radiation Measurements* **30**, 207-217, doi:10.1016/s1350-4487(99)00040-2 (1999).
49 Roberts, R. *et al.* Single-aliquot and single-grain optical dating confirm thermoluminescence age estimates at Malakunanja II rock shelter in northern Australia. *Ancient TL* **16**, 19-24 (1998).
50 Jacobs, Z., Duller, G. A. T. & Wintle, A. G. Optical dating of dune sand from Blombos Cave, South Africa: II - single grain data. *Journal of Human Evolution* **44**, 613-625, doi:10.1016/s0047-2484(03)00049-6 (2003).
51 Jain, M., Murray, A. S. & Botter-Jensen, L. Characterisation of blue-light stimulated luminescence components in different quartz samples: implications for dose measurement. *Radiation Measurements* **37**, 441-449, doi:10.1016/s1350-4487(03)00052-0 (2003).
52 Roberts, R. G., Galbraith, R. F., Olley, J. M., Yoshida, H. & Laslett, G. M. Optical dating of single and multiple grains of quartz from jinmium rock shelter, northern Australia, part 2, Results and implications. *Archaeometry* **41**, 365-395, doi:10.1111/j.1475-4754.1999.tb00988.x (1999).

53 Yoshida, H., Roberts, R. G., Olley, J. M., Laslett, G. M. & Galbraith, R. F. Extending the age range of optical dating using single 'supergrains' of quartz. *Radiation Measurements* **32**, 439-446, doi:10.1016/s1350-4487(99)00287-5 (2000).
54 Galbraith, R. F., Roberts, R. G., Laslett, G. M., Yoshida, H. & Olley, J. M. Optical dating of single and multiple grains of quartz from jinmium rock shelter, northern Australia, part 1, Experimental design and statistical models. *Archaeometry* **41**, 339-364, doi:10.1111/j.1475-4754.1999.tb00987.x (1999).
55 Thomsen, K. J., Murray, A. & Jain, M. The dose dependency of the over-dispersion of quartz OSL single grain dose distributions. *Radiation Measurements* **47**, 732-739, doi:10.1016/j.radmeas.2012.02.015 (2012).
56 Guérin, G. *et al.* A Multi-method Luminescence Dating of the Palaeolithic Sequence of La Ferrassie Based on New Excavations Adjacent to the La Ferrassie 1 and 2 Skeletons. *Journal of Human Evolution.* **58**, 147-166, doi:10.1016/j.jas.2015.01.019 (2015).
57 Duller, G. A. T. & Murray, A. S. Luminescence dating of sediments using individual mineral grains *Geologos* **5**, 87-106 (2000).
58 Duller, G. A. T. Single-grain optical dating of Quaternary sediments: why aliquot size matters in luminescence dating. *Boreas* **37**, 589-612, doi:10.1111/j.1502-3885.2008.00051.x (2008).
59 Thomsen, K. J., Murray, A. S., Bøtter-Jensen, L. & Kinahan, J. Determination of burial dose in incompletely bleached fluvial samples using single grains of quartz. *Radiation Measurements* **42**, 370-379, doi:10.1016/j.radmeas.2007.01.041 (2007).
60 Singarayer, J. S. & Bailey, R. M. Further investigations of the quartz optically stimulated luminescence components using linear modulation. *Radiation Measurements* **37**, 451-458, doi:10.1016/s1350-4487(03)00062-3 (2003).
61 Choi, J. H., Murray, A. S., Jain, M., Cheong, C. S. & Chang, H. W. Luminescence dating of well-sorted marine terrace sediments on the southeastern coast of Korea. *Quaternary Science Reviews* **22**, 407-421, doi:10.1016/s0277-3791(02)00136-1 (2003).
62 Jain, M., Murray, A. S., Bøtter-Jensen, L. & Wintle, A. G. A single-aliquot regenerative-dose method based on IR (1.49 eV) bleaching of the fast OSL component in quartz. *Radiation Measurements* **39**, 309-318, doi:10.1016/j.radmeas.2004.05.004 (2005).
63 Ballarini, M., Wallinga, J., Wintle, A. G. & Bos, A. J. J. A modified SAR protocol for optical dating of individual grains from young quartz samples. *Radiation Measurements* **42**, 360-369, doi:10.1016/j.radmeas.2006.12.016 (2007).
64 Reimann, T., Lindhorst, S., Thomsen, K. J., Murray, A. S. & Frechen, M. OSL dating of mixed coastal sediment (Sylt, German Bight, North Sea). *Quaternary Geochronology* **11**, 52-67, doi:10.1016/j.quageo.2012.04.006 (2012).
65 Jacobs, Z., Hayes, E. H., Roberts, R. G., Galbraith, R. F. & Henshilwood, C. S. An improved OSL chronology for the Still Bay layers at Blombos Cave, South Africa: further tests of single-grain dating procedures and a re-evaluation of the timing of the Still Bay industry across southern Africa. *Journal of Archaeological Science* **40**, 579-594, doi:10.1016/j.jas.2012.06.037 (2013).
66 Sim, A. K. *et al.* Dating recent floodplain sediments in the Hawkesbury-Nepean River system, eastern Australia using single-grain quartz OSL. *Boreas* **43**, 1-21, doi:10.1111/bor.12018 (2014).
67 Medialdea, A., Thomsen, K. J., Murray, A. S. & Benito, G. Reliability of equivalent-dose determination and age-models in the OSL dating of historical and modern

palaeoflood sediments. *Quaternary Geochronology* **22**, 11-24, doi:10.1016/j.quageo.2014.01.004 (2014).
68 Madsen, A. T. & Murray, A. S. Optically stimulated luminescence dating of young sediments: A review. *Geomorphology* **109**, 3-16, doi:10.1016/j.geomorph.2008.08.020 (2009).
69 Durcan, J. A. & Duller, G. A. T. The fast ratio: A rapid measure for testing the dominance of the fast component in the initial OSL signal from quartz. *Radiation Measurements* **46**, 1065-1072, doi:10.1016/j.radmeas.2011.07.016 (2011).
70 Duller, G. A. T. Improving the accuracy and precision of equivalent doses determined using the optically stimulated luminescence signal from single grains of quartz. *Radiation Measurements* **47**, 770-777, doi:10.1016/j.radmeas.2012.01.006 (2012).
71 Feathers, J. Luminescence dating at Diepkloof Rock Shelter - new dates from single-grain quartz. *Journal of Archaeological Science* **63**, 164-174, doi:10.1016/j.jas.2015.02.012 (2015).
72 Gliganic, L. A., Jacobs, Z. & Roberts, R. G. Luminescence characteristics and dose distributions for quartz and feldspar grains from Mumba rockshelter, Tanzania. *Archaeological and Anthropological Sciences* **4**, 115-135, doi:10.1007/s12520-011-0085-9 (2012).
73 Arnold, L. J. & Roberts, R. G. Paper I - Optically stimulated luminescence (OSL) dating of perennially frozen deposits in north-central Siberia: OSL characteristics of quartz grains and methodological considerations regarding their suitability for dating. *Boreas* **40**, 389-416 (2011).
74 Slama, J. *et al.* Plesovice zircon - A new natural reference material for U-Pb and Hf isotopic microanalysis. *Chemical Geology* **249**, 1-35, doi:10.1016/j.chemgeo.2007.11.005 (2008).
75 Jochum, K. P. *et al.* Determination of Reference Values for NIST SRM 610–617 Glasses Following ISO Guidelines. *Geostandards and Geoanalytical Research* **35**, 397-429, doi:10.1111/j.1751-908X.2011.00120.x (2011).
76 Kuiper, K. F. *et al.* Synchronizing rock clocks of Earth history. *Science* **320**, 500-504, doi:10.1126/science.1154339 (2008).
77 Steiger, R. H. & Jager, E. Subcommission on geochronology - convention on use of decay constants in geochronology and cosmochronology. *Earth and Planetary Science Letters* **36**, 359-362, doi:10.1016/0012-821x(77)90060-7 (1977).
78 Nier, A. O. A redetermination of the relative abundances of the isotopes of carbon, nitrogen, oxygen, argon and potassium. *Physical Reviews* **77**, 780-793 (1950).
79 Galbraith, R. F. & Green, P. F. Estimating the component ages in a finite mixture. *Nuclear Tracks and Radiation Measurements* **17**, 197-206 (1990).
80 Guérin, G., Murray, A. S., Jain, M., Thomsen, K. J. & Mercier, N. How confident are we in the chronology of the transition between Howieson's Poort and Still Bay? *Journal of Archaeological Science* **64**, 314-317, doi:10.1016/j.jhevol.2013.01.006 (2013).
81 Arnold, L. J., Roberts, R. G., Galbraith, R. F. & DeLong, S. B. A revised burial dose estimation procedure for optical dating of young and modern-age sediments. *Quaternary Geochronology* **4**, 306-325, doi:10.1016/j.quageo.2009.02.017 (2009).
82 Demuro, M., Froese, D. G., Arnold, L. J. & Roberts, R. G. Single-grain OSL dating of glaciofluvial quartz constrains Reid glaciation in NW Canada to MIS 6. *Quaternary Research* **77**, 305-316, doi:10.1016/j.yqres.2011.11.009 (2012).
83 Olley, J. M., Pietsch, T. & Roberts, R. G. Optical dating of Holocene sediments from a variety of geomorphic settings using single grains of quartz. *Geomorphology* **60**, 337-358, doi:10.1016/j.geomorph.2003.09.020 (2004).

84 Bailey, R. M. & Arnold, L. J. Statistical modelling of single grain quartz D-e distributions and an assessment of procedures for estimating burial dose. *Quaternary Science Reviews* **25**, 2475-2502, doi:10.1016/j.quascirev.2005.09.012 (2006).
85 Fuchs, M. & Owen, L. A. Luminescence dating of glacial and associated sediments: review, recommendations and future directions. *Boreas* **37**, 636-659, doi:10.1111/j.1502-3885.2008.00052.x (2008).
86 DeLong, S. B. & Arnold, L. J. Dating alluvial deposits with optically stimulated luminescence, AMS C-14 and cosmogenic techniques, western Transverse Ranges, California, USA. *Quaternary Geochronology* **2**, 129-136, doi:10.1016/j.quageo.2006.03.012 (2007).
87 Fattahi, M. *et al.* Refining the OSL age of the last earthquake on the Dheshir fault, Central Iran. *Quaternary Geochronology* **5**, 286-292, doi:10.1016/j.quageo.2009.04.005 (2010).
88 Costas, I. *et al.* Comparison of OSL ages from young dune sediments with a high-resolution independent age model. *Quaternary Geochronology* **10**, 16-23, doi:10.1016/j.quageo.2012.03.007 (2012).
89 Stone, A. E. C., Thomas, D. S. G. & Viles, H. A. Late Quaternary palaeohydrological changes in the northern Namib Sand Sea: New chronologies using OSL dating of interdigitated aeolian and water-lain interdune deposits. *Palaeogeography Palaeoclimatology Palaeoecology* **288**, 35-53, doi:10.1016/j.palaeo.2010.01.032 (2010).
90 Gaar, D. & Preusser, F. Luminescence dating of mammoth remains from northern Switzerland. *Quaternary Geochronology* **10**, 257-263, doi:10.1016/j.quageo.2012.02.007 (2012).
91 Thomsen, K. J., Jain, M., Bøtter-Jensen, L., Murray, A. S. & Jungner, H. Variation with depth of dose distributions in single grains of quartz extracted from an irradiated concrete block. *Radiation Measurements* **37**, 315-321, doi:10.1016/s1350-4487(03)00006-4 (2003).
92 Rodnight, H., Duller, G. A. T., Wintle, A. G. & Tooth, S. Assessing the reproducibility and accuracy of optical dating of fluvial deposits. *Quaternary Geochronology* **1**, 109-120, doi:10.1016/j.quageo.2006.05.017 (2006).
93 Galbraith, R. F. *Statistics for Fission Track Analysis*. (Chapman & Hall, 2005).
94 Roberts, R. G., Galbraith, R. F., Yoshida, H., Laslett, G. M. & Olley, J. M. Distinguishing dose populations in sediment mixtures: a test of single-grain optical dating procedures using mixtures of laboratory-dosed quartz. *Radiation Measurements* **32**, 459-465, doi:10.1016/s1350-4487(00)00104-9 (2000).
95 Arnold, L. J., Demuro, M., Navazo, M., Benito-Calvo, A. & Perez-Gonzalez, A. OSL dating of the Middle Palaeolithic Hotel California site, Sierra de Atapuerca, north-central Spain. *Boreas* **42**, 285-305, doi:10.1111/j.1502-3885.2012.00262.x (2013).
96 Armitage, S. J. *et al.* The Southern Route "Out of Africa": Evidence for an Early Expansion of Modern Humans into Arabia. *Science* **331**, 453-456, doi:10.1126/science.1199113 (2011).
97 Jacobs, Z. *et al.* Ages for the Middle Stone Age of Southern Africa: Implications for Human Behavior and Dispersal. *Science* **322**, 733-735, doi:10.1126/science.1162219 (2008).
98 Jacobs, Z. *et al.* Single-grain OSL dating at La Grotte des Contrebandiers ('Smugglers' Cave'), Morocco: improved age constraints for the Middle Paleolithic levels. *Journal of Archaeological Science* **38**, 3631-3643, doi:10.1016/j.jas.2011.08.033 (2011).

99 Jacobs, Z., Duller, G. A. T. & Wintle, A. G. Interpretation of single grain D-e distributions and calculation of D-e. *Radiation Measurements* **41**, 264-277, doi:10.1016/j.radmeas.2005.07.027 (2006).
100 David, B. *et al.* Sediment mixing at Nonda Rock: investigations of stratigraphic integrity at an early archaeological site in northern Australia and implications for the human colonisation of the continent. *Journal of Quaternary Science* **22**, 449-479, doi:10.1002/jqs.1136 (2007).
101 Bateman, M. D. *et al.* Detecting post-depositional sediment disturbance in sandy deposits using optical luminescence. *Quaternary Geochronology* **2**, 57-64, doi:10.1016/j.quageo.2006.05.004 (2007).
102 Jacobs, Z., Wintle, A. G., Duller, G. A. T., Roberts, R. G. & Wadley, L. New ages for the post-Howiesons Poort, late and final Middle Stone Age at Sibudu, South Africa. *Journal of Archaeological Science* **35**, 1790-1807, doi:10.1016/j.jas.2007.11.028 (2008).
103 Jacobs, Z., Wintle, A. G., Roberts, R. G. & Duller, G. A. T. Equivalent dose distributions from single grains of quartz at Sibudu, South Africa: context, causes and consequences for optical dating of archaeological deposits. *Journal of Archaeological Science* **35**, 1808-1820, doi:10.1016/j.jas.2007.11.027 (2008).
104 Galbraith, R. F. On the mis-use of mathematics: A comment on "How confident are we about the chronology of the transition between Howieson's Poort and Still Bay?" by Guerin et al. (2013). *Journal of Human Evolution* **80**, 184-186, doi:10.1016/j.jhevol.2014.10.006 (
105 Jacobs, Z. & Roberts, R. G. Single-grain OSL chronologies for the Still Bay and Howieson's Poort industries and the transition between them: Further analyses and statistical modelling *Journal of Human Evolution* **107**, 1-13 (2017).
106 Huntley, D. J. & Lian, O. B. Some observations on tunnelling of trapped electrons in feldspars and their implications for optical dating. *Quaternary Science Reviews* **25**, 2503-2512, doi:10.1016/j.quascirev.2005.05.011 (2006).
107 Moska, P. & Murray, A. S. Stability of the quartz fast-component in insensitive samples. *Radiation Measurements* **41**, 878-885, doi:10.1016/j.radmeas.2006.06.005 (2006).
108 Fujita, H., Jain, M. & Murray, A. S. Retrospective dosimetry using Japanese brick quartz: A way forward despite an unstable fast decaying OSL signal. *Radiation Measurements* **46**, 565-572, doi:10.1016/j.radmeas.2011.03.025 (2011).
109 Fan, A. C., Li, S. H. & Li, B. Observation of unstable fast component in OSL of quartz. *Radiation Measurements* **46**, 21-28, doi:10.1016/j.radmeas.2010.10.001 (2011).
110 Wintle, A. G. & Murray, A. S. Towards the development of a preheat procedure for OSL dating of quartz. *Radiation Measurements* **29**, 81-94, doi:10.1016/s1350-4487(97)00228-x (1998).
111 Vannay, J. C. *et al.* Miocene to Holocene exhumation of metamorphic crustal wedges in the NW Himalaya: Evidence for tectonic extrusion coupled to fluvial erosion. *Tectonics* **23**, doi:Tc1014 10.1029/2002tc001429 (2004).
112 Webb, A. A. G. *et al.* Cenozoic tectonic history of the Himachal Himalaya (northwestern India) and its constraints on the formation mechanism of the Himalayan orogen. *Geosphere* **7**, 1013-1061, doi:10.1130/ges00627.1 (2011).
113 White, N. M. *et al.* Constraints on the exhumation and erosion of the High Himalayan Slab, NW India, from foreland basin deposits. *Earth and Planetary Science Letters* **195**, 29-44 (2002).

114 Yin, A. Cenozoic tectonic evolution of the Himalayan orogen as constrained by along-strike variation of structural geometry, exhumation history, and foreland sedimentation. *Earth-Science Reviews* **76**, 1-131, doi:10.1016/j.earscirev.2005.05.004 (2006).
115 Gehrels, G. *et al.* Detrital zircon geochronology of pre-Tertiary strata in the Tibetan-Himalayan orogen. *Tectonics* **30**, TC5016, doi:5010.1029/2011TC00286, doi:10.1029/2011tc002868 (2011).
116 DeCelles, P. G. *et al.* Detrital geochronology and geochemistry of Cretaceous-Early Miocene strata of Nepal: implications for timing and diachroneity of initial Himalayan orogenesis. *Earth and Planetary Science Letters* **227**, 313-330, doi:10.1016/j.epsl.2004.08.019 (2004).
117 Ravikant, V., Wu, F. Y. & Ji, W. Q. U-Pb age and Hf isotopic constraints of detrital zircons from the Himalayan foreland Subathu sub-basin on the Tertiary palaeogeography of the Himalaya. *Earth and Planetary Science Letters* **304**, 356-368, doi:10.1016/j.epsl.2011.02.009 (2011).
118 Vermeesch, P. Multi-sample comparison of detrital age distributions (vol 341, pg 140, 2013). *Chemical Geology* **425**, 145-145, doi:10.1016/j.chemgeo.2016.02.005 (2016).
