# Supplementary material for: Counter-intuitive influence of Himalayan river morphodynamics on Indus Civilisation urban settlements
Source: Nat Commun. 2017 Nov 28;8:1617. doi: 10.1038/s41467-017-01643-9 (PMC5705636; doi:10.1038/s41467-017-01643-9)
Supplement: Supplementary file 2 — Description of Additional Supplementary Files [file 41467_2017_1643_MOESM2_ESM.pdf]

Description of Additional Supplementary Files

File Name: Supplementary Data 1
Description: Detrital zircon U-Pb_isotope_data for modern river, dune and core samples

File Name: Supplementary Data 1
Description: Detrital mica Ar-Ar isotope_data for modern river and core samples
